# METTL4-mediated nuclear N6-deoxyadenosine methylation promotes metastasis through activating multiple metastasis-inducing targets

**DOI:** 10.1186/s13059-022-02819-3

**Published:** 2022-12-02

**Authors:** Kai-Wen Hsu, Joseph Chieh-Yu Lai, Jeng-Shou Chang, Pei-Hua Peng, Ching-Hui Huang, Der-Yen Lee, Yu-Cheng Tsai, Chi-Jung Chung, Han Chang, Chao-Hsiang Chang, Ji-Lin Chen, See-Tong Pang, Ziyang Hao, Xiao-Long Cui, Chuan He, Kou-Juey Wu

**Affiliations:** 1 Cancer Genome Research Center, Chang Gung Memorial Hospital at Linkou, No. 15, Wenhua 1st Road, Gueishan Dist., Taoyuan, 333 Taiwan; 2 Research Center for Cancer Biology, Taipei, Taiwan; 3 Institute of Translational Medicine and New Drug Development, China Medical University, Taichung, 404 Taiwan; 4 Institute of Biomedical Sciences, China Medical University, Taichung, 404 Taiwan; 5 Institute of Integrated Medicine, China Medical University, Taichung, 404 Taiwan; 6 Department of Health Risk Management, College of Public Health, China Medical University, Taichung, 404 Taiwan; 7 Department of Pathology, China Medical University Hospital, Taichung, 404 Taiwan; 8 Department of Urology, China Medical University Hospital, Taichung, 404 Taiwan; 9 Comprehensive Breast Health Center, Taipei Veterans General Hospital, Taipei, 112 Taiwan; 10 Division of Urology, Department of Surgery, Chang Gung Memorial Hospital at Linkou, Taoyuan, 333 Taiwan; 11 Departments of Chemistry & Biochemistry and Molecular Biology, Institute for Biophysical Dynamics, The University of Chicago, 929 E. 57th St., Chicago, IL 60637 USA; 12 School of Pharmaceutical Sciences, Capital Medical University, Beijing, 100069 China; 13 Howard Hughes Medical Institute, The University of Chicago, 929 E. 57th St., Chicago, IL 60637 USA

**Keywords:** METTL4, 6mA, Hypoxia, lncRNA, ZMIZ1, Metastasis

## Abstract

**Background:**

DNA N6-methyldeoxyadenosine (6mA) is rarely present in mammalian cells and its nuclear role remains elusive.

**Results:**

Here we show that hypoxia induces nuclear 6mA modification through a DNA methyltransferase, METTL4, in hypoxia-induced epithelial-mesenchymal transition (EMT) and tumor metastasis. Co-expression of METTL4 and 6mA represents a prognosis marker for upper tract urothelial cancer patients. By RNA sequencing and 6mA chromatin immunoprecipitation-exonuclease digestion followed by sequencing, we identify lncRNA RP11-390F4.3 and one novel HIF-1α co-activator, ZMIZ1, that are co-regulated by hypoxia and METTL4. Other genes involved in hypoxia-mediated phenotypes are also regulated by 6mA modification. Quantitative chromatin isolation by RNA purification assay shows the occupancy of lncRNA RP11-390F4.3 on the promoters of multiple EMT regulators, indicating lncRNA-chromatin interaction. Knockdown of lncRNA RP11-390F4.3 abolishes METTL4-mediated tumor metastasis. We demonstrate that ZMIZ1 is an essential co-activator of HIF-1α.

**Conclusions:**

We show that hypoxia results in enriched 6mA levels in mammalian tumor cells through METTL4. This METTL4-mediated nuclear 6mA deposition induces tumor metastasis through activating multiple metastasis-inducing genes. METTL4 is characterized as a potential therapeutic target in hypoxic tumors.

**Supplementary Information:**

The online version contains supplementary material available at 10.1186/s13059-022-02819-3.

## Background

DNA *N*^6^-dexoyadenosine methylation (6mA or m^6^dA) is prevalent in prokaryote genomes that mainly plays a role in bacterial host defense, DNA replication, repair, transposition, and gene regulation [1]. 6mA has been shown to exist in unicellular eukaryotes (ciliates and green algae), *Caenorhabditis elegans* and *Drosophila* [2–5]. 6mA also exists in vertebrates, including *Xenopus laevis*, zebrafish, mouse, pig, and human [6–9]. 6mA distribution is species- and developmental stage-specific [3–5, 7–10], and may be induced following different stress conditions [11–14]. The presence of 6mA in various genomic locations could correlate with different outcomes of gene expression and phenotypes [15, 16]. For example, 6mA is linked to transcriptionally active genes in early-diverging fungi, *Arabidopsis*, and humans due to its clustering around transcription start sites (TSS) [9, 10, 17]. For tumor biology, abundant 6mA levels have been observed in glioblastoma patient samples [18]. The enzyme that mediates genomic 6mA deposition has been shown to be DAMT-1 in *C. elegans* [4, 13] and METTL4 in mammalian cells [19, 20]. Recent results showed that mitochondria DNA (mtDNA) is enriched for 6mA and METTL4 mediates mtDNA 6mA methylation [11]. Hypoxia further elevates the mtDNA 6mA levels that regulate mitochondrial stress response [11]. However, the nuclear role of METTL4 and 6mA depositions in gene expression and tumorigenesis in mammalian cells remain unknown.

Hypoxia is a tumor microenvironmental factor that facilitates cancer metastasis and tumor progression [21]. Induction of tumor progression by hypoxia/HIF-1α could occur using various mechanisms, including transcriptional and epigenetic mechanisms [21–23]. Multiple types of human tumors with hypoxia contribution have been demonstrated [21]. However, effective treatment options against hypoxia-involved tumors are still under intensive investigation, although a therapeutic inhibitor against HIF-2α has been developed [24]. The alternative strategy will be to develop therapeutic agents against hypoxia-induced targets, especially epigenetic targets, that promote tumor metastasis [25, 26].

Long noncoding RNAs (lncRNAs) are long (>200 nt) RNAs that lack protein-coding sequences [27]. LncRNAs regulate crucial biological processes, including cell differentiation, lineage determination, organ development, and tumorigenesis [27, 28]. Their expression could be disease- or tumor-specific [29, 30]. LncRNAs have been shown to globally control gene expression through chromatin scaffolding [31]. Numerous tumor-inducing lncRNAs in human cancers have been demonstrated [31, 32]. However, 6mA-regulated lncRNAs that could control the expression of multiple metastasis-inducing factors remain relatively unknown.

In this study, we show that 6mA levels are enriched inside nucleus under hypoxia in mammalian tumor cells. METTL4 mediates nuclear 6mA depositions and its overexpression in tumor cells induces the epithelial-mesenchymal transition (EMT) [33, 34] and tumor metastasis. METTL4-mediated nuclear 6mAs lead to the activation of a lncRNA that activates core EMT transcription regulators, an unidentified HIF-1α co-activator, and various HIF-1α target genes that contribute to hypoxia-induced phenotypes. Therefore, METTL4-mediated nuclear 6mA deposition inside nucleus controls hypoxia-induced gene expression and tumor metastasis through activating multiple metastasis-inducing targets, identifying METTL4 as a therapeutic target for hypoxia-involved tumors.

## Results

### Induction of nuclear DNA N^6^-deoxyadenosine methylation by METTL4 under hypoxia

**Fig. 1:**
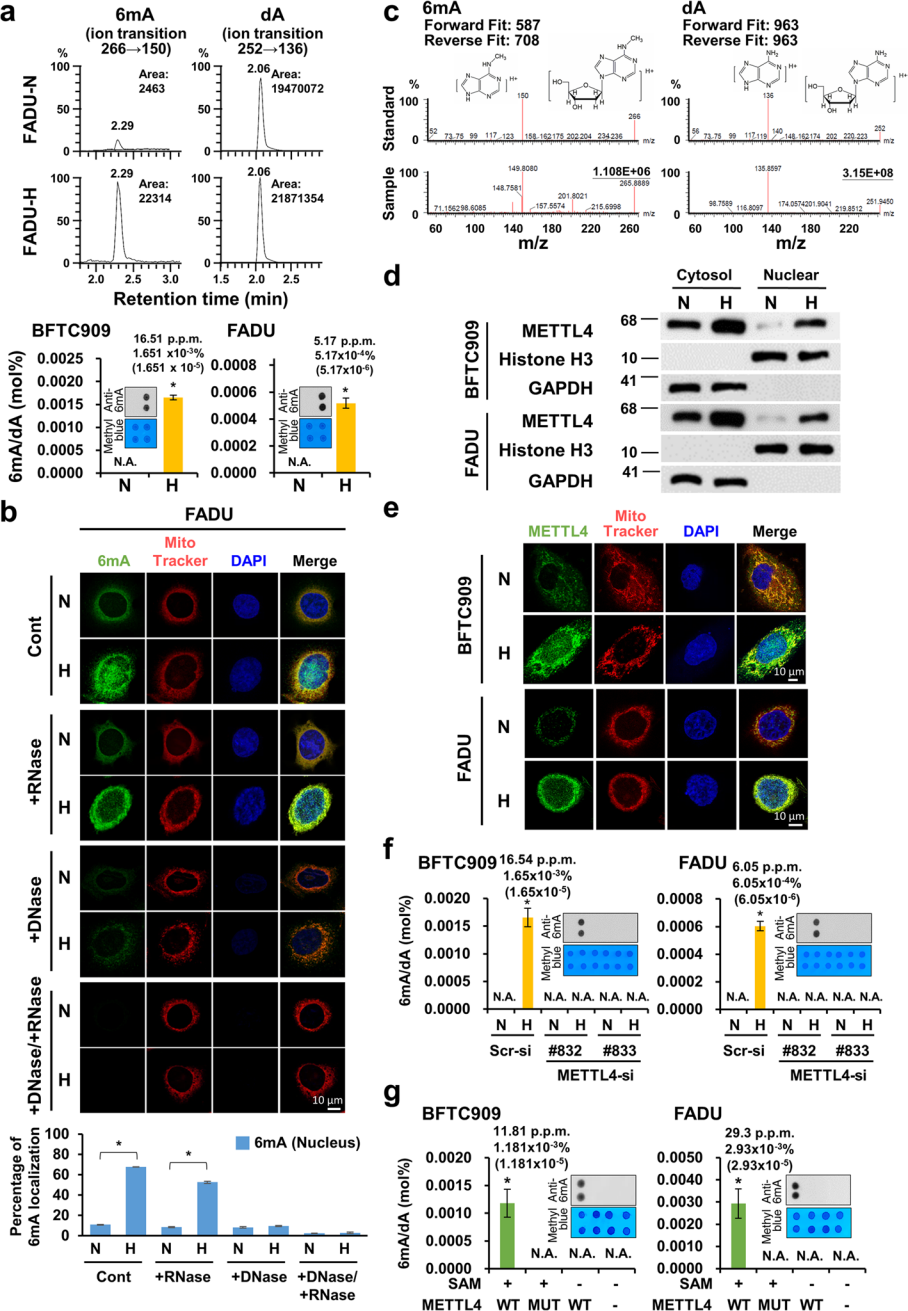
Increase in the nuclear 6mA levels through nuclear activation of METTL4 expression under hypoxia. **a** An increase in nuclear 6mA levels was observed in BFTC909 and FADU cells under hypoxia (see “Methods”). The collected results were summarized as the ratios of 6mA/dA in the bar graph (lower panel). Corresponding 6mA dot blots with methyl blue loading controls are shown together with bar graphs. N, normoxia; H, hypoxia. Normoxic condition was used as a control. **b** Immunofluorescence staining shows the increased nuclear staining of 6mA in FADU cells under hypoxia. Staining of cell nuclei by DAPI and mitochondria by MitoTracker was used as controls. Bar graph indicated the percentage of cells containing nuclear 6mA signals. **c** Detected 6mA and dA in METTL4-induced gDNAs were verified by product ion conformation spectra (PICS) fit to the spectrum generated from each standard. Parental ion of 6mA was m/z 266 with major daughter ion m/z 150. Parental ion of dA was m/z 252 with major daughter ion m/z 136. **d** Western blot analysis shows the more prominent nuclear activation of METTL4 levels through nuclear fractionation in two different cell lines. Histone H3 and GAPDH was used as a nuclear and cytoplasmic control, respectively. **e** Immunofluorescence staining shows the increased nuclear METTL4 expression in cells under hypoxia in BFTC909 and FADU cells. Mitotracker: mitochondria DNA. Cell nuclei were stained by DAPI. **f** Knockdown of *METTL4* abolished the increase in nuclear 6mA levels induced by hypoxia in BFTC909 and FADU cells. Corresponding 6mA dot blots are shown. **g** In vitro DNA methylation assays show an increase in the 6mA levels by incubating METTL4 with genomic DNAs from BFTC909 or FADU cells. The METTL4 mutant and incubation without SAM were used as controls. Corresponding 6mA dot blots are shown. N, normoxia; H, hypoxia. Normoxic condition was used as a control. The asterisk (*) indicated statistical significance (*P*<0.05) between experimental and control groups

To test the role of 6mA inside the nucleus of mammalian cells, we first examined the nuclear 6mA levels. Since 6mA levels have been shown to be very low or undetectable in the nucleus of mammalian cells and may be induced following different stress conditions [11–14], we further tested whether other stress conditions, including heat shock, oxidative stress, acidosis, serum starvation, or hypoxia could induce a significant increase in the 6mA levels inside the nucleus. The DNAs purified from cells were first depleted of mitochondria DNAs by a conventional mitochondria depletion method followed by extensive RNase treatment to avoid m^6^A RNA contamination (see “Methods”). We performed ultra-performance liquid chromatography with electrospray ionization tandem mass spectrometry **(**UPLC-ESI-MS/MS) analysis of nuclear 6mA levels in mammalian cells under various stress conditions [5]. The calibration standards and the curves of quantifying 6mA levels were shown (Additional file 1: Fig. S1a-b) [4, 5, 8, 11]. Among the various stress conditions, only hypoxia induced detectable 6mA levels inside the nucleus (Additional file 1: Fig. S1c). Genomic 6mA levels increased from undetectable levels to around 5 to 16 ppm under hypoxia in two cell lines, FADU (head and neck cancer) and BFTC909 (upper tract urothelial cancer-UTUC) (Fig. 1a; Additional file 1: Fig. S1c). Increased 6mA levels were also induced by hypoxia in a primary UTUC cell line, KTCC28M (Additional file 1: Fig. S1d). For control experiments, we first tested whether the genomic DNA extraction step of mitochondria depletion was enough to deplete the mitochondria DNA (see “Methods”). The results showed that no mitochondrial DNAs were detected in three different cell lines after mitochondria depletion from the genomic DNA extraction step (Additional file 1: Fig. S1e). We also tested the possible existence of contaminating 6mAs inside all the reagents and the results again showed no detectable 6mA levels in all the reagents (Additional file 1: Fig. S1f). Before using immunofluorescence staining to detect the presence of 6mAs, antigen retrieval step was tested and it did not affect the immunofluorescence staining results (Additional file 1: Fig. S1g). The staining of 6mA inside nucleus by anti-6mA antibodies significantly increased under hypoxia in FADU, BFTC909, and KTCC28M cells (Fig. 1b, upper panels; Additional file 1: Fig. S1h). Nuclear 6mA staining remained after RNase treatment (Fig. 1b, middle panels; Additional file 1: Fig. S1h). Treatment with DNase showed that the induced 6mA signals were abolished (Fig. 1b, middle panels; Additional file 1: Fig. S1h). After treatment with DNase and RNase, the induced 6mA signals disappeared (Fig. 1b, lower panels; Additional file 1: Fig. S1h). The percentage of cells containing nuclear or cytoplasmic 6mA signals were shown in a bar graph (Fig. 1b, lower panel; Additional file 1: Fig. S1i), indicating the 6mA signals detected by the anti-6mA antibodies were DNA-specific. MS/MS fragmentation profiling confirmed the identity of increased 6mA levels detected (Fig. 1c), whereas no 6mAs were detected in all the reagents tested (Additional file 1: Fig. S1j). All these results indicate an increase in nuclear 6mA levels induced by hypoxia.

Since METTL4 has been shown to be a putative DNA 6mA methyltransferase [11, 19, 20], we tested whether METTL4 could also be activated by hypoxia inside the nucleus. Indeed, total METTL4 levels (mRNA and protein) were increased under hypoxia in three cell lines (Additional file 1: Fig. S1k). Fractionation experiments showed that nuclear METTL4 levels significantly increased under hypoxia (Fig. 1d). Immunofluorescence staining using the anti-METTL4 antibodies showed the increased staining of nuclear METTL4 under hypoxic condition compared to the very low levels of nuclear METTL4 staining under normoxic condition (Fig. 1e; Additional file 1: Fig. S1l). We tested the role of METTL4 in mediating nuclear 6mA modification. The increase in nuclear 6mA levels under hypoxia was abolished by METTL4 knockdown using UPLC-ESI-MS/MS analysis (Fig. 1f). Overexpression of METTL4, but not the enzymatically inactive mutant, increased the 6mA levels in two cell lines using UPLC-ESI-MS/MS analysis (Additional file 1: Fig. S1m). The specificity of the anti-METTL4 antibody was confirmed by immunofluorescence staining experiments showing the staining of METTL4 under hypoxia and abolishment of METTL4 staining under METTL4 knockdown in three different cell lines (Additional file 1: Fig. S1n). Finally, in vitro methylation assays using purified METTL4 showed the ability of METTL4 to deposit 6mA in the genomic DNAs (gDNAs) of BFTC909 and FADU cells (Fig. 1g). We also tested whether another putative 6mA methyltransferase, N6AMT1, could mediate 6mA modification [9]. The results showed that the induction of 6mA by hypoxia still maintained in the presence of N6AMT1 knockdown, indicating that N6AMT1 was not a 6mA methyltransferase (Additional file 1: Fig. S1o, p). These results indicate that hypoxia increases nuclear 6mA levels that are mediated by nuclear METTL4.

Since it has been reported that METTL4 could mediate *U2 snRNA* m^6^Am modification [35, 36], we tested this activity. Our results showed that METTL4 mediated *U2 snRNA* m^6^Am modification and increased the m^6^Am levels about 50% under hypoxic condition (Additional file 1: Fig. S1q). Overexpression of METTL4, but not the enzymatically inactive mutant, increased the m^6^Am levels in two cell lines using UPLC-ESI-MS/MS analysis (Additional file 1: Fig. S1r). In vitro RNA methylation assays also showed that mutation of the enzymatic site of METTL4 abolished its *U2 snRNA* m^6^Am modifying activity (Additional file 1: Fig. S1s). Other characterizations of the m^6^Am-modifying effects mediated by METTL4 are shown in the later section (Additional file 1: Fig. S4).

Although nuclear and cytoplasmic METTL4 levels were induced by hypoxia (Fig. 1d), the precise molecular mechanism that regulates METTL4 expression by hypoxia is unknown. We further determined the activation mechanism of METTL4 expression by hypoxia. Overexpression of a constitutively active HIF-1α mutant activated METTL4 expression (Additional file 1: Fig. S1t). Knockdown of *HIF-1*α abolished the activation of METTL4 by hypoxia, indicating that HIF-1α is a crucial regulator of METTL4 expression (Additional file 1: Fig. S1u). Reporter gene assays showed that HIF-1α activated the *METTL4* promoter-driven reporter construct and a HRE (hypoxia response element) located in the proximal promoter of *METTL4* was identified (Additional file 1: Fig. S1v). Chromatin immunoprecipitation (ChIP) assays showed the direct binding of HIF-1α to the proximal promoter of *METTL4* (Additional file 1: Fig. S1w). We also tested whether HIF-2α plays a role in the activation of METTL4 expression. The results showed that knockdown of *HIF-2*α under hypoxia did not abolish the activation of METTL4 by hypoxia, indicating that HIF-2α was not responsible for hypoxia-induced METTL4 activation (Additional file 1: Fig. S1x). We further tested the role of HIF-1β due to its dimerization ability with HIF-1α. Similar to the role of HIF-1α, knockdown of *HIF-1β* abolished the activation of METTL4 by hypoxia, indicating that heterodimerization of HIF-1β with HIF-1α was essential in hypoxia-induced METTL4 activation (Additional file 1: Fig. S1x). Actinomycin D experiments were also used to test the transcriptional activation of *METTL4* by hypoxia, and the results showed that treatment of Actinomycin D significantly decreased the activation of METTL4 (at the mRNA and protein levels) by hypoxia (Additional file 1: Fig. S1y, z), indicating that METTL4 induction by hypoxia goes through transcriptional regulation. These results demonstrate that HIF-1α directly activates the expression of *METTL4*.

### METTL4 is essential in the induction of hypoxia-induced epithelial-mesenchymal transition

**Fig. 2:**
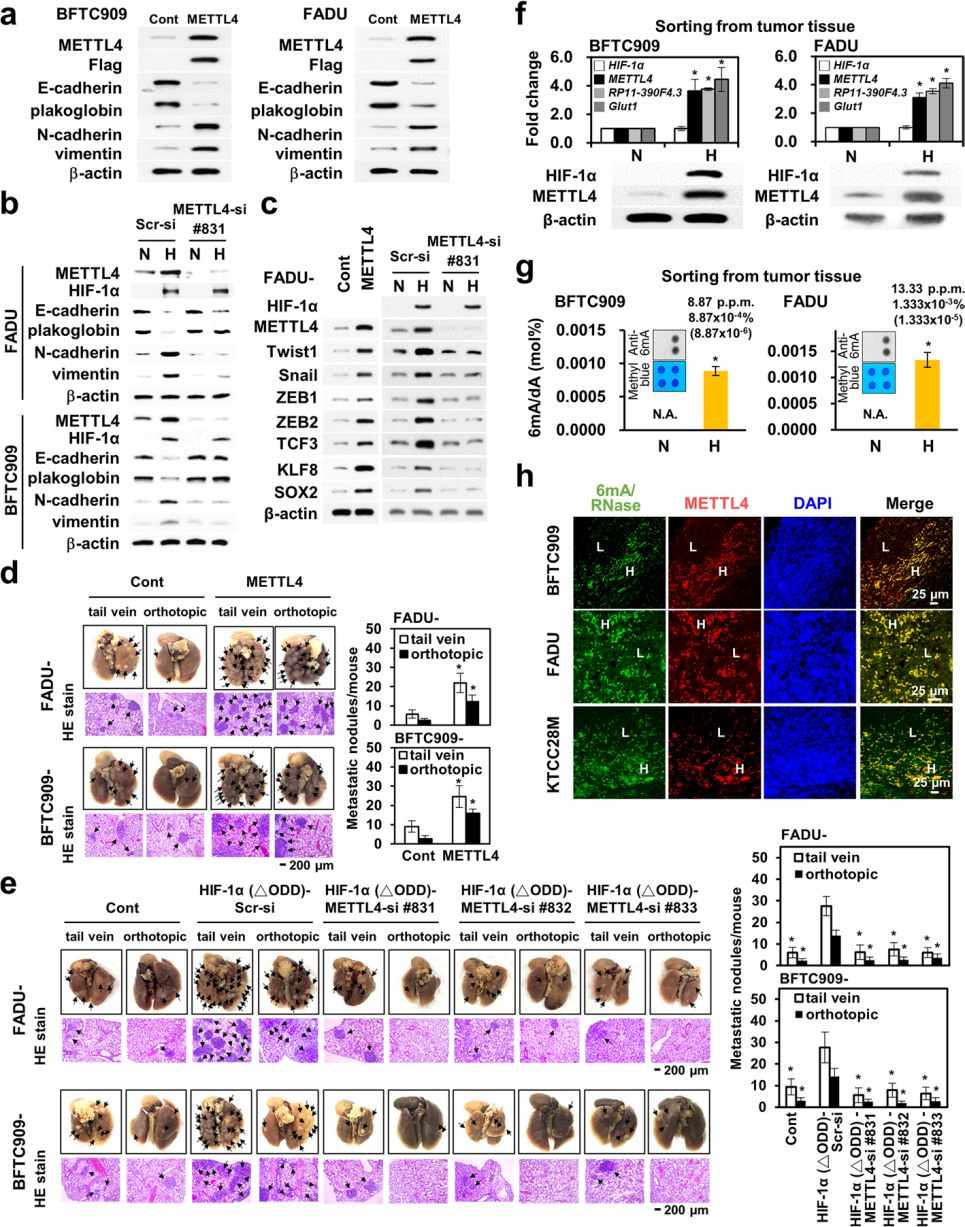
METTL4 is essential in hypoxia-induced EMT and in vitro*/*in vivo metastatic activity. **a** Overexpression of METTL4 induced EMT in BFTC909 and FADU cells by Western blot analysis. The cell clone transfected with the control vector was used as a control. **b** Knockdown of *METTL4* reversed the expression of EMT markers regulated by hypoxia in BFTC909 and FADU cells. **c** Overexpression of METTL4 induced the expression of a set of EMT transcriptional regulators and knockdown of *METTL4* abolished the activation of these EMT regulators induced by hypoxia. **d** Overexpression of METTL4 induced increased numbers of metastatic lung nodules in mice in tail vein and orthotopic implantation experiments. Representative gross anatomy and histology are shown on the left; measurement of metastatic lung nodules is shown on the right. **e** Knockdown of *METTL4* significantly decreased the increased metastatic lung nodules in mice injected with cells overexpressing a HIF-1α constitutively active mutant. Representative gross anatomy and histology are shown on the left; measurement of metastatic lung nodules is shown on the right. **f** Hypoxic tumor cells sorted from xenografted tumors from BFTC909 and FADU cells show the increased HIF-1α and METTL4 protein levels together with increased HIF-1α target gene expression. *Glut1* activation was used as a positive control. N, normoxia; H, hypoxia. Normoxic cells were used as a control. **g** Hypoxic tumor cells sorted from xenografted tumors from BFTC909 and FADU cells show an increase in the 6mA levels. Corresponding 6mA dot blots with methyl blue loading controls are shown together with bar graphs. N, normoxia; H, hypoxia. **h** Immunofluorescence staining shows the co-staining of 6mA and METTL4 in xenografted tumors from BFTC909, FADU, and KTCC28M cells. Green fluorescence: 6mA staining (RNase treatment); red fluorescence: METTL4 staining. Cell nuclei were stained by DAPI. H, highly colocalized area; L, less colocalized area. The asterisk (*) indicated statistical significance (*P*<0.05) between experimental and control groups

To determine the role of METTL4 in tumorigenesis, we tested the role of METTL4 in hypoxia-induced tumor progression since hypoxia/HIF-1α induces the epithelial-mesenchymal transition (EMT) and tumor metastasis [33, 34, 37, 38]. Overexpression of the wild type METTL4 induced EMT by Western blot and immunofluorescence staining assays in two cell lines (Fig. 2a; Additional file 1: Fig. S2a). Hypoxia-induced EMT was abolished after *METTL4* knockdown using three different siRNA vectors by Western blot analysis in three different cell lines (Fig. 2b; Additional file 1: Fig. S2b, c). Immunofluorescence staining results of E-cadherin and vimentin were consistent with the Western blot results in two cell lines (Additional file 1: Fig. S2d). The in vitro migration and invasion activity of tumor cells were induced by METTL4 overexpression in two cell lines (Additional file 1: Fig. S2e). The increased in vitro migration and invasion activity induced by hypoxia was decreased by METTL4 knockdown using three different siRNA vectors in three different cell lines (Additional file 1: Fig. S2f). METTL4 overexpression or hypoxia activated the expression of various EMT transcriptional regulators, including Twist1, Snail, ZEB1, ZEB2, TCF3, KLF8, and SOX2 in two different cell lines (Fig. 2c; Additional file 1: Fig. S2g). The expression of hypoxia-activated EMT transcriptional regulators was abolished under *METTL4* knockdown using three different siRNA vectors (Fig. 2c; Additional file 1: Fig. S2g). To further confirm the above results, knockdown of *METTL4* in a lung cancer H1299 cell line constitutively overexpressing HIF-1α [38] showed the reversion of EMT, a decrease in the expression of EMT regulators, and a decrease in the in vitro migration and invasion activity (Additional file 1: Fig. S2h, i). In addition, we tested two glycolysis genes, *Glut1* and *REDD1*, that were activated by hypoxia and their regulation by METTL4. Hypoxia activated the expressions of *Glut1* and *REDD1* and their expressions were abolished under *METTL4* knockdown in FADU and BFTC909 cells (Additional file 1: Fig. S2j). Knockdown of METTL4 decreased the expressions of *Glut1* and *REDD1* in H1299 cells (Additional file 1: Fig. S2j). We also tested the ability of METTL4 to regulate cell proliferation [11]. Overexpression of METTL4 in three cell lines increased cell proliferation rate (Additional file 1: Fig. S2k). Knockdown of METTL4 in three cell lines under hypoxic condition decreased their proliferation rate (Additional file 1: Fig. S2l). Knockdown of METTL4 followed by reconstitution with a wild type METTL4 or a nuclear localization-defective mutant METTL4 showed that the will type METTL4 fully rescued the cell proliferation rate, whereas the nuclear localization-defective mutant METTL4 only partially rescued cell proliferation rate (Additional file 1: Fig. S2m, Fig. S3e-g). These results supported the role of METTL4 in cell proliferation.

To test the in vivo tumorigenic ability of METTL4, we showed that METTL4 overexpression increased the tumor volume of tumor cells from three different tumor cell lines (Additional file 1: Fig. S2n). HIF-1α overexpression induced an increase in tumor volume in three different cell lines, and the HIF-1α-induced increase in tumor volume was abolished by knockdown of *METTL4* using three different siRNA vectors (Additional file 1: Fig. S2o). Since METTL4 overexpression activates various EMT regulators and increased the in vitro migration and invasion activity, we further tested its in vivo metastatic activity. Overexpression of METTL4 in two different cell lines increased their in vivo metastatic activity by using either tail vein or orthotopic implantation assays (Fig. 2d). Other in vivo experiments showed that METTL4 knockdown abolished the in vivo metastatic activity induced by overexpression of a constitutively active HIF-1α mutant in two different cell lines (Fig. 2e). The above results indicate the crucial role of METTL4 in hypoxia/HIF-1α -induced metastatic activity in vitro and in vivo.

From the mice xenograft experiments performed, we further isolated hypoxic cells by Pimonidazole staining through FACS from the xenografted tumors and examined the correlation between HIF-1α, 6mA, and METTL4 expression. The results showed that indeed only hypoxic tumor cells (FADU, BFTC909, KTCC28M) staining with Pimonidazole (a representative sorting was shown in Additional file 1: Fig. S2p) has increased METTL4 expression and increased 6mA levels (Fig. 2f, g; Additional file 1: Fig. S2q). Immunofluorescence staining also showed the correlation between HIF-1α and METTL4, HIF-1α and 6mA, and METTL4 and 6mA in hypoxic FADU, BFTC909, and KTCC28M cells (Fig. 2h; Additional file 1: Fig. S2r).

**Fig. 3:**
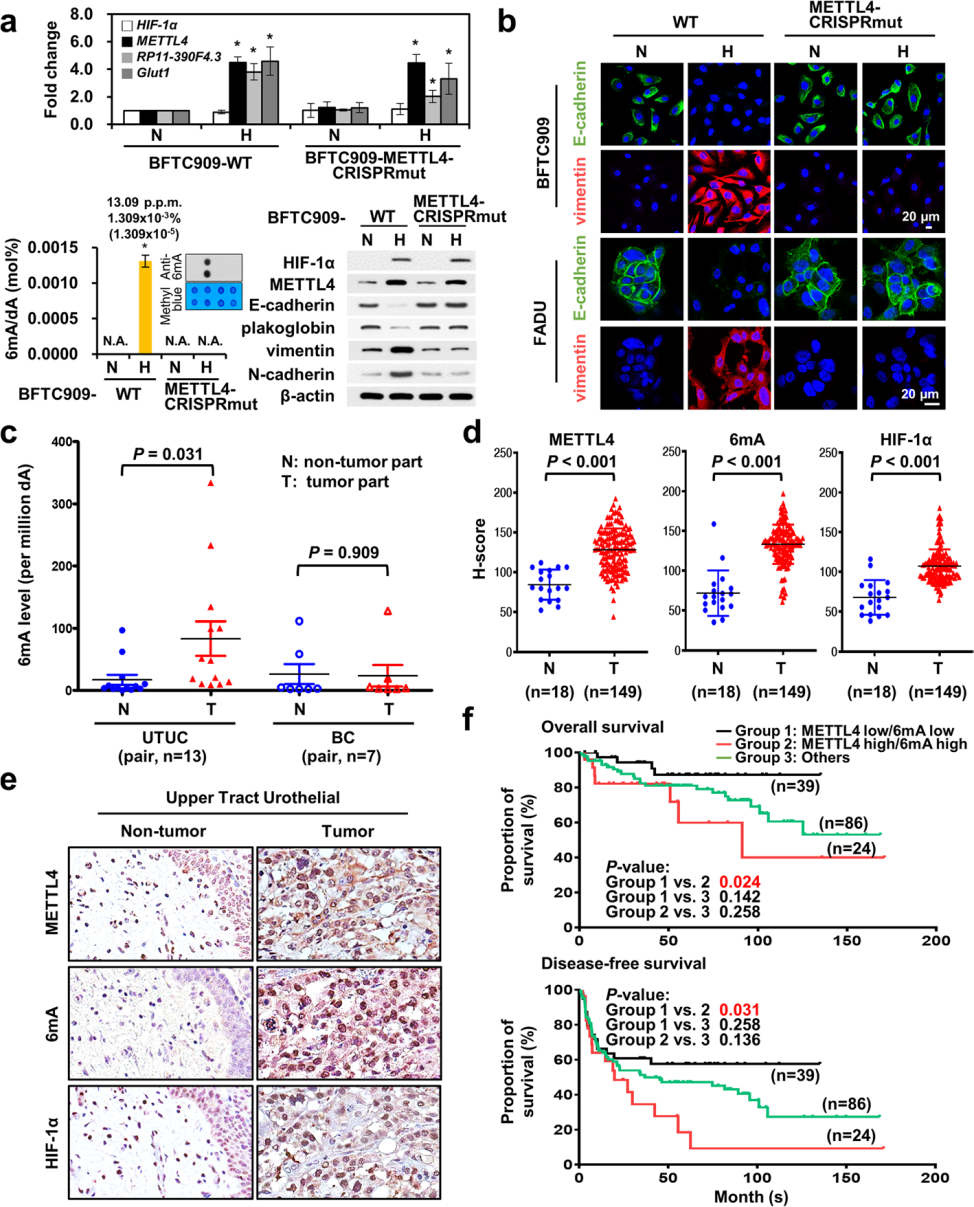
The essential role of the enzymatic activity of METTL4 in hypoxia-induced phenotypes and clinical implications. **a** Mutation of the enzymatic site of METTL4 by a prime-cutting CRISPR-Cas9 approach in BFTC909 cells abolished the induction of EMT by hypoxia and significantly decreased the RNA expression of *RP11-390F4.3* and *Glut1*. The induction of 6mA levels was abolished in enzymatically inactive METTL4 mutant BFTC909 cells. N, normoxia; H, hypoxia. The normoxic condition for METTL4 wild type BFTC909 cells was used as a control. A corresponding 6mA dot blot with methyl blue loading control is shown together with the bar graph. The asterisk (*) indicated statistical significance (*P*<0.05) between experimental and control groups. **b** Immunofluorescence staining shows the abolishment of EMT induction by hypoxia in the enzymatically inactive METTL4 mutant FADU and BFTC909 cells. Green fluorescence represented staining of E-cadherin; red fluorescence represented staining of vimentin. Cell nuclei were stained by DAPI. N, normoxia; H, hypoxia. The normoxic condition for METTL4 wild type BFTC909 and FADU cells were used as a control. **c** Increased 6mA levels in UTUC, but not in bladder cancer (BC), patient samples. **d** Increased METTL4, 6mA, and HIF-1α levels by immunohistochemistry staining in the tumor part (T) (vs. the normal part (N)) of UTUC patient samples are shown by H-score measurement. The error bars represented the standard deviation (SD). Student’s *t* test was used to compare two groups of independent samples. **e** A representative case of immunohistochemistry staining of UTUC patient samples using antibodies against METTL4, 6mA, and HIF-1α between normal and tumor tissues. **f** Co-expression of METTL4 and 6mA predicted a poor prognosis of UTUC patients in either overall survival or disease-free survival by Kaplan-Meier analysis. Subgroup analysis of overall survival and disease-free survival of UTUC cases according to the expression profile of METTL4 low/6mA low (Group 1), METTL4 high/6mA high (Group 2), and others (Group 3) in tumors. *P* values of the comparison between each group are shown

To further test the essential role of the enzymatic activity of METTL4 in mediating EMT, we used a prime-editing CRISPR-Cas9 approach [39] to mutate the enzymatic site (DPPW changed to APAW) of endogenous METTL4 in BFTC909 and FADU cells (Additional file 1: Fig. S3a) and test its effects on 6mA modification and induction of hypoxia-induced gene expression and EMT phenotypes. The results showed that indeed the 6mA levels in the mutant cell lines could not be induced by hypoxia (Fig. 3a; Additional file 1: Fig. S3b). The induction of hypoxia-induced gene expression (lncRNAs and protein-coding genes from RNA-seq datasets analysis) and EMT phenotypes (EMT marker regulation and in vitro migration/invasion activity) was mitigated in METTL4 mutant cell lines (Fig. 3a, b; Additional file 1: Fig. S3b-d), supporting the role of METTL4 enzymatic activity in hypoxia-induced 6mA levels, gene expression, and EMT phenotypes. All these results confirm that the increased 6mA levels and EMT phenotypes induced by METTL4 under hypoxia is dependent on the enzymatic activity of METTL4.

Since METTL4 could mediate 6mA depositions of mitochondria DNAs [11], we generated a nuclear translocation-defective mutant of METTL4 through mutation of its nuclear localization signal (a.a. 132-136, KKRKR changed to AAAAA). The inability of this mutant to translocate into nucleus was confirmed using immunofluorescence staining (Additional file 1: Fig. S3e). Overexpression of this nuclear localization-defective METTL4 mutant could not induce 6mA levels or EMT (by UPLC-ESI-MS/MS, Western blot analysis or immunofluorescence staining) (Additional file 1: Fig. S3f, g) and was unable to increase the in vitro migration/invasion activity (Additional file 1: Fig. S3h). However, this mutant still retained the in vitro 6mA methyltransferase activity (Additional file 1: Fig. S3i). Other control experiments showed that hypoxia induced the mitochondria 6mA levels (Additional file 1: Fig. S3j) and the nuclear localization-defective METTL4 mutant was still able to mediate 6mA modifications of mitochondria DNAs (Additional file 1: Fig. S3k). Finally, to discern whether the nuclear transport mechanism plays a role in the nuclear translocation of METTL4, we screened for the putative nuclear transporter of METTL4 and the results showed that *KPNA7* was induced by hypoxia (Additional file 1: Fig. S3l). Knockdown of *KPNA7* abolished the nuclear translocation of METTL4 by nuclear fractionation assays (Additional file 1: Fig. S3m, n), indicating that hypoxia-induced KPNA7 played a role in the nuclear translocation mechanism of METTL4. This result was confirmed by immunofluorescence staining (Additional file 1: Fig. S3o). Taken together, these results indicate that an increase in nuclear 6mA levels and EMT phenotypes mediated by METTL4 requires its enzymatic activity which needs to be carried out inside the nucleus.

### Co-expression of METTL4 and 6mA is a prognostic marker for upper tract urothelial cancer patients

To further examine the clinical implication of METTL4 and 6mA, we measured the 6mA levels in upper tract urothelial cancer (UTUC) patient samples since HIF-1α contributes to tumor progression in UTUC patients [40]. There was a significant increase in the UTUC but not in bladder carcinoma samples (Fig. 3c). Immunohistochemistry (IHC) staining of METTL4, 6mA, and HIF-1α showed that the levels of METTL4, 6mA, and HIF-1α were increased in the tumor part, but not in the normal part, of UTUC samples (Fig. 3d, and a representative case was shown in Fig. 3e). In addition, there was a significant correlation between the levels of METTL4 and 6mA (Additional file 2: Table S1). Univariate and multivariate analysis of overall survival and disease-free survival of UTUC patients showed a worse prognosis of patients with higher expression levels of METTL4 and 6mA (Additional file 2: Tables S2 and S3). Kaplan-Meier analysis also showed a significantly worse prognosis of UTUC patients with high METTL4/6mA co-expression compared to the low METTL4/6mA patient group (Fig. 3f). All the above results indicate the prognostic value of high METTL4/6mA co-expression in UTUC patients’ survival.

### METTL4 regulates hypoxia-induced programs that contribute to metastasis

**Fig. 4:**
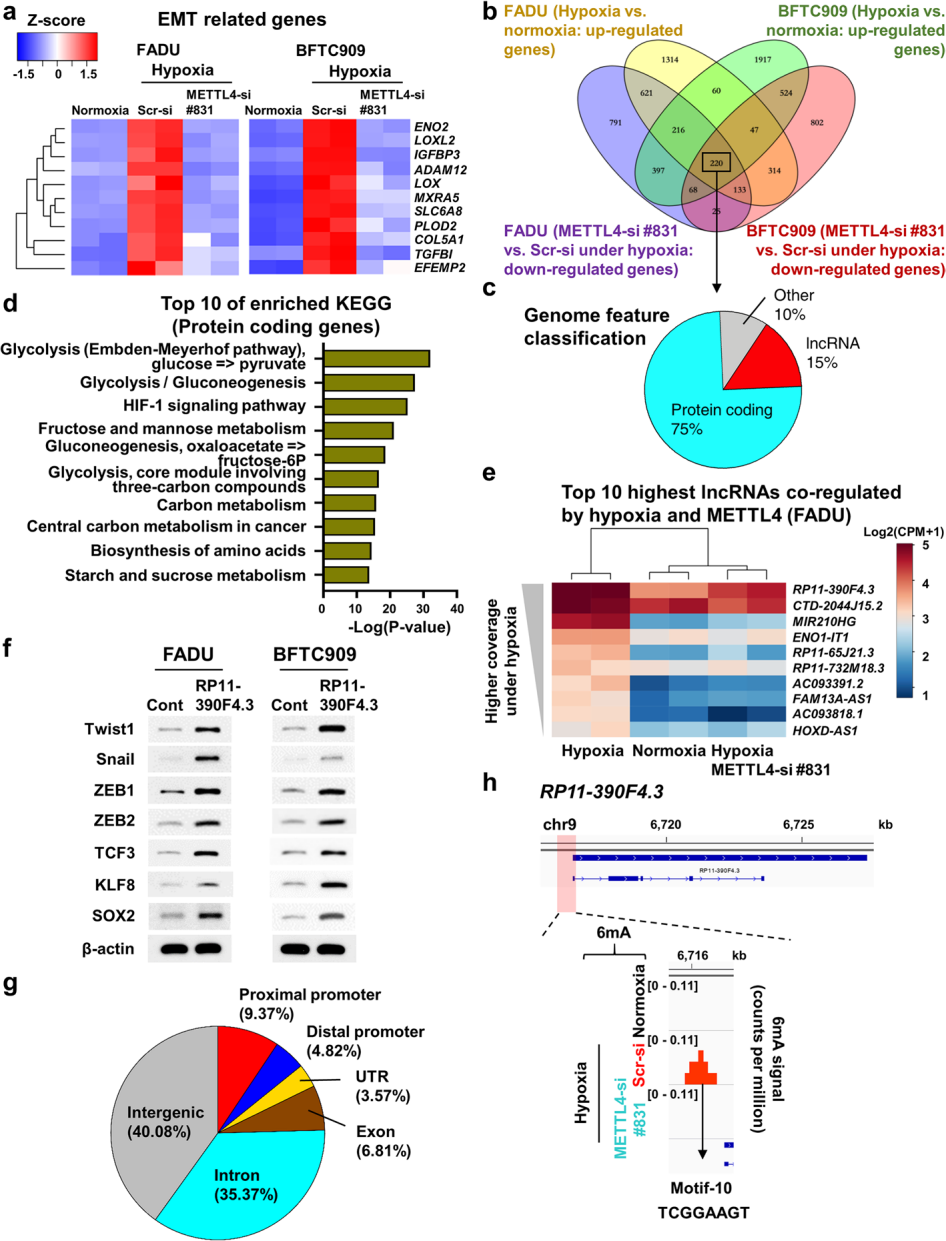
Analysis of RNA-seq and 6mA-ChIP-exo-seq datasets shows hypoxia/METTL4 co-regulated genes and 6mA signals-regulated genes. **a** The expression heatmap of EMT-related genes in FADU and BFTC909 cells, whose expression increased under hypoxia and decreased in the hypoxic status undergoing *METTL4* knockdown. **b** Venn diagram shows the overlapping set of genes (*n*=220) co-regulated by hypoxia and METTL4 through overlapping of upregulated genes under hypoxia and downregulated genes in the hypoxic status undergoing METTL4 knockdown in both FADU and BFTC909 cells. **c** Pie chart shows the different percentage of hypoxia and METTL4 co-regulated genes according to the classification of gene feature (protein coding, 75%; lncRNA, 15%; others, 10%). **d** KEGG analysis shows the top 10 enriched pathways in the class of protein-coding genes co-regulated by hypoxia and METTL4. **e** Heatmap shows the expression of top ten lncRNAs co-regulated by hypoxia and METTL4 using RNA-seq datasets from FADU cells. **f** Western blot analysis shows that overexpression of lncRNA *RP11-390F4.3* activated a set of EMT transcriptional regulators. **g** Pie chart shows the annotation of hypoxia-induced/METTL4 dependent gain-of-6mA regions located in different genomic regions. **h** LncRNA *RP11-390F4.3* was used as an example of hypoxia-induced/METTL4-dependent 6mA regulated gene that contained hypoxia-induced/METTL4-dependent 6mA signals on its promoter region. Different 6mA motifs were calculated by HOMER. Only motif-10 was indicated on the lncRNA *RP11-390F4.3* promoter. A magnified window around the motif-10 area from 3 gene tracks is shown. *Y*-axis denotes the scale of the number of reads

Since METTL4 is essential for hypoxia-induced tumor progression, we analyzed the RNA sequencing (RNA-seq) results by Gene Set Enrichment Analysis (GSEA) from two cell lines treated with hypoxia vs. normoxia and hypoxic status undergoing *METTL4* knockdown vs. hypoxia control knockdown (cut-off by false discovery rate *q*-value < 0.05) (GSE171115) (Additional file 1: Fig. S4a, b). This analysis identified gene sets co-regulated by hypoxia and METTL4, which contain enriched hypoxia-related and the epithelial-mesenchymal transition programs among the major pathways (Additional file 1: Fig. S4a, b). Heatmap of gene expression showed that the EMT-related genes were consistently upregulated by METTL4 under hypoxia in FADU and BFTC909 cells (Fig. 4a). For further analysis of the genes co-regulated by hypoxia and METTL4 from two different cell lines, we performed differentially expressed (DE) gene analysis and considered 1.3 positive/negative fold change with *p*-value < 0.05 as significant differentially expressed genes (Additional file 1: Fig. S4c). After the analysis of various conditions across both cell lines (FADU and BFTC909), the results showed that 220 genes were both induced under hypoxia and regulated by METTL4 (Fig. 4b). Among these genes, 75% were protein-coding genes and 15% belonged to long noncoding RNAs (lncRNAs) (Fig. 4c). KEGG analysis of protein-coding genes showed that HIF-1 signaling pathway was inside the list (Fig. 4d). In contrast, heatmap of expression of lncRNAs revealed that lncRNA *RP11-390F4.3* was located on the top of the list of lncRNAs co-regulated by hypoxia and METTL4 (Fig. 4e). Our previous results showed that lncRNA *RP11-390F4.3* was activated by hypoxia/HIF-1α and overexpression of lncRNA *RP11-390F4.3* induced EMT and increased in vitro migration/invasion and in vivo metastatic activity [41]. In addition, overexpression of lncRNA *RP11-390F4.3* induced the activation of four “core” EMT regulators (Snail, Twist1, ZEB1, and ZEB2) and other EMT regulators (TCF3, KLF8, and SOX2) (Fig. 4f; Additional file 1: Fig. S4d) [41, 42], which was the same set of EMT regulators activated by METTL4 overexpression (Fig. 2c; Additional file 1: Fig. S2g). Due to this finding, we tested whether METTL4 and lncRNA *RP11-390F4.3* had signaling connection. Real-time PCR analysis showed that induction of lncRNA *RP11-390F4.3* by hypoxia was mitigated by knockdown of *METTL4*, indicating that lncRNA *RP11-390F4.3* was regulated by METTL4 (Additional file 1: Fig. S4e). We also analyzed other lncRNAs that were activated under hypoxia [43], and the heatmap analysis showed that most of the reported hypoxia-activated lncRNAs were upregulated, but only some of them were regulated by METTL4 (Additional file 1: Fig. S4f). The above results identify the set of protein-coding and lncRNA genes that are co-regulated by hypoxia and METTL4.

We also analyzed the repressed genes that were co-regulated by hypoxia and METTL4. The results showed that certain categories of genes (mitochondria process, chromatin stability, and RNA metabolism) were regulated (Additional file 1: Fig. S4g), indicating that these genes may be repressed by METTL4 under hypoxia.

### Whole genome 6mA profiling identifies the groups of 6mA-activated genes that play a role in hypoxia-induced metastasis

Next, we applied the 6mA chromatin immunoprecipitation-exonuclease digestion method [11] followed by sequencing (ChIP-exo-seq) to identify differential 6mA signals from hypoxia vs. normoxia as well as from hypoxic status undergoing *METTL4*-knockdown vs. hypoxia control knockdown (GSE171116) (Additional file 1: Fig. S4h). The 6mA signals were mostly located on the promoter region around TSS and gene body (Additional file 1: Fig. S4h). Two different comparisons using different datasets were performed to generate 6mA signal regions regulated by METTL4 under hypoxia (8268 from comparison 1 and 76,695 from comparison 2) (Additional file 1: Fig. S4i) (see “Methods”). In addition, a whole genome amplification (WGA) method to amplify DNAs pulled down by anti-6mA antibodies under hypoxic condition followed by sequencing was used to correct the background noise underlying the 6mA signals pulled down by anti-6mA antibodies (Additional file 1: Fig. S4j). After subtracting the WGA signals from the hypoxia-enriched 6mA signals, the remaining 6mA signals were still located on the promoter region around the TSS and gene bodies (Additional file 1: Fig. S4h). By overlapping the 6mA signals subtracted by WGA control and the 6mA signals from comparisons 1 and 2, Venn diagram showed that the 6mA signals were further narrowed down to 3673 regions defined as hypoxia-induced/METTL4-dependent gain-of-6mA regions (Additional file 1: Fig. S4j). Among the 3673 gain-of-6mA regions, different percentage of 6mA signal regions were present in different genomic areas with the following distribution: intergenic (40.08%), intron (35.37%), proximal promoter (9.37%), exon (6.81%), distal promoter (4.82%), or UTR (3.57%) region (Fig. 4g). The hypoxia-induced/METTL4-dependent gain-of-6mA regions were correlated with upregulated genes as well as downregulated genes in a similar magnitude (Additional file 1: Fig. S4k). By focusing on METTL4-dependent hypoxia-activated genes containing hypoxia-induced/METTL4-dependent gain-of-6mA regions in FADU cells, a total of 263 upregulated genes were identified (Additional file 1: Fig. S4l). KEGG analysis of these 263 genes showed the category of genes (Additional file 1: Fig. S4l). We also analyzed this group of genes and overlapped the list with the genes bound by HIF-1α using HIF-1α ChIP-seq datasets from FADU cells (GSE171116) (Additional file 1: Fig. S4m). One of the overlapping gene groups belonged to the HIF-1α target genes whose genomic regions were bound by HIF-1α (54 genes) (Additional file 1: Fig. S4m). It is noticeable that another set of HIF-1α target genes (209 genes) were regulated by 6mA signals but did not require direct assembly of HIF-1α to their genomic loci (Additional file 1: Fig. S4m). It is interesting that these target genes represented various pathways (i.e., angiogenesis, stemness, cancer metabolism) that could be mediated by hypoxia (Additional file 1: Fig. S4m), supporting that hypoxia-mediated phenotypes could be regulated by 6mA signals. KEGG analyses of all the METTL4-dependent hypoxia-activated genes were shown (Additional file 1: Fig. S4m). The 6mA consensus motifs located in the upregulated genes calculated by HOMER are shown (Additional file 1: Fig. S4n). We observed that sequences homologous to motif-10 could be identified on the promoter of lncRNA *RP11-390F4.3* (Fig. 4h; gene track analysis was included), consistent with its regulation by METTL4 through 6mA deposition on its promoter. Another hypoxia/METTL4-regulated gene that contained 6mA signals in its intron 1 region (sequences homologous to motif-6) was a co-activator, ZMIZ1, that may play a role in enhancing HIF-1α-induced gene transcription (see results below) (Additional file 1: Fig. S4o). Bioinformatics analysis also showed that different consensus sequence motifs were located in different genomic locations of two representative HIF-1α target genes that were regulated by 6mA without direct assembly of HIF-1α to their loci, including *CTNNA1* (containing motif-9) and *PIK3CA* (containing motif-7) (Additional file 1: Fig. S4o). Gene track analyses were shown for these three genes (Additional file 1: Fig. S4o). The profiling results above show that most target genes co-regulated by hypoxia and METTL4 through 6mA deposition play a role in hypoxia-mediated phenotypes.

### U2 snRNA and its m^6^Am modification are not involved in hypoxia/METTL4-mediated EMT

Since METTL4 was shown to also increase the m^6^Am methylation of *U2 snRNA* (Additional file 1: Fig. S1q-s) [35, 36], we tested whether it was able to mediate m^6^Am modification of lncRNA *RP11-390F4.3*. The results showed that METTL4 was unable to mediate the m^6^Am modification of lncRNA *RP11-390F4.3* from various assays (Additional file 1: Fig. S1q-s). We further tested the role of *U2 snRNA* in mediating differential RNA splicing, METTL4-mediated EMT, and in vitro migration/invasion activity. We profiled the differential splicing events induced by hypoxia vs. normoxia and *METTL4* knockdown under hypoxia vs. hypoxia control knockdown from two cell lines (Additional file 1: Fig. S4p, left upper panel). The upregulated splicing events induced by hypoxia were overlapped with downregulated splicing events in the condition of *METTL4* knockdown under hypoxia (Additional file 1: Fig. S4p, left upper panel). The differential splicing events were categorized (Additional file 1: Fig. S4p, left lower panel). The 154 differential splicing events in BFTC909 cells and 175 differential splicing events in FADU cells that were co-regulated by hypoxia and METTL4 are shown (Additional file 1: Fig. S4p, left lower panel). KEGG analysis showed that these differential splicing events were not related to EMT (Additional file 1: Fig. S4p, right upper panel). Further overlapping of these events between two different cell lines showed that 5 genes containing skipped exon (SE) or alternative 3′ splice site (A3SS) events (Additional file 1: Fig. S4p, right lower panel). We further characterized *CEP192* and *ANXA11* among these 5 genes by their differential splicing events (the patterns of differential splicing are shown; Additional file 1: Fig. S4q, upper part). Indeed, the differential splicing of these two RNAs were regulated by hypoxia and returned to the original splicing pattern under METTL4 knockdown (Additional file 1: Fig. S4q, left lower panel). However, the differential splicing of RNAs (*UBE3C*, *KLHDC2*) that were reported to be regulated by *U2 snRNA* were not regulated in our cell lines tested [35], which may be due to the different cellular background (Additional file 1: Fig. S4q, right lower panel).

We further tested the ability of *U2 snRNA* to regulate in vitro migration and invasion activity of tumor cells. Overexpression of *U2 snRNA* did not increase the mRNA levels of *RP11-390F4.3*, the in vitro migration/invasion activity, or the protein levels of various EMT regulators in two cell lines (Additional file 1: Fig. S4r). These phenotypes also did not occur in the condition of *U2 snRNA* knockdown under hypoxia (Additional file 1: Fig. S4s). We subsequently characterized the specificity of m^6^Am methylation of *U2 snRNA* in regulating the differential splicing of these RNAs. The ability of *U2 snRNA* to regulate differential splicing was tested in cells overexpressing METTL4 under *U2 snRNA* knockdown followed by reconstitution of wild type *U2 snRNA* or A30-mutated *U2 snRNA*. The m^6^Am methylation of these reconstituted *U2 snRNAs* (wild type vs. point mutant) is shown (Additional file 1: Fig. S4t, left panel). Differential splicing assays showed that wild type *U2 snRNA*, but not the A30-mutated *U2 snRNA*, was able to rescue the differential splicing of *CEP192* and *ANXA11* (Additional file 1: Fig. S4t, right panel), supporting the role of m^6^Am modification of *U2 snRNA* in carrying out differential splicing of specific genes. Further testing of the m^6^Am methylation effects of *U2 snRNA* showed that knockdown of *U2 snRNA* under METTL4 overexpression followed by reconstitution with either wild type *U2 snRNA* or A30-mutanted *U2 snRN*A did not change the protein levels of various EMT regulators, EMT markers, or the in vitro migration and invasion activity of two different cell lines (Additional file 1: Fig. S4u, v). These results supported that the role of METTL4 in regulating hypoxia-induced EMT and metastasis was not through the m^6^Am modification of *U2 snRNA*.

### LncRNA RP11-390F4.3 induces the epithelial-mesenchymal transition by activating multiple EMT regulators via lncRNA-chromatin interaction

**Fig. 5:**
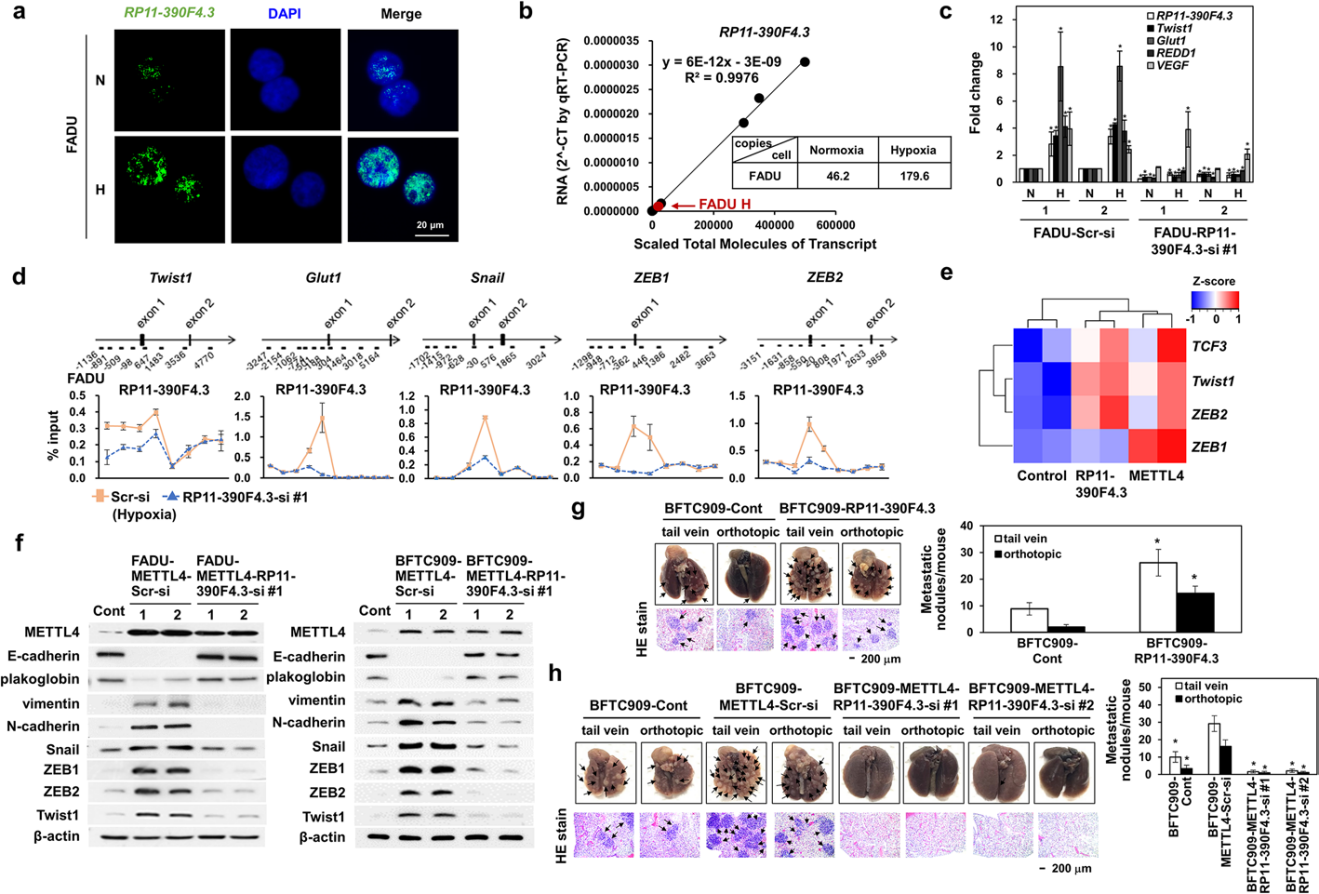
Characterizations of lncRNA *RP11-390F4.3* using different in vitro and in vivo metastatic assays. **a** Immunofluorescence staining shows the nuclear localization of lncRNA *RP11-390F4.3.* Cell nuclei were stained by DAPI. **b** Measurement of the copy number of lncRNA *RP11-390F4.3* in FADU cells (normoxia vs. hypoxia). Titration standard curve was used for measurement of the copy number of lncRNA *RP11-390F4.3* per 500,000 cells. The red point represents the qRT-PCR value from a standard sample of 500,000 FADU cells under hypoxia. **c** Knockdown of lncRNA *RP11-390F4.3* significantly decreased the induction of various HIF-1α target genes using qRT-PCR analysis. Knockdown using the scrambled control siRNA was used as a control. N, normoxia; H, hypoxia. The asterisk (*) indicated statistical significance (*P*<0.05) between experimental and control clones (*n*=3). **d** qChIRP assays show the significantly decreased lncRNA *RP11-390F4.3* binding to the promoter regions of EMT regulators and *Glut1* gene in hypoxic FADU cells under knockdown of lncRNA *RP11-390F4.3* vs. the hypoxic control knockdown cells. **e** Heatmap analysis of the EMT transcription regulator genes induced by lncRNA *RP11-390F4.3* or METTL4 overexpression using RNA-seq datasets. **f** Western blot analysis shows that knockdown of lncRNA *RP11-390F4.3* abolished the EMT phenotypes induced by METTL4 overexpression in FADU and BFTC909 cells. **g** Overexpression of lncRNA *RP11-390F4.3* in BFTC909 cells significantly increased metastatic lung nodules in mice after injection of these cells into mice. Representative gross anatomy and histology are shown on the left, and measurement of metastatic lung nodules is shown on the right. BFTC control-transfected clone was used as a control. **h** Knockdown of lncRNA *RP11-390F4.3* in BFTC909 cells overexpressing METTL4 significantly decreased the metastatic lung nodules in mice. Representative gross anatomy and histology are shown on the left, and measurement of metastatic lung nodules is shown on the right. BFTC control-transfected clone was used as a control. The asterisk (*) indicates statistical significance (*P*<0.05) between experimental and control groups

Since Western blot analysis showed that METTL4 and lncRNA *RP11-390F4.3* overexpression activated the same set of EMT regulators (Fig. 2c, 4f) and lncRNA *RP11-390F4.3* contains 6mA depositions on its promoter, we further characterized lncRNA *RP11-390F4.3.* To identify the localization of lncRNA *RP11-390F4.3*, we performed single-molecule RNA-FISH detection of lncRNA *RP11-390F4.3* in tumor cell lines. The results showed that lncRNA *RP11-390F4.3* was located inside nucleus (Fig. 5a; Additional file 1: Fig. S5a). Since there are three versions of lncRNA *RP11-390F4.3* from the recent annotations of lncRNAs (Ensembl-GRCh37.p13) (Additional file 1: Fig. S5b), we tested which version had the highest activation fold and the result showed that V1 had the highest fold of activation (~4 to 6 fold) inside the nucleus (Additional file 1: Fig. S5b). We further performed RNA-FISH assays to measure the copy number of lncRNA *RP11-390F4.3* using lncRNA *NEAT1* as a positive control. The results showed that the copy number of lncRNA *RP11-390F4.3* was ~46 copies per cell compared to ~1296 copies of *NEAT1* in FADU cells (Fig. 5b; Additional file 1: Fig. S5c). The copy number of lncRNA *RP11-390F4.3* in BFTC909 cells was significantly less (Additional file 1: Fig. S5c). Furthermore, knockdown of lncRNA *RP11.390F4.3* abolished the activation of HIF-1α target genes induced by hypoxia (*Twist1, Glut1,* and *REDD1*) in FADU cells under hypoxia (Fig. 5c; Additional file 1: Fig. S5d). qChIRP (quantitative chromatin isolation by RNA purification) results showed the decreased occupancy levels of lncRNA *RP11-390F4.3* on the promoter of *Twist1*, *Snail*, *ZEB1*, *ZEB2*, and *Glut1* genes (Fig. 5d; Additional file 1: Fig. S5e), indicating lncRNA-chromatin interaction [44]. The specificity of the qChIRP method was confirmed by the retrieval of lncRNA *RP11.390F4.3*, but not *HOTAIR*, by our tiling probes (Additional file 1: Fig. S5f). Control experiments showed that lncRNA *RP11-390F4.3* was not located on the promoters of *VEGF*, *E-cadherin*, and *N-cadherin* (Additional file 1: Fig. S5g). We further confirmed the above results by RNA-seq results from FADU cells overexpressing lncRNA *RP11.390F4.3* or METTL4 (Fig. 5e), supporting the regulation of these genes by lncRNA *RP11-390F4.3*.

To test the tumorigenic ability of lncRNA *RP11-390F4.3*, BFTC909 and FADU cells overexpressing lncRNA *RP11-390F4.3* showed increased tumor volume in xenograft experiments (Additional file 1: Fig. S5h and ref. 41). In contrast, increased tumor volume by METTL4 overexpression was significantly decreased under knockdown of lncRNA *RP11-390F4.3* in two cell lines (Additional file 1: Fig. S5i, j), indicating that the tumorigenic activity induced by METTL4 was mediated by lncRNA *RP11-390F4.3*. To show the essential role of lncRNA *RP11-390F4.3* in METTL4-mediated EMT and in vivo metastatic activity, overexpression of METTL4 followed by knocking down lncRNA *RP11-390F4.3* reversed the EMT induced by METTL4 overexpression in two cell lines (Fig. 5f). In vivo metastatic activity assays also showed that overexpression of lncRNA *RP11-390F4.3* in BFTC909 and FADU cells increased the number of metastatic lung nodules in mice (Fig. 5g and ref. [41]). This increase in the number of metastatic lung nodules was significantly decreased by knockdown of lncRNA *RP11-390F4.3* (Fig. 5h; Additional file 1: Fig. S5k). Finally, high lncRNA *RP11-390F4.3* expression was shown to predict a worse survival of head and neck cancer patients from the TCGA dataset analysis (Additional file 1: Fig. S5l). All the above results support the crucial role of lncRNA *RP11-390F4.3* in the increase in tumor volume and induction of EMT and in vivo metastatic activity mediated by METTL4 in BFTC909 and FADU cells.

### Discovery of a HIF-1α co-activator, ZMIZ1, that is regulated by 6mA and contributes to EMT and in vitro migration/invasion activity

**Fig. 6:**
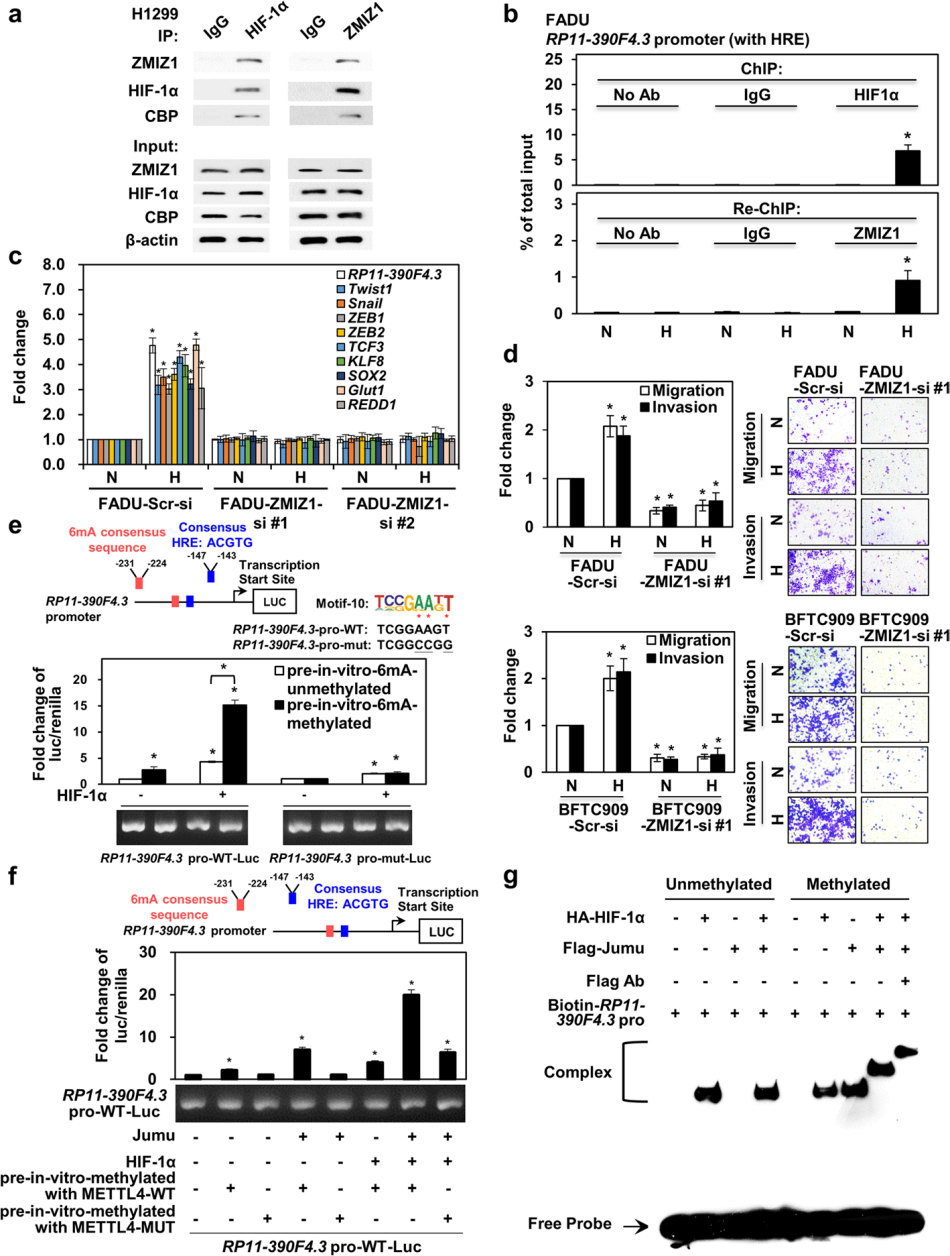
Characterizations of ZMIZ1 and synergistic activation by HIF-1α and Jumu using different in vitro assays. **a** Co-IP assays show the interaction between HIF-1α, ZMIZ1, and CBP. IgG was used as a control. **b** ChIP-re-ChIP assays show that ZMIZ1 could be pulled down after HIF-1α IP. N, normoxia; H, hypoxia. No antibody/normoxia condition was used as a control. **c** Knockdown of *ZMIZ1* decreased the induction of various HIF-1α target genes. Knockdown using the scrambled control siRNA was used as a control. N, normoxia; H, hypoxia. **d** Knockdown of *ZMIZ1* decreased the in vitro migration and invasion activity induced by hypoxia in FADU and BFTC909 cell lines. N, normoxia; H, hypoxia. Normoxic condition was used as a control. **e** Reporter gene assays show that the 6mA site pre-methylated *RP11-390F4.3* promoter-driven reporter construct has higher luciferase activities compared to the unmethylated reporter construct after co-transfection with a HIF-1α expression vector. The positions of the 6mA consensus sequence and the HIF-1α response element are shown in the upper part of the panel. The luciferase/renilla activities of FADU cells co-transfected with reporter construct and pcDNA3 control vector were used as the baseline control. The amounts of plasmids transfected inside cells are shown in agarose gels. **f** Reporter gene assays show that HIF-1α and Jumu (a Drosophila 6mA-binding protein) synergistically activated the lncRNA *RP11-390F4.3* promoter-driven reporter construct in which its 6mA site on the promoter was pre-methylated. The upper part of the panel shows the positions of the 6mA consensus sequence and the consensus HRE on the *RP11-390F4.3* promoter. The controls were the same as in **e**. **g** DNA EMSA assays show the cooperative binding between HIF-1α and Jumu when the oligonucleotides containing the 6mA consensus sequence were in vitro methylated. The positions of the free probe and of the protein complexes are indicated on the left. The asterisk (*) indicates statistical significance (*P*<0.05) between experimental and control groups

Since 6mA signals could be discovered in the intron 1 region of a co-activator, *ZMIZ1* (Additional file 1: Fig. S4o), we confirmed that *ZMIZ1* expression was activated by hypoxia and METTL4 overexpression by real-time PCR analysis (Additional file 1: Fig. S6a). Co-immunoprecipitation assays showed that HIF-1α interacted with ZMIZ1 and CBP (Fig. 6a). ChIP followed by Re-ChIP assays showed that indeed ZMIZ1 could be pulled down by anti-ZMIZ1 antibodies from the initial ChIP using anti-HIF-1α antibodies that both occupied the lncRNA *RP11-390F4.3* promoter in two different cell lines (Fig. 6b; Additional file 1: Fig. S6b). Knockdown of *ZMIZ1* significantly decreased the induction of various HIF-1α target genes in FADU and BFTC909 cells by real-time PCR analyses (Fig. 6c; Additional file 1: Fig. S6c), indicating the essential role of ZMIZ1 as a co-activator of HIF-1α. Western blot analysis also showed that knockdown of *ZMIZ1* abolished hypoxia-induced EMT in two cell lines (Additional file 1: Fig. S6d). Finally, knockdown of *ZMIZ1* decreased the in vitro migration and invasion activity induced by hypoxia in two cell lines (Fig. 6d; Additional file 1: Fig. S6e). The above results support the essential role of ZMIZ1 in hypoxia-induced gene expression and phenotypes.

### Regulation of lncRNA RP11-390F4.3, ZMIZI, and other HIF-1α target genes by 6mAs

Since the putative 6mA sites have been localized to the proximal promoter of lncRNA *RP11-390F4.3*, we used the in vitro DNA methylation assays to measure the 6mA levels by incubating the purified METTL4 with the oligonucleotides containing the 6mA site (TCGGAAGT, −231 to −224 bp upstream of TSS) on the lncRNA *RP11-390F4.3* promoter. The oligonucleotides containing the mutated 6mA site (TCGGAAGT to TCGGCCGG) were also tested. The 6mA site on the promoter of lncRNA *RP11-390F4.3* was confirmed by methylation DNA immunoprecipitation (MeDIP) assays (Additional file 1: Fig. S6f, left panel). In vitro DNA methylation assays followed by UPLC-ESI-MS/MS analysis showed that only the wild type 6mA oligonucleotides could be methylated at the 6mA site by METTL4, but not the mutated oligonucleotides or the reaction not incubating with SAM (Additional file 1: Fig. S6f, right panel). We further tested whether the lncRNA *RP11-390F4.3* promoter-driven reporter construct containing this 6mA site (TCGGAAGT, −231 to −224 bp upstream of TSS) could be activated by METTL4. This motif was chosen due to its proximity to the HRE (hypoxia response element: ACGTG, −147 to −143 bp upstream of TSS). Indeed, this reporter construct was activated by the wild-type, but not the mutant, METTL4 in a dose-dependent manner (Additional file 1: Fig. S6g). We further mutated the putative 6mA site (TCGGAAGT to TCGGCCGG) and generated a mutant reporter construct. Activation by METTL4 was only shown in the wild type lncRNA *RP11-390F4.3* promoter-driven reporter construct, but not in the 6mA site-mutated lncRNA *RP11-390F4.3* promoter-driven reporter construct (Additional file 1: Fig. S6g). Co-transfection assays showed that HIF-1α and METTL4 synergistically activated the wild type, but not the mutant, lncRNA *RP11-390F4.3* promoter-driven reporter construct (Additional file 1: Fig. S6g). Further experiments to test the role of 6mA presence on the promoter of *RP11-390F4.3* was performed using in vitro methylation experiments to pre-methylate the promoter of a *RP11-390F4.3*-driven wild type reporter construct (vs. a 6mA site-mutated reporter construct). The methylation status was confirmed using UPLC-ESI-MS/MS assays (Additional file 1: Fig. S6h). Transient transfection assays with the reporter constructs (unmethylated vs. methylated; wild type vs. mutant) and a HIF-1α expression vector showed that the methylated reporter construct had a higher luciferase activity compared to the unmethylated version (Fig. 6e). Co-transfection of HIF-1α further activated the pre-methylated reporter construct, whereas the 6mA site-mutated reporter construct had low luciferase activity and was only mildly activated by HIF-1α (Fig. 6e), supporting the role of 6mAs on the target gene promoter in promoting gene expression.

To further delineate the molecular mechanism of 6mA-mediated gene activation, we tested a putative 6mA-binding protein from *Drosophila*, Jumu, for its functional interaction with HIF-1α [45]. Reporter gene assays using the lncRNA *RP11-390F4.3* promoter-driven reporter construct containing the 6mA site followed by co-transfections of HIF-1α, METTL4, and Jumu expression vectors showed a synergistic activation of the lncRNA *RP11-390F4.3* promoter-driven reporter construct, indicating that methylation of the 6mA site followed by binding by Jumu facilitated the synergistic interaction between HIF-1α and Jumu (Additional file 1: Fig. S6i). This synergistic activation was also observed using an in vitro pre-methylated *RP11-390F4.3* promoter-driven reporter construct together with HIF-1α and Jumu expression vectors (Fig. 6f), further confirming the role of 6mA and its binding protein in promoting HIF-1α target gene expression. To test the functional cooperation between HIF-1α and Jumu, DNA electrophoretic mobility shift assays (EMSAs) were performed. Incubating the lncRNA *RP11-390F4.3* promoter DNA fragment without METTL4 methylation with purified Jumu showed that there was no binding of Jumu to the fragment (Fig. 6g). Binding of Jumu to the fragment appeared after the DNA fragment was in vitro methylated (Fig. 6g). Further EMSAs showed that there was a cooperative binding between HIF-1α and Jumu when the DNA fragment was in vitro 6mA-methylated by METTL4 (Fig. 6g). The above results indicate that a 6mA-binding protein, through binding to the methylated 6mA site, functionally interacts with HIF-1α to achieve synergistic activation of a promoter.

We further mutated the 6mA site on the *RP11-390F4.3* promoter in FADU and BFTC909 cells using a prime-cutting CRISPR-Cas9 approach [39]. We generated clones containing heterozygous mutation of the 6mA site (Additional file 1: Fig. S6j). Functional assays of these clones showed that mutation of one copy of the 6mA site on the promoter of lncRNA *RP11-390F4.3* gene already reduced the activation of lncRNA *RP11-390F4.3* down to ~30% of the wild type *RP11-390F4.3* expression induced by hypoxia (Additional file 1: Fig. S6k). The regulation of EMT markers and regulators mediated by hypoxia was abolished in the heterozygous mutated clones (Additional file 1: Fig. S6l, m). The activation of in vitro migration and invasion activity induced by hypoxia was also abolished in these clones (Additional file 1: Fig. S6n). We further generated a FADU clone that contained homozygous mutation of the 6mA site on the LncRNA *RP11-390F4.3* promoter (Additional file 1: Fig. S6j). In addition to the abolishment of EMT and in vitro migration/invasion activity induced by hypoxia in this clone, the activation of lncRNA *RP11-390F4.3* by hypoxia was totally abolished (Additional file 1: Fig. S6o-q), indicating the essential role of this 6mA site in the process of hypoxia-induced gene activation.

The 6mA modification on the intron 1 region of *ZMIZ1* induced by hypoxia was confirmed by MeDIP assays showing the 6mA site located on the intron 1 (Additional file 1: Fig. S6r). This 6mA site was further tested by transfecting the reporter constructs containing part of the *ZMIZ1* intron 1 (containing the consensus 6mA site vs. the deleted or mutated 6mA site version). The results showed that the reporter construct containing the wild type 6mA site could be activated by co-expressing METTL4, but not the reporter construct containing the deleted or mutated 6mA site (Additional file 1: Fig. S6s). In vitro DNA methylation assays showed that the methylation occurred on the intron 1 region of *ZMIZ1* gene containing the wild type 6mA site, but not the deleted or mutated 6mA site version or not incubating with SAM (Additional file 1: Fig. S6t). Using the in vitro pre-methylated *ZMIZ1* reporter construct, the reporter gene assay confirmed that the presence of 6mAs increased the reporter gene activity vs. the unmethylated reporter construct (Additional file 1: Fig. S6t). The *ZMIZ1* reporter gene construct containing the wild type 6mA site was transfected into the METTL4 wild type or enzymatically inactive mutant FADU or BFTC909 cells. The results showed that the METTL4 mutant cells could not activate the reporter construct under hypoxia, supporting that 6mA methylation by METTL4 was required to activate the *ZMIZ1* reporter gene construct (Additional file 1: Fig. S6u). The correlation between expressions of 6mA and lncRNA *RP11-390F4.3*/*ZMIZ1* or between expressions of METTL4 and lncRNA *RP11-390F4.3*/*ZMIZ1* was shown by testing the clinical samples (Additional file 2: Tables S4 and S5).

Since the 6mA signals were discovered on the introns of other target genes including *CTNNA1* and *PIK3CA* (Additional file 1: Fig. S4o), we also confirmed the activation of these genes by hypoxia or METTL4 overexpression using real-time PCR assays (Additional file 1: Fig. S6v). MeDIP assays showed the presence of 6mA sites on the specific intron for *CTNNA1* or *PIK3CA* gene, respectively (Additional file 1: Fig. S6w). In vitro DNA methylation assays also showed the increased 6mA levels through METTL4-mediated methylation of the intronic 6mA site on both genes (Additional file 1: Fig. S6w).

To test the ability of 6mA ChIP-exo-seq data to predict bona fide 6mA sites, we further confirmed the activation of 5 genes (*DAAM1, LEPREL1, XPNEPEP1, TBC1D23,* and *TNIP1*) by hypoxia or METTL4 overexpression by qRT-PCR in two different cell lines (Additional file 1: Fig. S6x). The specific 6mA sites were localized from gene track analysis of each gene (Additional file 1: Fig. S6y). MeDIP assays confirmed the presence of the specific 6mA sites on the promoter or intronic regions of each gene in FADU and BFTC909 cells (Additional file 1: Fig. S6z).

### Overlapping gene sets induced by METTL4/6mA, lncRNA RP11-390F4.3, and HIF-1α fall within the metastasis-inducing pathways

**Fig. 7:**
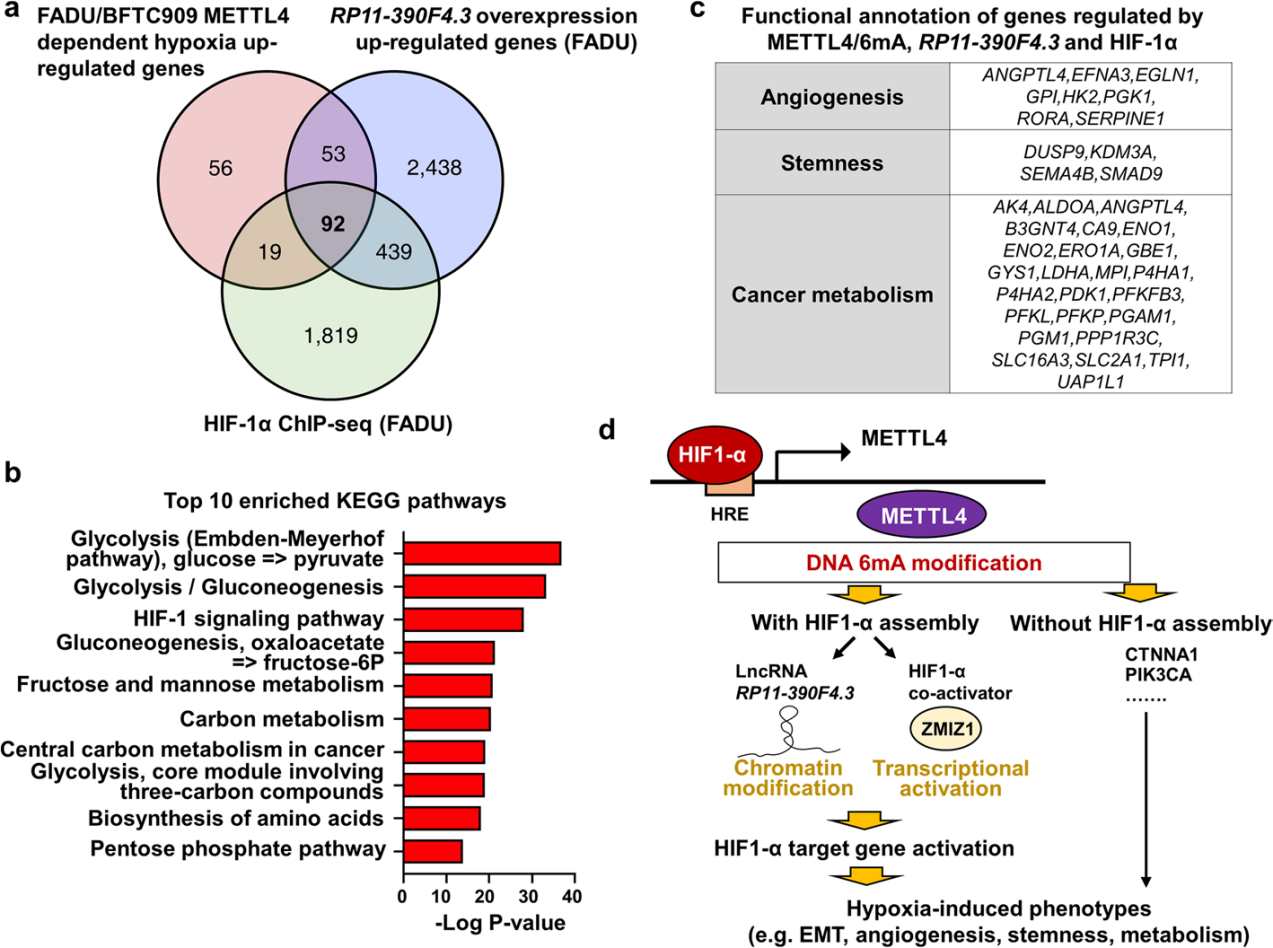
Analysis of overlapping datasets regulated by METTL4/6mA, lncRNA *RP11-390F4.3*, and HIF-1α, and a summary model. **a** Venn diagram shows the number of genes that was co-regulated by METTL4/6mA, lncRNA *RP11-390F4.3*, and HIF-1α. **b** KEGG analysis of the 92 co-regulated genes, in which glycolysis and HIF-1 signaling pathways are on the top of the list. **c** Categorization of the 92 genes shows that they mainly belonged to the main hypoxia-induced phenotypes (angiogenesis, stemness, cancer metabolism). **d** A model to summarize the results and the mechanisms from this report

From the 220 genes that were co-regulated by hypoxia and METTL4 (Fig. 4b), we wanted to examine the contribution of lncRNA *RP11-390F4.3* to the regulation of these genes. After overlapping these 220 genes with the genes induced by lncRNA *RP11-390F4.3* overexpression, a list of 145 genes was generated (Fig. 7a). Through further overlapping with a HIF-1α ChIP-seq dataset, 92 genes were bound by HIF-1α, whereas 53 genes were without HIF-1α assembly to their genomic loci (Fig. 7a). KEGG analysis showed that glycolysis and HIF-1α signaling pathways were listed on the top of the list of 92 genes (Fig. 7b), suggesting that lncRNA *RP11-390F4.3* collaborated with HIF-1α to regulate the majority of genes co-regulated by hypoxia and METTL4 (Fig. 7a). In addition, functional annotation of the list of overlapping gene set showed that these genes still belonged to the three major phenotypes (angiogenesis, stemness, cancer metabolism) induced by hypoxia (Fig. 7c). These results indicate that a majority of hypoxia/6mA-regulated genes were regulated by lncRNA *RP11-390F4.3* for their expression, supporting the crucial role of the HIF-1α-METTL4-lncRNA *RP11-390F4.3* axis in regulating the expression of certain HIF-1α target genes and recapitulating hypoxia-induced phenotypes.

## Discussion

Due to the low abundance of DNA 6mA modifications in eukaryotes, the biological roles of 6mA in mammalian cells have long been elusive. As various stress conditions have been shown to induce 6mA levels in *C. elegans*, mouse brain, mouse trophoblast stem cells, and mammalian mitochondria [11–14], here we show that nuclear 6mA levels are induced by hypoxia in mammalian tumor cells, regulating hypoxia-induced gene expression and promoting cancer metastasis. Since hypoxia is a physiological stress condition and a microenvironmental factor in inducing tumor metastasis [21, 23, 38], all the results described above are consistent with the picture that 6mA levels are induced under different stress conditions. In this report, METTL4 is demonstrated as a nuclear DNA 6mA methyltransferase [11, 19, 20] and plays a crucial role in hypoxia-induced EMT and metastasis. Co-expression of 6mAs and METTL4 predicts a poor survival of UTUC patients. Due to the regulation of the same set of “core” EMT regulators by METTL4 and lncRNA *RP11-390F4.3* (Fig. 2c, 4f) [41], the HIF-1α-METTL4-*RP11-390F4.3* axis plays a crucial role in hypoxia-induced tumor progression. The scaffolding of a putative chromatin-modifying complex by lncRNA *RP11-390F4.3* needs to be further characterized. METTL4 may serve as a target for future therapy against specific types of cancer (e.g., UTUC, head and neck cancer).

Hypoxia-regulated gene expression has been shown to be mediated through both transcriptional and epigenetic mechanisms that contribute to hypoxia-induced tumor metastasis [21–23]. In this report, we show that the epigenetic regulation (via lncRNA *RP11-390F4.3*) and transcriptional regulation (via an unidentified HIF-1α co-activator, ZMIZ1) are crucial in hypoxia-induced metastasis. Under this scenario, 6mA depositions on the promoters of these two genes as well as in the promoters of other HIF-1α target genes (e.g., *CTNNA1*, *PIK3CA*) play a crucial role in hypoxia-induced metastasis. Therefore, targeting METTL4 that regulates 6mA depositions on the regulatory regions of these metastasis-inducing genes will be a reasonable strategy of controlling hypoxia-induced metastasis.

To determine the molecular mechanism of 6mA sites that mediate gene activation, we show that Jumu, a *Drosophila* 6mA-binding protein [45], together with HIF-1α synergistically activates a HIF-1α target gene (i.e., lncRNA *RP11-390F4.3*) containing 6mA sites and HRE on its promoter by reporter gene assays. In addition, cooperative binding between HIF-1α and Jumu has been demonstrated using a promoter fragment of lncRNA *RP11-390F4.3* containing HRE and a 6mA site that is methylated by METTL4. Further characterizations of the mammalian 6mA-binding sites and identification of mammalian 6mA-binding proteins are required to understand the precise roles played by 6mAs to regulate gene expression in mammalian cells.

METTL4 has been shown to demonstrate *U2 snRNA* m^6^Am methyltransferase activity [35, 36]. We further characterized this activity in relation to hypoxia/METTL4-mediated EMT and metastasis. Our results showed that m6Am modification of *U2 snRNA* mediated by METTL4 did not contribute to hypoxia/METTL4-induced EMT and metastasis (Additional file 1: Fig. S4p-v), consistent with the report that *U2 snRNA* is not involved in cellular migration and invasion activity [46]. Therefore, the DNA 6mA methyltransferase activity mediated by METTL4 in this report should be the major activity that induces hypoxia-induced EMT and metastasis.

## Conclusions

These findings demonstrate that hypoxia/METTL4-mediated nuclear 6mA depositions induce tumor metastasis through activating multiple metastasis-inducing genes, identifying METTL4 as a therapeutic target for hypoxia-involved tumors.

## Methods

### Cell culture, oxygen deprivation, and cellular stress conditions

The human head and neck cancer (FADU) and renal pelvis transitional cancer (BFTC909) cell lines were obtained from the Bioresource Collection and Research Center (BCRC, Hsinchu, Taiwan). The human lung cancer (H1299) cell line was obtained from ATCC. The embryonic kidney 293T (HEK293T) cell line was described [38]. KTCC28M, a primary cell line generated from human upper tract urothelial carcinoma cells, was obtained from Dr. See-Tong Pang (Chang Gung Memorial Hospital at Linkou, Taoyuan, Taiwan). Use of KTCC28M cells was approved by the Institutional Review Board of Chang Gung Memorial Hospital (201303642A3C501). Cell lines were cultured in Dulbecco’s modified Eagle’s medium (DMEM) or RPMI medium supplemented with 10% FBS and 1% PS*.* Culture conditions were maintained at 37°C in a humidified incubator containing 5% CO_2_. The cell lines showed negative results for mycoplasma contamination by mycoplasma DNA PCR and ultra-performance liquid chromatography with electrospray ionization tandem mass spectrometry (UPLC-ESI–MS/MS) assays. Oxygen deprivation was carried out by incubating the cells under 1% O_2_, 5% CO_2_, and 94% N_2_ for 18 h. For serum starvation, cells were washed twice with sterile phosphate-buffered saline (PBS) and then incubated in DMEM with 1% FBS for 24 h. For heat shock treatment, tissue-culture plates with growing cells were incubated in a water bath at 42°C for 2 h. To induce oxidative stress, cells were treated with 500 μM hydrogen peroxide at 37°C and 5% CO_2_ for 12 h. To induce acidosis, cells were treated with DMEM (pH 6.4) with 10% FBS at 37°C and 5% CO_2_ for 6 h. Actinomycin D (2 μg/ml) in dimethyl sulfoxide was used to study transcriptional regulation.

### Plasmids and stable transfection

The pHA-HIF-1α, pHA-HIF-1α (ΔODD), and pHA-HIF-1α (LCLL) expression constructs were obtained from Dr. L.E. Huang (University of Utah, USA) [47]. The Flag epitope-tagged human METTL4 cDNA cloned in pcDNA3 vector (pcDNA3-Flag-METTL4) was obtained from Prof. Chuan He (University of Chicago, USA). The Flag epitope-tagged Drosophila Jumu cDNA cloned in pEF vector (pEF-Flag-Jumu) was obtained from Dr. Dahua Chen (Chinese Academy of Sciences, Beijing, China) [45]. The human lncRNA *RP11-390F4.3* sequence was synthesized by Biotools (Taiwan) and cloned into pcDNA3.1 (+) vector (Life Technologies). The human lncRNA *NEAT1* expression vector was purchased from Addgene (USA). The oligonucleotides used to generate various plasmids and the methods to construct these plasmids were shown (Additional file 2: Tables S7 and S8). The generation of stable clones by calcium phosphate transfection or Lipofectamine was described [38, 48]. All the stable clones were established by transfection of plasmids as designated by the name of the clones.

### Establishment of METTL4 and RP11-390F4.3 knock-in cell lines

The knock-in cell lines were generated based on the Prime-Editing system [39] to make mutations at the catalytic site (287–290, DPPW to APAW) of METTL4 and the 6mA modification site on the *RP11-390F4.3* promoter (−231~−224, ACTTCCGA to CCGGCCGA). The all-in-one PE3 vector, pPE2.pPuro, was obtained from the National RNAi Core Facility (Academia Sinica, Taiwan). Briefly, the PE3 gRNA and pegRNA targeting *METTL4* or *RP11-390F4.3*, and RT-PBS containing the mutations were cloned into pPE2.pPuro to construct the pLAS-PE3-METTL4 and pLAS-PE3-RP11-390F4.3 plasmids (Additional file 2: Table S9). The plasmids were transfected respectively into FADU or BFTC909 cells and treated with 1.5 μg/ml puromycin for 3 days. Pure lines established from single cells were selected, and the genotypes were determined by PCR and DNA sequencing (the primers used are listed in Additional file 2: Table S9).

### Protein extraction, co-immunoprecipitation, Western blot analysis, RNA extraction, and quantitative real-time PCR

The extraction of total proteins from cells, co-immunoprecipitation assay, and Western blot analyses were performed as previously described [38, 48, 49]. The NE-PER™ Nuclear and Cytoplasmic Extraction Kit (Thermo Fisher Scientific) was used to extract the cytoplasmic and nuclear proteins. The characteristics of the antibodies used are listed in Additional file 2: Table S10. Total RNA purification, cDNA synthesis, and quantitative real-time PCR were performed as previously described [49]. The cytoplasmic/nuclear RNAs were purified from FADU or BFTC909 cells using the SurePrep Nuclear or Cytoplasmic RNA Purification Kit (Thermo Fisher Scientific) in accordance with the manufacturer’s protocol. The sequences of primers used in the real-time PCR experiment are listed in Additional file 2: Table S11.

### Immunofluorescence staining

Immunofluorescence staining followed by the acquisition of fluorescence images using confocal microscopy was performed as described previously [38, 49]. For METTL4 immunostaining, FADU, BFTC909, and KTCC28M cells were washed and fixed with 4% paraformaldehyde for 15 min at room temperature. Fixed cells were washed with PBS and permeabilized with PBS containing 0.1% Triton X-100 for 5 min at room temperature. Thereafter, cells were washed with PBS, blocked with 1% BSA in TBST buffer for 1 h, and incubated with anti-METTL4 antibody (1:500 dilution; HPA) at 4 °C overnight. Next day, cells were washed with PBS and incubated with the secondary antibody (1:500 dilution; goat anti-rabbit Alexa488, Abcam) for 1 h at room temperature followed by DAPI staining (1:10,000 dilution; Invitrogen). For 6mA immunostaining, the procedure was performed with minor modifications [11]. Briefly, the cells were stained with 100–200 nM MitoTracker Deep Red FM (Thermo Fisher) in HEPES buffer at 37°C for 30 min. Permeabilized cells were washed with PBS and then blocked for 1 h in blocking buffer (1% BSA in PBS containing 0.1% Tween, 40 μg/ml RNase A for RNA digestion, and 80 μg/ml DNase for DNA digestion). Cells were then incubated with 6mA antibody (1:500 dilution; Synaptic Systems) overnight at 4 °C. Next day, cells were washed with TBST three times and incubated with a secondary antibody (1:500 dilution; goat anti-rabbit Abberior STAR635). For double staining of mouse tissues, the tissue sections were incubated with anti-METTL4 (1:200 dilution; Abnova), anti-6mA (1:500 dilution; Synaptic Systems), or anti-HIF-1α (1:200 dilution; Abcam) antibodies. Finally, cells and tissues were stained with DAPI (1:10,000 dilution; Invitrogen) in PBS for 1 min at room temperature. The images were acquired using ImageXpress Micro Confocal System (Molecular Devices) and Leica TCS SP8 STED microscope. The images were processed using MetaXpress High-Content Image Acquisition and Analysis Software (MetaXpress version 6.5.4.532). Data analyses are conducted using MetaMorph software version 7.8.0.0 (Universal Imaging, USA) and Leica Application Suite X (LAS X version 3.5.5.19976) software. The cell counts from three independent replicates (*n*=150 cells) were pooled for further analysis. The hydrochloric acid antigen retrieval procedure was performed as described previously [7]. The antibodies used in immunofluorescence staining are listed (Additional file 2: Table S10).

### In vivo tumorigenicity assay

All animal experiment protocols were performed with the approval of the Institutional Animal Care and Use Committee of China Medical University in this study. Five-week-old BALB/c nu/nu mice were purchased from National Science Council Animal Center (Taipei, Taiwan). Briefly, 2 × 10^6^ viable cells were subcutaneously injected into the hind limbs of these BALB/c nu/nu mice, and then, the mice were euthanized after implantation at 30–35 days, and tumor incidence was monitored. Each experimental group contained at least five mice.

### In vitro migration/invasion and in vivo tail vein/orthotopic metastatic assays

The in vitro migration/invasion assays and in vivo tail vein/orthotopic metastatic assays were performed as per methods described previously [38, 49, 50]. Cells were injected into 6-week-old male, non-obese diabetic/severe combined immunodeficiency mice (NOD-SCID mice, National Science Council Animal Center, Taipei, Taiwan) through the tail vein (1 × 10^6^ cells) or orthotopic injection (1 × 10^5^ cells). The incidence of lung metastatic nodules was monitored 8–12 weeks after injection. Each experimental group contained at least five mice. All animal experiment protocols were performed with the approval of the Institutional Animal Care and Use Committee of China Medical University in this study.

### Fluorescence-activated cell sorting analysis

The procedure was performed as described previously with minor modifications [51, 52]. Briefly, prior to sacrifice and tumor excision, tumor-bearing mice (BALB/c nu/nu) with tumor volumes of ~350–500 mm^3^ were administered with 100 mg/kg pimonidazole (Hypoxyprobe™ Green Kit, HPI) intraperitoneally for 3 h and 1 mg/mouse Hoechst 33342 (Sigma-Aldrich) intravenously for 30 min. Tumor tissue was harvested and minced in PBS containing collagenase type I (Gibco Invitrogen Corp.) and dispase II (Gibco Invitrogen Corp.) and incubated for 40 min to prepare single-cell suspensions using gentle MACS Dissociator (Miltenyi Biotec). The cell suspension was filtered through a 70-μm sterile nylon mesh filter. Next, red blood cells were lysed with RBC lysis buffer (Invitrogen). After several washes, cells were stained with FITC-conjugated anti-pimonidazole (Hypoxyprobe™ Green Kit, HPI) in sorting buffer (PBS containing 0.5% BSA) following the manufacturer’s instructions. Cells were sorted using a FACSCalibur instrument (BD Bioscience). The hypoxic cell population was defined as Hoechst 33342+ and pimonidazole+ cells, and the normoxic cell population was defined as Hoechst 33342+ and pimonidazole− cells.

### Transient transfection, luciferase assays, and cell proliferation assays

The *METTL4* and *RP11-390F4.3* promoters were cloned; the reporter constructs are shown (Additional file 2: Tables S7 and S8). The *ZMIZ1* promoter (from −500 to −1 bp upstream of TSS) was inserted in front of the luciferase gene in pGL3-Basic vector to construct the *ZMIZ1* promoter-driven reporter plasmid (ZMIZ1-Luc). To construct an intron-containing luciferase reporter plasmid, the region from 24,990 to 25,469 bp of the *ZMIZ1* intron 1 that has the putative 6mA site (GGCACAAGCC) was inserted into the nucleotide 834 position of the firefly luciferase gene of ZMIZ1-Luc construct described above to mimic the artificial luciferase reporter containing an intron as described [53]. Under this construction, the new construct will contain the inserted *ZMIZ1* intron 1 sequence that has GT-AG sequence located at the splicing junction of artificial intron inside the luciferase open reading frame (named as ZMIC1-Luc-intron1-WT). Two other plasmids, ZMIZ1-Luc-intron1-Del (deletion of the putative 6mA site) and ZMIZ1-Luc-intron1-mut (mutation of GGCACAAGCC to GGCCCCCGCG), were generated to test the ability of the 6mA site to increase luciferase activity after methylation by METTL4. The reporter constructs are shown in Additional file 2: Tables S7 and S8. The reporter constructs were co-transfected into FADU cells with different expression vectors. A pRL-TK plasmid was used as an internal control. Luciferase activities were measured using the Dual-Luciferase™ reporter assay kit (Promega, Madison, WI) and further normalized with *Renilla* activity for transfection efficiency [54]. To prepare the in vitro 6mA pre-methylated reporter construct, the *RP11-390F4.3* promoter-driven reporter construct was prepared using ECOS™ 2163 competent cells (*Escherichia coli* K12 strain), and then incubated with METTL4 using in vitro DNA methylation assay as described below. Thereafter, the in vitro 6mA pre-methylated reporter construct was purified and the 6mA modification was confirmed via UPLC-ESI-MS/MS before it was co-transfected with other plasmids into the cells for luciferase assays. Cell proliferation assay were performed in 96-well plate (3.5 × 10^3^ cells/well) as described previously [11]. The proliferation of transfected cells at 24, 48, or 72 h was assessed using CellTiter 96 AQueous One Solution Reagent (Promega) according to the manufacturer’s instructions. The absorbance was measured at 490 nm using a microplate ELISA reader (TECAN Infinite 200).

### Lentivirus shRNA experiments

Biotools (Taiwan) designed the oligonucleotides for short hairpin RNA (shRNA) targeting *RP11-390F4.3*, which was cloned into a pLV2-U6-Puro vector. The sequence and target of *RP11-390F4.3* shRNAs (#1 and #2) are listed in Additional file 2: Table S12. shRNA-expressing lentiviral vectors (pLKO.1-puro or pLV2-U6-Puro) were generated in HEK293T cells as previously described [55]. The sequences and clonal names of plasmids against *METTL4*, *RP11-390F4.3*, *HIF-1*α, *HIF-1β*, *HIF-2*α, *ZMIZ1*, *KPNA7*, *N6AMT1*, and *U2 snRNA* are listed in Additional file 2: Table S12. These plasmids and the packaging plasmid pCMVΔR8.91 were provided by the National RNAi Core Facility of Academia Sinica (Taipei, Taiwan). For lentivirus production, HEK293T cells were transfected with 5 μg of lentiviral vectors expressing individual shRNA along with 5 μg of the packaging plasmid pCMVΔR8.91 and 0.5 μg of the envelope plasmid pMD.G. The viruses were collected 48 h after transfection. To prepare *METTL4*, *RP11-390F4.3*, *HIF-1*α, *HIF-1β*, *HIF-2*α, *ZMIZ1*, *KPNA7*, *N6AMT1*, and *U2 snRNA* knockdown cells, cells were infected with the corresponding lentiviruses for 24 h, and the stable clones were then generated by selection with appropriate antibiotics.

### Genomic and mitochondria DNA extraction

Briefly, harvested cells (1 × 10^7^) were washed with PBS and suspended in ice-cold pre-lysis buffer (20 mM HEPES [pH 7.4], 10 mM KCl, 2 mM MgCl_2_, 1 mM EDTA, and 1 mM EGTA). After incubation on ice for 15 min, the cells were ruptured with a 27-gauge needle (BD Precision Glide needle) 15 times and then allowed to stand on ice for 20 min. After centrifugation at 720*×g* for 5 min at room temperature, pellets were collected and genomic DNA was extracted using the EasyPrep Genomic DNA Extraction Kit (Tools, Taiwan) according to the manufacturer’s protocol. Genomic DNA concentrations were measured using a fluorometer (Qubit® 3.0; Thermo Fisher Scientific). The efficiency of removing mitochondrial DNA from the genomic DNA extraction step was quantified by quantitative real-time PCR analysis using mitochondrial and nuclear-specific primers [11, 56]. The primers used are listed in Additional file 2: Table S11. Mitochondrial DNA extraction was performed as previously described [11, 56]. The quality of the extraction was assessed by comparing the fold change of mitochondrial DNA versus genomic DNA using the Δ*C*_t_ method.

### 6mA dot blot assays

The 6mA dot blot assay was performed as described previously, with minor modifications [4, 5]. Briefly, the DNA was diluted using nuclease-free H_2_O to a final concentration of 150 ng/μl, denatured at 95°C for 10 min, and then loaded onto Hybond N+ membranes (GE Healthcare Life Sciences). The membranes were air-dried, baked at 78°C for 25 min, and then blocked in 5% non-fat dry milk in PBS containing 0.1% Tween 20 (PBST) at 37°C for at least 1 h. Thereafter, the membranes were washed with PBST, probed with anti-6mA antibody (Synaptic Systems), probed with the secondary antibody, and developed. Methylene blue staining was used as the loading control.

### Quantification of 6mA in gDNA via ultra-performance liquid chromatography with electrospray ionization tandem mass spectrometry (UPLC-ESI–MS/MS)

The procedure was performed as described with minor modifications [3]. Three hundred nanograms of DNA was denatured in 20 μl of nuclease-free H_2_O at 95°C for 10 min, rapidly incubated on ice, and digested with 2 U nuclease P1 (Wako USA, 145-08221) in 10 mM ammonium acetate [pH 5.3] at 42°C. After 18 h, 2 μl of phosphodiesterase I (Sigma-Aldrich, P3243-1VL) and 3.4 μl of 1 M NH_4_HCO_3_ were added to the digested DNAs, and digestion was continued at 37°C for 4 h. Thereafter, the digested products were treated with 2 U of alkaline phosphatase (Sigma, P5931-500UN) at 37°C for at least 4 h and diluted 1:2 with nuclease-free H_2_O followed by filtering through a 0.22-μm filter (Millipore, SLGVR04NL). The resulting deoxyribonucleoside samples were analyzed via UPLC-ESI-MS/MS. Deoxyribonucleoside standards (Carbosynth), deoxyadenosine (dA), and *N*^6^-deoxyadenosine (6mA) were used for constructing daughter ion scan spectra to identify digested DNA products and also for quantitative calculation by multiple reaction monitoring (MRM). Separation of nucleosides was carried out by reverse phase UPLC on an Acquity UPLC® BEH C18 1.7 μm column, 2.1 mm × 50 mm (Waters, Milford, USA) at 30°C. Mass spectra and chromatograms were acquired in positive ESI mode, using Waters Xevo TQ-XS mass spectrometer. The nucleosides in digested DNAs were determined by LC retention plus MS/MS spectra and quantified based on the ion mass transitions; 6mA (m/z): 266 to 150 and dA (m/z): 252 to 136 in MRM mode. The 6mA/dA ratio of each sample was calculated with quantified 6mA and dA values in accordance with the calibration curves obtained from dA and 6mA standards. The representative chromatogram as quantified by their integrated area in the corresponding chromatogram was shown as peaks for samples tested.

### In vitro DNA and RNA methylation assays

Mammalian HEK293T cells were transfected with either pcDNA3-Flag-METTL4 WT or Flag-METTL4 MUT plasmid, and then the overexpressed proteins were purified through immunoprecipitation with anti-FLAG M2 affinity gel (Sigma-Aldrich) according to the manufacturer’s protocol. Enzyme activity of METTL4 was analyzed as described previously with slight modifications [57]. Briefly, in a 50 μl total reaction, 500 ng of DNA substrate was incubated with METTL4 (50–100 μM) which was prepared via immunoprecipitation. The reaction buffer comprised 1.5 mM S-adenosyl-L-methionine (SAM), 80 mM KCl, 1.5 mM MgCl_2_, 2 mM CaCl_2_, 10 mM DTT, 4% glycerol, and 15 mM HEPES [pH 7.9]. After incubating the reaction mixture at 16°C for 18 h, the reaction was terminated by heat inactivation at 65°C for 5 min. Thereafter, DNA was cleaned, purified, and the 6mA modification was analyzed via UPLC-ESI-MS/MS, as described above. The 6mA/dA ratio of each sample was normalized with the untreated probe alone. The probe sequences used for the in vitro DNA methylation assay are listed in Additional file 2: Table S13. For the in vitro RNA methylation assay, *U2 snRNA* and *RP11-390F4.3* RNA molecules were generated with an in vitro transcription kit (Thermo Fisher Scientific) using *U2 snRNA* and *RP11-390F4.3* expression constructs, respectively, which were then purified using the RNeasy Mini Kit (Qiagen, Valencia, CA). Each RNA substrate (300 ng) was incubated with METTL4 (50–100 μM), which was purified via immunoprecipitation, in a 50 μl total reaction with reaction buffer containing 1.5 mM SAM, 80 mM KCl, 1.5 mM MgCl_2_, 0.2 U/μl SUPERaseIn, and 15 mM HEPES [pH 7.9]. After incubation at 30°C for 2 h, the reaction was terminated by heating at 65°C for 5 min. The RNA was then purified with the RNeasy Mini Kit (Qiagen), and the m^6^Am modification was analyzed via UPLC-ESI-MS/MS as described below.

### Quantification of m^6^Am in U2 snRNA and RP11-390F4.3 via UPLC-ESI–MS/MS

Isolation of *U2 snRNA* or *RP11-390F4.3* from cells for UPLC-ESI-MS/MS analysis was performed as previously described [58]. Briefly, the synthetic biotin-labeled probes against specific RNAs were incubated with total RNA and then purified using streptavidin T1 beads. The biotin-labeled probes used are listed in Additional file 2: Table S14. Five hundred nanograms of *U2 snRNA* or *RP11-390F4.3* were treated with 2 units of nuclease P1 (Sigma-Aldrich) at 37°C in 50 μl of reaction buffer (20 mM sodium acetate [pH 5.3], 5 mM ZnCl_2_, and 50 mM NaCl). After 4 h, 10 units of antarctic phosphatase (NEB) were added, and the reaction continued for another 4 h. The resulting nucleosides were analyzed by UPLC-ESI-MS/MS with the same settings used to determine 6mA. Nucleoside standards (Toronto Research Chemicals), adenosine, and *N*^6^,2′-*O*-dimethyladenosine (m^6^Am) were used to construct daughter ion scan spectra to identify digested RNA products and for quantitative calculation by multiple reaction monitoring (MRM). The nucleosides of the digested RNAs were identified using LC retention with MS/MS spectra and quantified based on the ion mass transitions; m^6^Am (m/z): 296 to 150 and adenosine (m/z): 268 to 136 in MRM mode.

### Chromatin immunoprecipitation (ChIP), quantitative chromatin immunoprecipitation (qChIP) and methylated DNA immunoprecipitation (MeDIP) assays

Herein, ChIP and qChIP were performed as described previously [49]. Briefly, the cells were crosslinked, sonicated, and then immunoprecipitated with the antibody. For the re-ChIP assay, after the first ChIP reaction, the DNA–protein complexes were washed and eluted by incubation at 37°C with 25 μl of 15 mM dithiothreitol (DTT) for 30 min. After centrifugation, the supernatant was collected and subsequently diluted with nuclei lysis buffer and subjected to the ChIP procedure again. IgG was used as a negative control. The DNA samples were quantified by quantitative real-time PCR using SYBR® Green PCR Master Mix (Applied Biosystems). The primers and antibodies used in the ChIP assay are listed in Additional file 2: Tables S15 and S10, respectively. MeDIP was performed as previously described [3, 59]. Genomic DNA was isolated from the harvested cells and sonicated using Bioruptor. The fragmented DNA was denatured and then immunoprecipitated with anti-6mA antibodies. The 6mA antibody used is listed (Additional file 2: Table S10), and the primers used in the quantitative MeDIP assay are listed (Additional file 2: Table S15). The qChIP and the qMeDIP values were calculated as previously described [49, 60].

### Chromatin isolation by RNA purification (ChIRP)

ChIRP was performed as described with minor modifications [44]. *RP11-390F4.3* and *lacZ* probes were designed using an online program at “www.singlemoleculefish.com” (Additional file 2: Table S16). Anti-sense DNA probes were labeled with a BiotinTEG at the 3′ end. Cells were washed and trypsinized for 5 min at room temperature. The harvested cells were washed with PBS, crosslinked in 20 ml of 1% glutaraldehyde/PBS on an end-to-end rotator for 10 min at room temperature, and then quenched by adding glycine to a final concentration of 125 mM for 5 min at room temperature. The cells were pelleted by centrifuging at 2000*g* for 5 min at 4°C. This was followed by two PBS washes, and the pellets were resuspended in the lysis buffer (10x mass pellet in grams; 50 mM Tris-HCl [pH 7.0], 10 mM EDTA [pH 8.0], 1% SDS, and protease inhibitor (Sigma)) with freshly added 1 mM of PMSF (Sigma) and Superase IN. The lysate was sonicated by Bioruptor at 4°C for at least 30 min (30 s ON, 30 s OFF) until the sample was no longer turbid. After centrifugation at 16,100*g* for 10 min at 4°C, the supernatant was subjected to ChIRP. After putting aside samples for DNA and RNA input, the lysates were diluted in 2 volumes of hybridization buffer (50 mM Tris-HCl [pH 7.0], 750 mM NaCl, 1 mM EDTA [pH 8.0], 1% SDS, 15% formamide, and protease inhibitors) with freshly added 1 mM of PMSF and Superase IN. Pools of probes (100 pmol) were added and incubated for 4 h at 37°C. The C-1 magnetic beads (Invitrogen) were washed twice with the lysis buffer and added into the lysate for 30–60 min at 37°C. Next, the beads were washed five times for 5 min at 37°C with mixing in pre-warmed washing buffer (2× saline sodium citrate [SSC], 0.5% SDS, and protease inhibitors) with freshly added 1 mM of PMSF and Superase IN. After the last wash, the beads were resuspended in 1 mL of washing buffer and then separated for RNA (100 μl) and DNA (900 μl) purification as described in the protocols for RNA extraction, ChIP, and qChIP. The primers used in qChIRP assay are listed (Additional file 2: Tables S11 and S15).

### RNA sequencing (RNA-seq)

In brief, RNA-Seq libraries were prepared by Kapa HyperPrep Kit with RoboErase and were sequenced using Illumina NextSeq 550 to obtain 150 bp paired-end reads.

### Chromatin immunoprecipitation followed by sequencing (ChIP-seq)

After DNAs were obtained from ChIP procedure, libraries were constructed by using KAPA HyperPrep Kit. Libraries were sequenced using Illumina NextSeq 550 to obtain 75 bp single-end reads.

### Genomic DNA 6mA-ChIP-exo-seq 5.0

6mA ChIP-exo 5.0 was performed according to the previous reported procedure with some modifications to increase the yield for final library construction [11]. Briefly, 15–20 μg of fragmented genomic DNA was incubated with anti-6mA antibody (Synaptic Systems) at 4°C overnight in 1 × IP buffer (150 mM NaCl, 0.1% IGEPAL CA-630, 10 mM Tris-HCl; pH 7.4). The mixture was irradiated with UV 254 nm with 0.15 mJ/cm^2^ energy for 6 times. The crosslinked samples were pulled down at 4°C for 2 h, using 80 μl pre-blocked Dynabeads Protein A slurry (Invitrogen). Crosslinked DNAs on beads were washed with FA lysis buffer (50 mM Hepes-KOH, pH 7.5, 150 mM NaCl, 2 mM EDTA, 1% Triton, 0.1% sodium deoxycholate), NaCl lysis buffer (50 mM HEPES-KOH, pH 7.5, 500 mM NaCl, 2 mM EDTA, 1% Triton X-100, 0.1% sodium deoxycholate), LiCl buffer (100 mM Tris-HCl, pH 8.0, 500 mM LiCl, 1% NP-40, 1% sodium deoxycholate), and 10 mM Tris-HCl buffer at 4°C followed by enzyme reaction on beads, including A tailing (15 U Klenow Fragment, -exo (NEB), 1 × NEBuffer 2, and 100 μM dATP) at 37°C for 30 min, the first adapter ligation and kinase reaction (1200 U T4 DNA ligase, 10 U T4 PNK, 1 × NEBNext Quick Ligation Buffer, and 375 nM adapter) at 25°C for 1 h, fill-in reaction (10 U phi29 polymerase, 1 × phi29 reaction buffer, 2 × BSA, and 180 μM dNTPs) at 30°C for 20 min, and lambda exonuclease digestion (10 U λ exonuclease, 1 × λ exonuclease reaction buffer, 0.1% Triton X-100, and 5% DMSO) at 37°C for 30 min. DNAs were then released from beads with proteinase K digestion (30 μg Proteinase K, 25 mM Tris-HCl, pH 7.5, 2 mM EDTA, 200 mM NaCl, and 0.5% SDS) at 56°C overnight and then purified by using Agencourt AMPure XP (Beckman Coulter) with 3× beads clean up. Purified DNAs were subjected to splint ligation (1200 U T4 DNA ligase, 1 × Quick Ligase Buffer, 375 nM splint adapter). The product was purified using AMPure XP with 3× beads clean up and amplified by using PCR primers in TruSeq small RNA preparation kit and Phusion Master Mix with HF buffer (NEB). After the first 5 cycles of pre-amplification, 5 μl of pre-amplified samples was subjected to qPCR reaction to avoid over amplification according to the previous method [61]. Libraries were subjected to high-throughput sequencing by using NextSeq 550 with 75 bp single-end sequence.

### DNA whole genome amplification (WGA) protocol for 6mA-ChIP-exo-seq

Whole genome amplified DNA was generated by RPEPLI-g midi kits (Qiagen) with 5 ng input genomic DNA. After amplification, amplified DNA was purified by 1× Ampure XP beads followed by 6mA-ChIP-exo-seq.

### High-throughput sequence analysis

Before alignment, we trimmed all the sequenced reads by Trim galore. For RNA-seq, we aligned the reads to hg19 with HISAT2 [62]. After the alignment, gene abundances were done by htseq-count, and edgeR was used for differential gene analysis or Gene Set Enrichment Analysis was used for searching enrichment gene set in our RNA-seq data [63, 64]. For ChIP-seq and ChIP-exo-seq data, we aligned the reads to hg19 with Bowtie [65]. After the alignment, we filtered the aligned reads by mapping quality and removed the duplicated by picard. To get the enrichment region for all types of sequence, we applied MACS2 for peak calling [66]. Normalized signal files and profiles cross the genome were done by deeptools for data visualization [67]. Peak to gene correlation was performed by ChIPseeker with the annotation file of Ensembl version 87 [68]. Functional gene annotation was performed by gene ontology analysis with HOMER (Hypergeometric Optimization of Motif Enrichment) software [69].

### 6mA-ChIP-exo-seq quality control and genome-wide differential 6mA region analysis

According to the quality control results of each sample, we used 3 out of 5 hypoxia replicates for genome-wide analysis in different comparisons (comparison 1 or 2) (Additional file 1: Fig. S4h-j and Table S6). Due to the low complexity of 6mA ChIP-exo-seq data, we used a different pipeline to obtain enriched 6mA regions. This pipeline was based on previous established pipeline for 6mA signals counting by genome windows with application of quantile-adjusted conditional maximum likelihood method. We defined 3-kb windows across the genome and counted the signals from the de-duplicated aligned reads from each condition (i.e., comparing hypoxia vs. normoxia or METTL4-si vs. scrambled-si under hypoxia with edgeR [63]). For differential analysis, 6mA signal values in each condition were normalized with input sequence control. To define the gain-of-6mA regions regulated by hypoxia and METTL4, we applied the threshold of significant *p*-value < 0.05 under hypoxia vs. normoxia with the cut-off input-normalized log2 fold change of hypoxia vs. normoxia (> log2(1.1)) and METTL4 knockdown vs. control undergoing hypoxic status (< −log2(1.1)). Through this analysis, we identified 8268 regions in comparison 1 (hypoxia replicate-1,2,3) and 76,695 regions in comparison 2 (hypoxia replicate-1,4,5) (Additional file 1: Fig. S4i). To exclude the potential false discovered gain-of-6mA regions, we calculated the input-normalized log2 fold change of 6mA signals in hypoxia vs. WGA and considered input-normalized log2 fold change > log2(1.1) as hypoxia/WGA positive gain-of-6mA regions. By overlapping gain-of-6mA regions of different comparisons and hypoxia/WGA 6mA-positive regions, we generated 3673 gain-of-6mA regions co-regulated by hypoxia and METTL4 through multiple background subtractions for downstream analysis (Additional file 1: Fig. S4i, j).

### Motif calculation

Motifs were calculated by HOMER [69] from comparison of each group of hypoxia 6mA-ChIP-exo-seq datasets as mentioned above (Comparison 1 used hypoxia-1, -2, -3 groups; Comparison 2 used hypoxia-1, -4, -5 groups) (Additional file 2: Table S6). The enriched signals located in hypoxia-induced METTL4 dependent gain-of-6mA regions were used for identifications of de novo motif by using the intersection between comparison 1 and comparison 2 (Additional file 1: Fig. S4i, j). WGA control signals from comparison 1 were used as background to exclude the sequencing noise. The *p*-value of motifs was calculated accordingly.

### Analysis of RNA alternative splicing

RNA alternative splicing analysis was performed by rMAST [70]. After the quantification of spliced events, the results were input to maser, a R package, for downstream analysis. Low counts of spliced events were filtered by the cut-off mean junction counts < 10. Analysis of differential splicing events was performed with each comparison (hypoxia vs normoxia or METTL4-si vs scrambled-si under hypoxia). Significant spliced events were generated with the cut-offs *p*-value < 0.05, an absolute change in percent spliced in (|ΔPSI|) ≥ 0.05.

### Calculation of lncRNA copy number per cell

LncRNA copy number was calculated as described previously [71, 72]. A linear standard amplification curve of Ct values was generated by qPCR using a dilution series of *RP11-390F4.3* or *NEAT1* plasmid DNA templates with known concentrations, corresponding to the regions amplified during qRT-PCR. The qRT-PCR values of *RP11-390F4.3* or *NEAT1* transcripts from 500,000 cells were determined under normoxia and hypoxia conditions. The Ct values were then fitted on a standard amplification curve, and the corresponding total transcripts were divided by 500,000 to calculate the number of *RP11-390F4.3* or *NEAT1* transcripts per cell. The plasmid DNA templates used are listed in Additional file 2: Table S8, and the primers used are listed in Additional file 2: Table S11.

### Single-molecule RNA fluorescence in situ hybridization (RNA-FISH)

Single-molecule RNA-FISH was performed as described previously with minor modifications [72, 73]. The RNA-FISH probes targeting *RP11-390F4.3* were designed using LGC Biosearch Technologies online software (Stellaris® Probe Designer version 4.2; https://www.biosearchtech.com/support/education/stellaris-rna-fish) and were purchased from LGC Biosearch Technologies. Forty-eight fluorescein-conjugated Stellaris RNA-FISH probes specific to *RP11-390F4.3* are listed in Additional file 2: Table S17. Cells were fixed with 4% formaldehyde/5% acetic acid solution for 15 min at room temperature and then washed with PBS. Fixed cells were treated with 1% pepsin and subsequently dehydrated with a gradient of 70–100% ethanol. Cells were incubated with RNA-FISH probes diluted in hybridization buffer (100 mg/ml dextran sulfate, 0.2 mg/ml bovine serum albumin, and 10% formamide in 2× saline sodium citrate buffer (SSC)) at 55 °C. After 4 h, cells were washed three times with 0.1× SSC at 65 °C and then mounted using Prolong Gold Antifade Reagent with DAPI (Invitrogen). Images were captured using ImageXpress Micro Confocal System (Molecular Devices). Images were visualized using the MetaXpress High-Content Image Acquisition and Analysis Software (MetaXpress version 6.5.4.532).

### DNA electrophoretic mobility shift assay (EMSA)

The DNA EMSA experiment was performed using a LightShift® Chemiluminescent EMSA Kit (Thermo Scientific) by following the manufacturer’s protocol. The HIF-1α and Jumu proteins were purified from the HEK293T cells transiently transfected with HIF-1α and Jumu plasmids using anti-HA and anti-Flag conjugated agarose (Sigma-Aldrich). Each DNA EMSA reaction mixture was set up in a final volume of 20 μl containing the annealed double-stranded DNA probe (20 nM) and HIF-1α /Jumu protein (500 ng) in the binding buffer of the LightShift® Chemiluminescent EMSA Kit (Thermo Scientific). The biotin-labeled probes used for the DNA EMSA assay are listed (Additional file 2: Table S18).

### Study population and sample collection

One hundred and forty-nine upper tract urothelial carcinoma (UTUC) patients who underwent surgical resection at China Medical University Hospital (CMUH, Taiwan) between February 2004 and April 2015 were retrospectively analyzed. The study was approved by the Institutional Review Board of CMUH, Taiwan (See Additional file 2: Table S1 for clinical characteristics of the UTUC patients). Primary tumor samples and the corresponding non-cancerous matched tissue were obtained during surgery. A high-density tissue microarray was constructed. Tumor histology and grades were confirmed by an experienced pathologist.

### Immunohistochemistry, RNAScope assay, and scoring

The immunohistochemistry of METTL4, 6mA, HIF-1α, and ZMIZ1 was assessed by ImageScope software analysis H-scoring system (Aperio Technologies, Vista, CA, USA). The staining intensity was assigned a four-tiered grading, as follows: 0: negative, 1: weak, 2: medium, and 3: strong staining. The H-scores ranged from 0 to 300. The IHC staining of METTL4, 6mA, HIF-1α, and ZMIZ1 was assessed semi-quantitatively by assigning an H-score, which was defined by multiplying the percentage of positive-stained tumor cells (from 0 to 100) by staining intensity. For RNAScope assay, the RNAScope probe specific to *RP11-390F4.3* was designed by Advanced Cell Diagnostics. The detection of *RP11-390F4.3* expression was performed using the RNAscope 2.5 High Definition (HD)—RED Assay according to the manufacturer’s instructions (Advanced Cell Diagnostics). The images were acquired with ZEISS Axio Vert.A1 microscope and assessed by ImageScope software.

### Statistical analysis

Unless otherwise mentioned, all samples were assayed in triplicates. For in vitro analyses, each experiment was repeated at least three times. For in vivo analyses, each group of experiment used at least five mice. The error bars represented the standard deviation (SD). Student’s *t* test was used to compare two groups of independent samples. For clinical samples, Student’s *t* test was applied for comparison of dichotomous variables. The ROC curve analysis was used to find the optimal cut-off value of the H-scores for METTL4, 6mA, HIF-1α, and ZMIZ1. Associations between categorical variables and expression patterns of METTL4, 6mA, HIF-1α, ZMIZ1, and *RP11-390F4.3* were examined using Pearson’s chi-square test. The Kaplan-Meier estimation was used to plot overall and disease-free survival curve and compared using the log-rank test. Cox proportional hazard model was applied in univariate and multivariate survival analysis to test independent prognostic factors. The statistical analysis was performed using the IBM SPSS Statistics software (version 22, SPSS, Chicago, IL, USA) or R (version 3.4). The level of statistical significance was set at 0.05 for all tests.

## Supplementary Information

**Additional file 1: **Supplemental figures. **Figure S1.** Induction of 6mA levels and activation of METTL4 by hypoxia/HIF-1α, and other characterizations of m^6^Am, N6AMT1, HIF-2α, and HIF-1β. **Figure S2.** The critical role of METTL4 in inducing 6mA levels, EMT/EMT regulators, in vitro migration/invasion activity, in vivo metastasis/tumorigenicity, and increased expression of METTL4, 6mA and HIF-1α in the tumor samples of UTUC patients. **Figure S3.** Characterizations of the enzymatic activity and nuclear localization of METTL4 in its ability to induce gene expression and EMT phenotypes. **Figure S4.** Analyses of RNA-seq datasets, characterizations of the levels of EMT regulators, signaling connection of METTL4 and lncRNA *RP11-390F4.3,* heatmap analysis of hypoxia-activated lncRNAs, analyses and profiling of 6mA signals and their consensus sequences, profiling and confirmation of differential RNA splicing events, and characterizations of *U2 snRNA* functions. **Figure S5.** Characterizations of lncRNA *RP11-390F4.3* by various assays, ChIRP peaks of three different EMT regulator genes, and TCGA survival analysis of head and neck cancer patient groups with high lncRNA *RP11-390F4.3* levels*.* **Figure S6.** Characterizations of ZMIZ1 as a HIF-1α co-activator, and characterizations of 6mA genomic sites methylated by METTL4 by various assays.

**Additional file 2: **Contains all supplemental tables. **Table S1.** Relationship of METTL4 expression with UTUC pathologic parameters. **Table S2.** Univariate survival analysis. **Table S3.** Multivariate survival analysis. **Table S4.** Correlation of METTL4 expression with ZMIZ1 and *RP11-390F4.3* expressions in UTUC patients. **Table S5.** Correlation of 6mA expression with ZMIZ1 and *RP11-390F4.3* expressions in UTUC patients. **Table S6.** Analysis of 6mA ChIP-exo seq datasets from different comparisons. **Table S7.** Sequence of the oligonucleotides and restriction enzymes used for plasmid construction. **Table S8.** Schemes of plasmid constructions for constructs used. **Table S9.** Sequences of primers for *METTL4* and *RP11-390F4.3* knock-in experiments. **Table S10.** List of proteins tested by and characteristics of the corresponding antibodies. **Table S11.** Sequence of the primers for real-time PCR. **Table S12.** Lentivirus used in RNAi experiments. **Table S13.** Sequence of the oligonucleotides for in vitro DNA methylation assay. **Table S14.** Sequence of the probes for isolation of *U2 snRNA* and *RP11-390F4.3*. **Table S15.** Sequence of the primers for ChIP, ChIRP and MeDIP assays. **Table S16.** Sequence of the *RP11-390F4.3* and *lacZ* probes for ChIRP assay. **Table S17.** Sequence of the probes for RNA FISH assay. **Table S18.** Sequence of the probe for DNA EMSA.

**Additional file 3:** Contains images of full Western blots. The original uncropped Western blot images used in Figures 1, 2, 3, 4, 5 and 6, Figures S1-4, and Figure S6.

**Additional file 4:** Review history.

## Data Availability

Sequencing datasets are available from Gene Expression Omnibus under accession number GEO: GSE171115 [74] and GSE171116 [75]. All data generated or analyzed during this study are included in this article and the Additional files. All cell lines used in this study have been authenticated and will be available upon request. All reagents will also be available upon request.
