# Supplementary material for: METTL4-mediated nuclear N6-deoxyadenosine methylation promotes metastasis through activating multiple metastasis-inducing targets
Source: Genome Biol. 2022 Dec 2;23:249. doi: 10.1186/s13059-022-02819-3 (PMC9716733; doi:10.1186/s13059-022-02819-3)
Supplement: Supplementary file 1 — Additional file 1: Supplemental figures. Figure S1. Induction of 6mA levels and activation of METTL4 by hypoxia/HIF-1α, and other characterizations of m6Am, N6AMT1, HIF-2α, and HIF-1β. Figure S2. The critical role of METTL4 in inducing 6mA levels, EMT/EMT regulators, in vitro migration/invasion activity, in vivo metastasis/tumorigenicity, and increased expression of METTL4, 6mA and HIF-1α in the tumor samples of UTUC patients. Figure S3. Characterizations of the enzymatic activity and nuclear localization of METTL4 in its ability to induce gene expression and EMT phenotypes. Figure S4. Analyses of RNA-seq datasets, characterizations of the levels of EMT regulators, signaling connection of METTL4 and lncRNA RP11-390F4.3, heatmap analysis of hypoxia-activated lncRNAs, analyses and profiling of 6mA signals and their consensus sequences, profiling and confirmation of differential RNA splicing events, and characterizations of U2 snRNA functions. Figure S5. Characterizations of lncRNA RP11-390F4.3 by various assays, ChIRP peaks of three different EMT regulator genes, and TCGA survival analysis of head and neck cancer patient groups with high lncRNA RP11-390F4.3 levels. Figure S6. Characterizations of ZMIZ1 as a HIF-1α co-activator, and characterizations of 6mA genomic sites methylated by METTL4 by various assays. [file 13059_2022_2819_MOESM1_ESM.pdf]

Supplementary Information

METTL4-mediated nuclear N6-deoxyadenosine methylation promotes metastasis through activating multiple metastasis-inducing targets

Kai-Wen Hsu[1,2,3], Joseph Chieh-Yu Lai[1,4], Jeng-Shou Chang[1], Pei-Hua Peng[1], Ching-Hui Huang[1], Der-Yen Lee[5], Yu-Cheng Tsai[2], Chi-Jung Chung[6], Han Chang[7], Chao-Hsiang Chang[8], Ji-Lin Chen[9], See-Tong Pang[10], Ziyang Hao[11,12], Xiao-Long Cui[11], Chuan He[11,13], Kou-Juey Wu[1*]

[*]**Corresponding author. Email:** wukj@cgmh.org.tw

This file includes:

Supplementary Figures (Figures S1-S6)

Legends to Supplementary Figures

Supplementary Figures
Figure S1

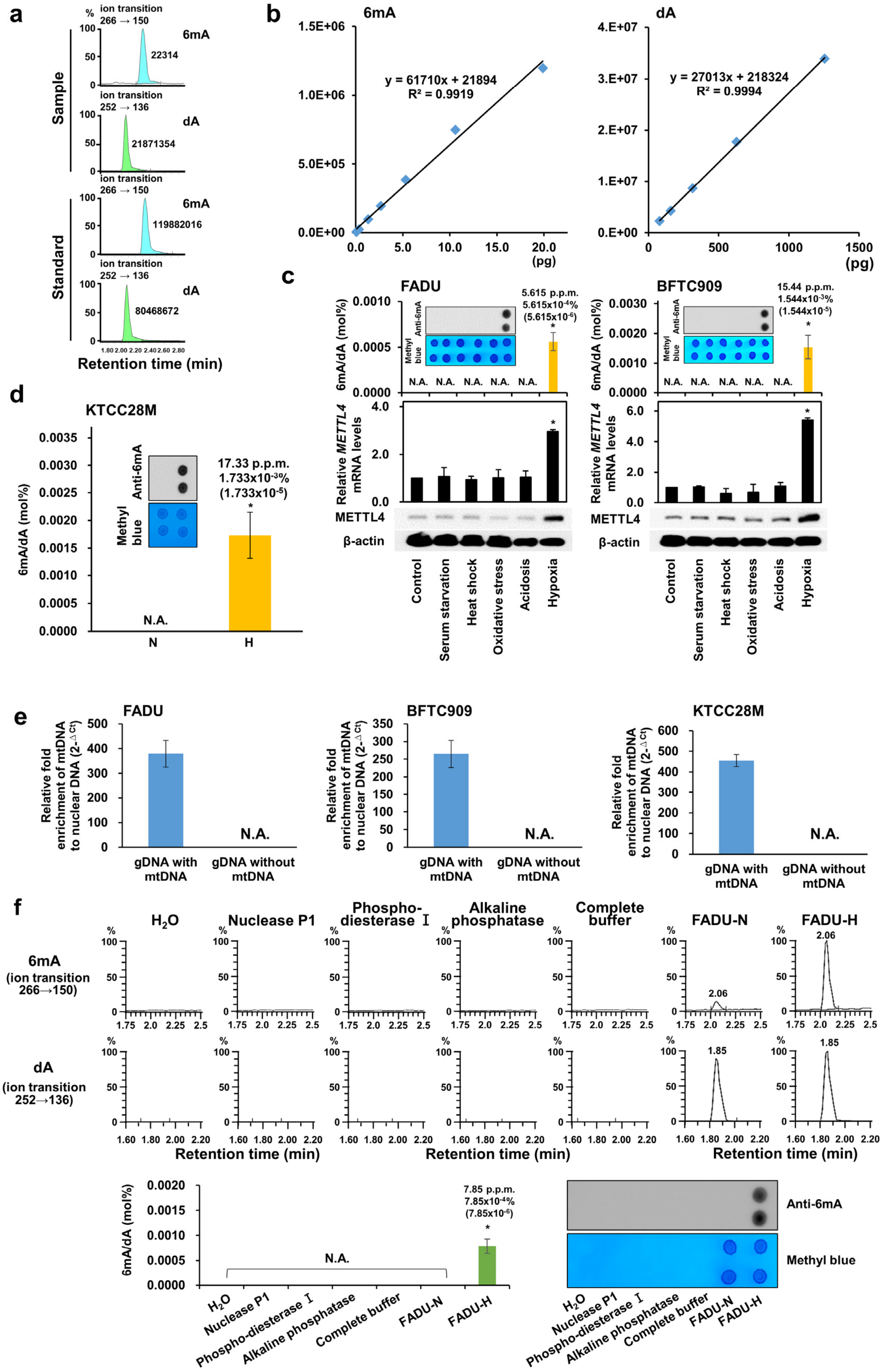

g

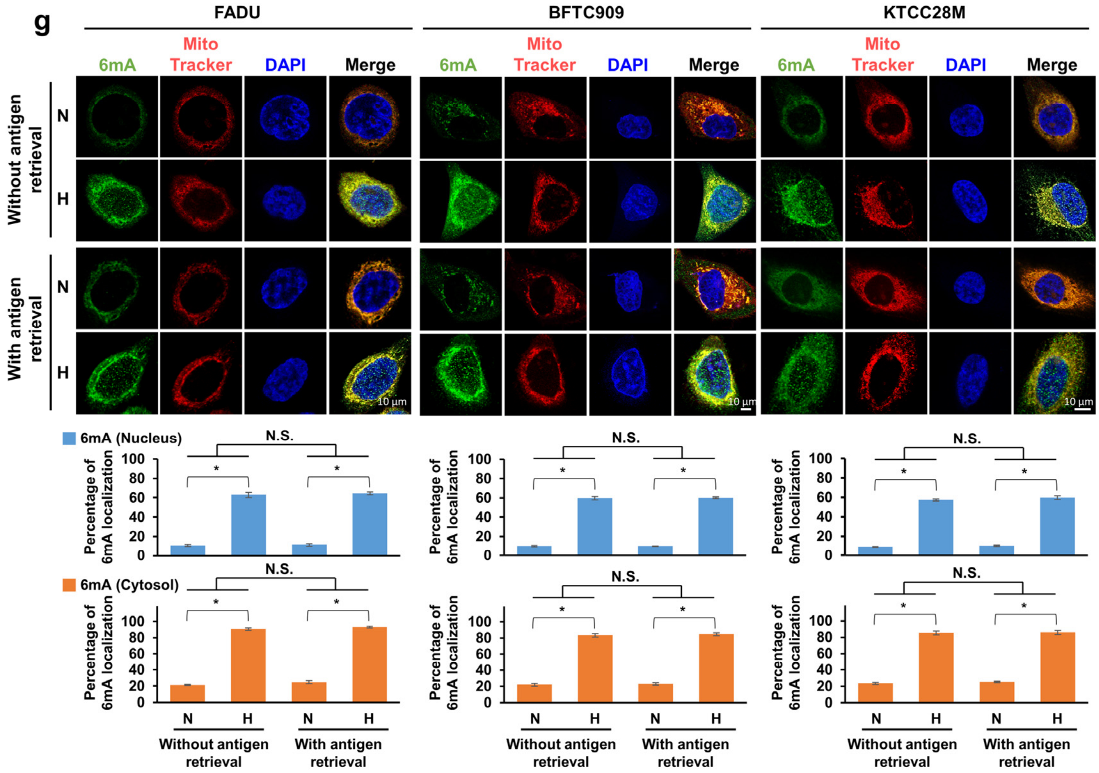
FADU
BFTC909
KTCC28M
6mA
Mito Tracker
DAPI
Merge
Without antigen retrieval
With antigen retrieval
N
H
10 μm
6mA (Nucleus)
6mA (Cytosol)
N.S.
*
Percentage of 6mA localization
0
20
40
60
80
100
Without antigen retrieval
With antigen retrieval

h

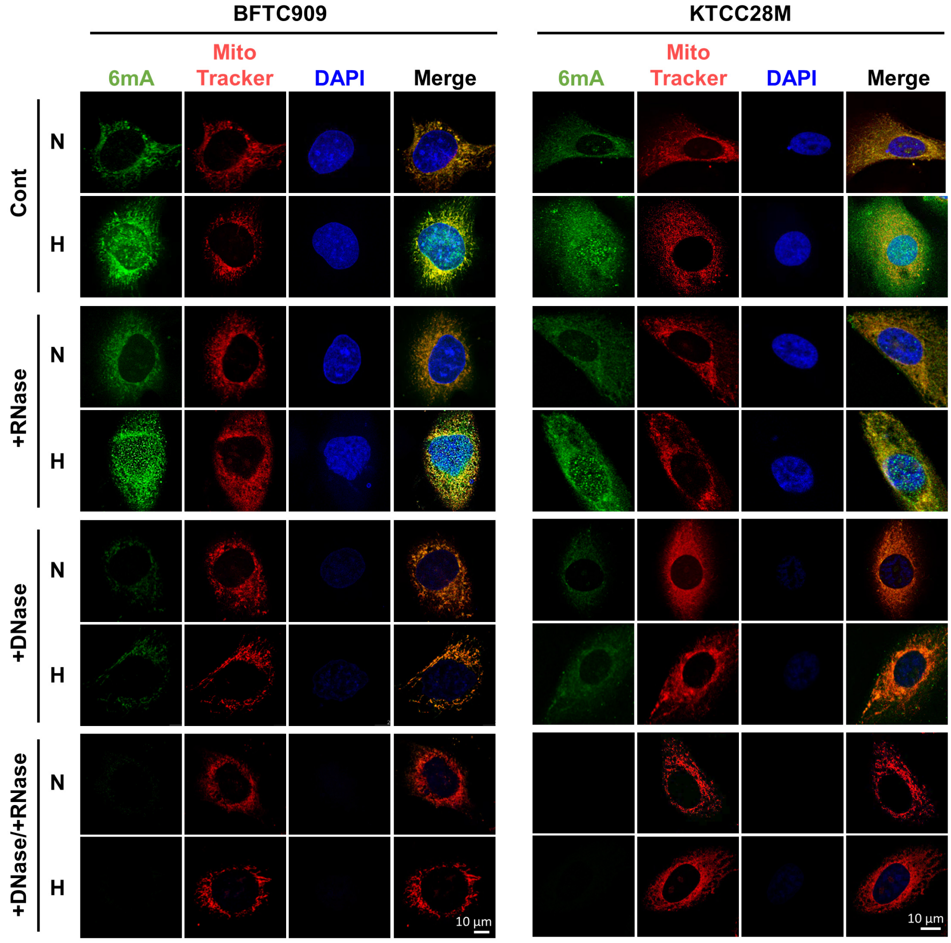
BFTC909
KTCC28M
6mA
Mito Tracker
DAPI
Merge
Cont
+RNase
+DNase
+DNase/+RNase
N
H
10 μm

i

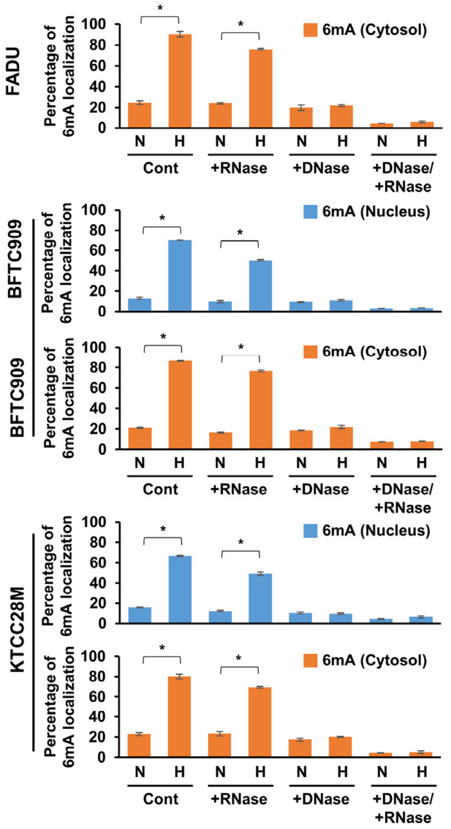
FADU
BFTC909
BFTC909
KTCC28M
Percentage of 6mA localization
6mA (Cytosol)
6mA (Nucleus)
6mA (Cytosol)
6mA (Nucleus)
6mA (Cytosol)
N H N H N H N H
Cont
+RNase
+DNase
+DNase/ +RNase
*

j

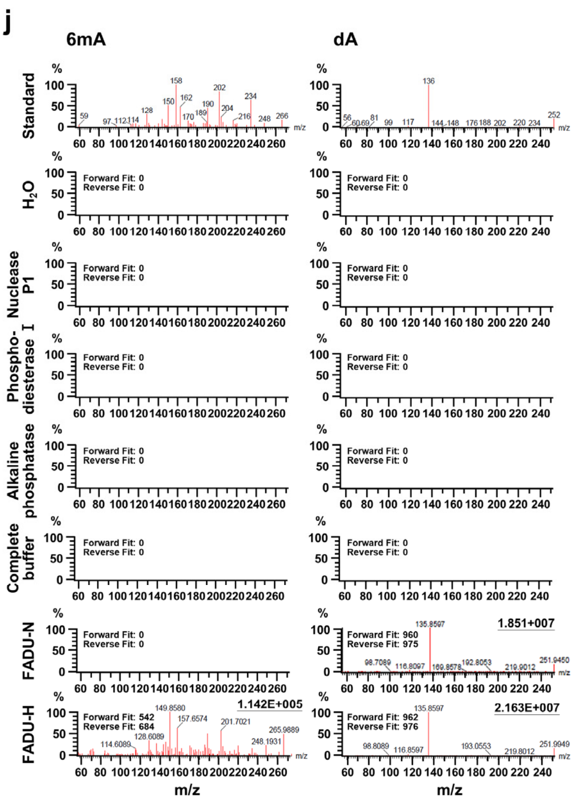
6mA
dA
Standard
H2O
Nuclease P1
Phospho-diesterase I
Alkaline phosphatase
Complete buffer
FADU-N
FADU-H
Forward Fit: 0
Reverse Fit: 0
Forward Fit: 960
Reverse Fit: 975
1.851+007
Forward Fit: 542
Reverse Fit: 684
1.142E+005
Forward Fit: 962
Reverse Fit: 976
2.163E+007
m/z

k

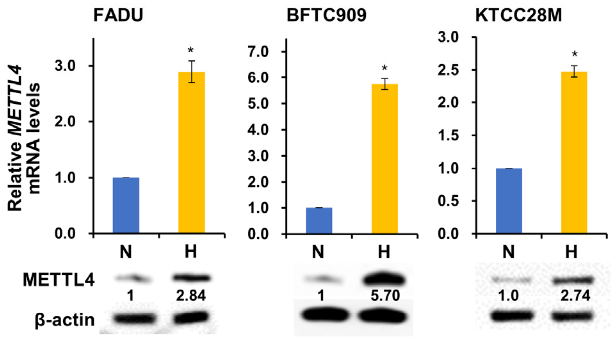
FADU
BFTC909
KTCC28M
Relative METTL4 mRNA levels
N H
METTL4
1 2.84
1 5.70
1.0 2.74
β-actin

l

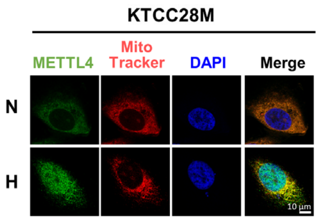
KTCC28M
METTL4
Mito Tracker
DAPI
Merge
N
H
10 µm

m

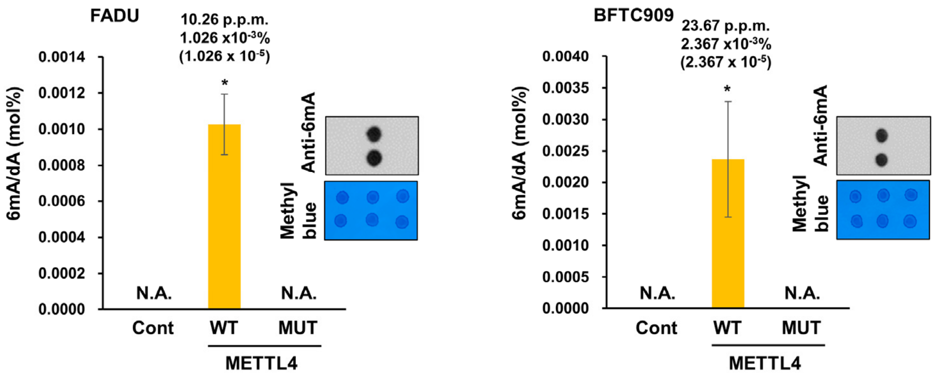
FADU
10.26 p.p.m.
1.026 x10-3%
(1.026 x 10-5)
BFTC909
23.67 p.p.m.
2.367 x10-3%
(2.367 x 10-5)
6mA/dA (mol%)
Anti-6mA
Methyl blue
N.A.
Cont
WT
MUT
METTL4

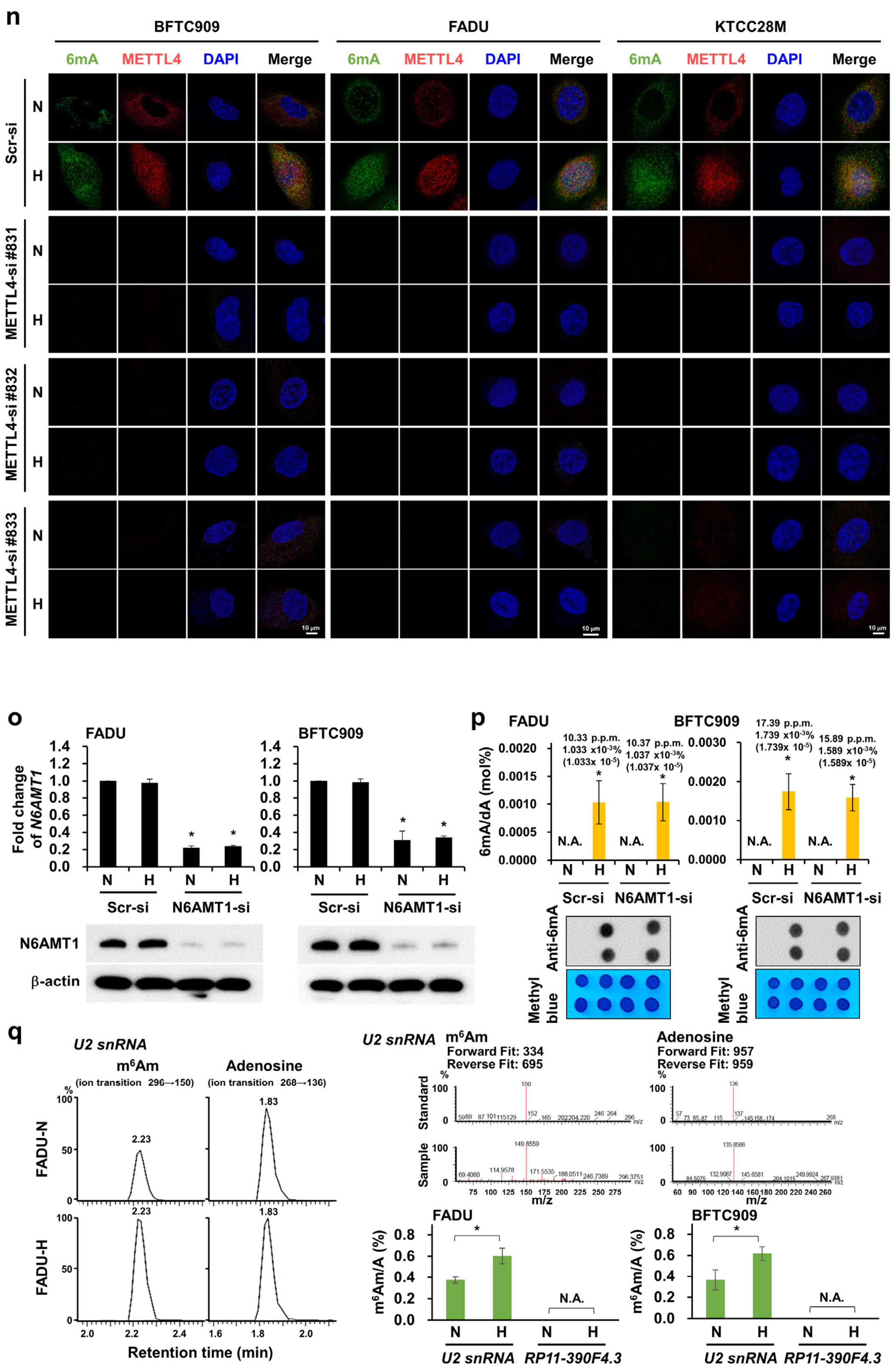
n
BFTC909
FADU
KTCC28M
6mA
METTL4
DAPI
Merge
Scr-si
METTL4-si #831
METTL4-si #832
METTL4-si #833
N
H
10 µm
o
FADU
BFTC909
Fold change of N6AMT1
Scr-si
N6AMT1-si
N6AMT1
β-actin
p
FADU
BFTC909
6mA/dA (mol%)
10.33 p.p.m.
1.033 x10⁻³%
(1.033x 10⁻⁵)
10.37 p.p.m.
1.037 x10⁻³%
(1.037x 10⁻⁵)
17.39 p.p.m.
1.739 x10⁻³%
(1.739x 10⁻⁵)
15.89 p.p.m.
1.589 x10⁻³%
(1.589x 10⁻⁵)
N.A.
Scr-si
N6AMT1-si
Anti-6mA
Methyl blue
q
U2 snRNA
m6Am
(ion transition 296→150)
Adenosine
(ion transition 268→136)
FADU-N
FADU-H
2.23
1.83
Retention time (min)
U2 snRNA m6Am
Forward Fit: 334
Reverse Fit: 695
Adenosine
Forward Fit: 957
Reverse Fit: 959
Standard
Sample
m/z
FADU
BFTC909
m6Am/A (%)
N.A.
U2 snRNA
RP11-390F4.3

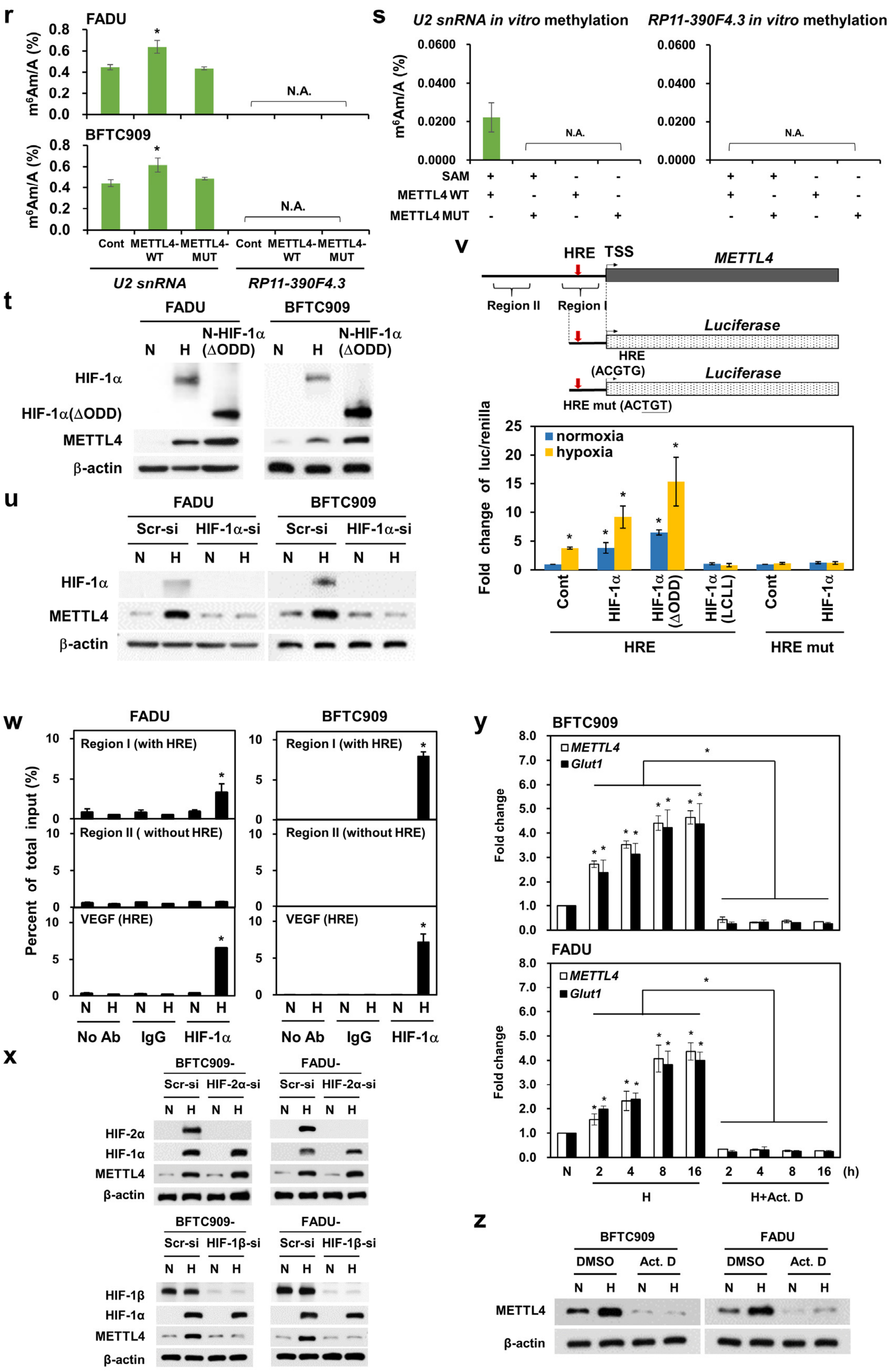

r
FADU
BFTC909
m6Am/A (%)
0.8
0.6
0.4
0.2
0.0
N.A.
Cont
METTL4-WT
METTL4-MUT
U2 snRNA
RP11-390F4.3
s
U2 snRNA in vitro methylation
RP11-390F4.3 in vitro methylation
m6Am/A (%)
0.0600
0.0400
0.0200
0.0000
N.A.
SAM
METTL4 WT
METTL4 MUT
t
FADU
BFTC909
N
H
N-HIF-1α (ΔODD)
HIF-1α
HIF-1α(ΔODD)
METTL4
β-actin
u
Scr-si
HIF-1α-si
v
HRE
TSS
METTL4
Region II
Region I
Luciferase
HRE (ACGTG)
HRE mut (ACTGT)
Fold change of luc/renilla
normoxia
hypoxia
Cont
HIF-1α
HIF-1α (ΔODD)
HIF-1α (LCLL)
HRE
HRE mut
w
Percent of total input (%)
Region I (with HRE)
Region II (without HRE)
Region II (without HRE)
VEGF (HRE)
No Ab
IgG
HIF-1α
x
BFTC909-
FADU-
HIF-2α-si
HIF-1β-si
HIF-2α
HIF-1β
y
METTL4
Glut1
Fold change
N
2
4
8
16
(h)
H
H+Act. D
z
DMSO
Act. D

Figure S1. Induction of 6mA levels and activation of METTL4 by hypoxia/HIF-1α, and other characterizations of m^{6}Am, N6AMT1, HIF-2α, and HIF-1β and Actinomycin experiments.

(a) The relative LC peaks of 6mA and dA in the representative sample were verified by 6mA and dA standards with each MRM transition. The amount of 6mA and dA were determined by UPLC-EIS-MS/MS analysis as a representative chromatogram and quantified by their integrated area in the corresponding chromatogram. Peaks for sample (gDNA digested by FADU cells under hypoxia) and 6mA/dA standard were shown, respectively.

(b) The calibration curves were generated by commercial 6mA and dA standards.

(c) Among the various stress conditions, only hypoxia induced detectable nuclear 6mA levels in FADU and BFTC909 cells by UPLC-ESI-MS/MS analysis (upper panel). The 6mA/dA ratio of each sample was calculated with quantified 6mA and dA values in accordance with the calibration curves obtained from dA and 6mA standards. RNA and protein expression levels of METTL4 after treatment with various stress conditions were shown in the bottom. The asterisk (*) indicated statistical significance ($P<0.05$) between experimental and control group. The non-treated condition was used as a control. Corresponding 6mA dot blots with methyl blue loading controls were shown together with bar graphs.

(d) Hypoxia increased the nuclear 6mA levels of KTCC28M. The 6mA/dA ratios of KTCC28M gDNAs were evaluated from the results of UPLC-ESI-MS/MS quantification. The asterisk (*) indicated statistical significance ($P<0.05$) between experimental and control conditions. N, normoxia; H, hypoxia. Normoxic condition was used as a control. Corresponding 6mA dot blots with methyl blue loading controls were shown together with bar graphs.

(e) The efficiency of removing mitochondrial DNA from the genomic DNA extraction step was quantified by quantitative real-time PCR analysis using mitochondrial and nuclear specific primers in FADU, BFTC909, and KTCC28M cell lines. The quality of the extraction was assessed by comparing the fold change of mitochondrial DNA versus genomic DNA using the ΔC_t method.

(f) No 6mA levels were detectable in all the reagents by UPLC-ESI-MS/MS analysis. N, normoxia; H, hypoxia. Normoxic condition was used as a control. The asterisk (*) indicated statistical significance ($P<0.05$) between experimental and control conditions. Corresponding 6mA dot blots with methyl blue loading controls were shown together with bar graphs.

(g) Immunofluorescence staining showed the consistently increased nuclear and cytoplasmic staining of 6mA in FADU, BFTC909, and KTCC28M cells under hypoxia with or without antigen retrieval procedure. Cell nuclei were stained by DAPI.

Mitochondria were stained by MitoTracker. Bar graph represented the percentage of cells containing nuclear or cytoplasmic 6mA signals. N, normoxia; H, hypoxia. Normoxic condition was used as a control. The asterisk (*) indicated statistical significance ($P<0.05$) between experimental and control conditions. N.S., not statistically significant.

(h) Immunofluorescence staining showed the increased nuclear staining of 6mA in BFTC909 and KTCC28M cells under hypoxia. 6mA signals still remained under RNase treatment, but mostly disappeared under DNase or RNase+DNase treatment, indicating the specific nuclear 6mA signals. Cell nuclei were stained by DAPI. Mitochondria were stained by MitoTracker. N, normoxia; H, hypoxia. Normoxic condition was used as a control.

(i) Bar graph represented the percentage of cells containing nuclear or cytoplasmic 6mA signals. N, normoxia; H, hypoxia. Normoxic condition was used as a control. The asterisk (*) indicated statistical significance ($P<0.05$) between experimental and control conditions.

(j) MS/MS fragmentation profiling confirmed that no 6mA levels were detected in all the reagents tested. Detected 6mA and dA in gDNAs were verified by product ion conformation spectra (PICS) fit to the spectrum generated from each standard. Parental ion of 6mA was m/z 266 with major daughter ion m/z 150. Parental ion of dA was m/z 252 with major daughter ion m/z 136.

(k) Hypoxia increased METTL4 levels (mRNAs and proteins) in FADU, BFTC909, and KTCC28M cell lines. The quantification of METTL4 protein levels induced by hypoxia are listed below the lane. β-actin was used as a loading control. The asterisk (*) indicated statistical significance ($P<0.05$) between experimental and control conditions. N, normoxia; H, hypoxia. Normoxic condition was used as a control.

(l) Immunofluorescence staining showed the increased nuclear METTL4 levels under hypoxia in KTCC28M cells. Cell nuclei were stained by DAPI. Mitochondria were stained by MitoTracker. N, normoxia; H, hypoxia. Normoxic condition was used as a control.

(m) Overexpression of METTL4, but not the enzymatically inactive mutant, increased the 6mA levels in FADU and BFTC909 cell lines by UPLC-ESI-MS/MS analysis. The cell clone transfected with the control vector was used as a control. The asterisk (*) indicated statistical significance ($P<0.05$) between experimental and control groups. Corresponding 6mA dot blots with methyl blue loading controls were shown together with bar graphs.

(n) Immunofluorescence staining showed the staining of METTL4 under hypoxia and abolishment of METTL4 staining under *METTL4* knockdown in BFTC909, FADU, and KTCC28M cell lines. Three different siRNAs were used to knockdown *METTL4*.

Knockdown using the scrambled siRNA under normoxia condition was used as a control. Cell nuclei were stained by DAPI. N, normoxia; H, hypoxia.

(o) Knockdown of *N6AMT1* decreased the *N6AMT1* mRNA and protein levels in FADU and BFTC909 cells by qRT-PCR and Western blot analysis. N, normoxia; H, hypoxia. Knockdown using the scrambled siRNA under normoxia condition was used as a control. The asterisk (*) indicated statistical significance (P<0.05) between experimental and control groups.

(p) Knockdown of *N6AMT1* showed that the induction of 6mA by hypoxia still maintained in the presence of *N6AMT1* knockdown using UPLC-ESI-MS/MS analysis in FADU and BFTC909 cells. N, normoxia; H, hypoxia. Knockdown using the scrambled siRNA under normoxia condition was used as a control. The asterisk (*) indicated statistical significance (P<0.05) between experimental and control groups. Corresponding 6mA dot blots with methyl blue loading controls were shown together with bar graphs.

(q) Hypoxia increased the *U2 snRNA* m6Am levels of FADU and BFTC909 cells. The quantity of m^{6}Am and Adenosine of digested *U2 snRNAs* of FADU cells was determined by UPLC-ESI-MS/MS analysis as the representative chromatograms. The nucleosides of digested *U2 snRNA*s were identified using LC retention with MS/MS spectra and quantified based on the ion mass transitions; m^{6}Am (m/z): 296 to 150 and adenosine (m/z): 268 to 136 in MRM mode. Peaks for normoxia and hypoxia samples of FADU cells were shown. Detected m6Am and A in *U2 snRNA*s were verified by product ion conformation spectra (PICS) fit to the spectrum generated from each standard. Parental ion of m6Am was m/z 296 with major daughter ion m/z 150. Parental ion of A was m/z 268 with major daughter ion m/z 136. The m^{6}Am/A ratios of FADU and BFTC909 RNAs (*U2 snRNA* or *RP11-390F4.3*) were evaluated from the results of UPLC-ESI-MS/MS quantification. N, normoxia; H, hypoxia. Normoxic condition was used as a control. The asterisk (*) indicated statistical significance (P<0.05) between experimental and control condition.

(r) The amount of m^{6}Am and adenosine of *U2 snRNA* and *RP11-390F4.3* digested by FADU and BFTC909 cells were determined by UPLC-ESI-MS/MS analysis. Results showed that mutation of the enzymatic site of METTL4 abolished the *U2 snRNA* m^{6}Am modifying activity in FADU and BFTC909 cells. The cell clone transfected with the control vector was used as a control. The asterisk (*) indicated statistical significance (P<0.05) between experimental and control groups.

(s) *In vitro* RNA methylation assays showed mutation of the enzymatic site of METTL4 abolished the *U2 snRNA* m^{6}Am levels.

(t) Hypoxia and overexpression of a constitutively active HIF-1α(ΔODD) under normoxia activated METTL4 expression. N, normoxia; H, hypoxia.

(u) Knockdown of *HIF-1α* abolished the activation of METTL4 induced by hypoxia by Western blot analysis. N, normoxia; H, hypoxia. Knockdown using the scrambled siRNA was used as a control.
(v) Reporter gene assays showed the identification of HRE (hypoxia response element) in the proximal promoter of *METTL4* and hypoxia/HIF-1α activated the *METTL4* promoter activity. The asterisk (*) indicated statistical significance (P<0.05) between experimental and control groups. The luciferase/renilla activities of FADU cells co-transfected with reporter construct and pcDNA3 control vector under normoxia were used as the baseline control.
(w) Chromatin immunoprecipitation (ChIP) assays showed the direct binding of HIF-1α to the HRE located in *METTL4* promoter under hypoxia in FADU and BFTC909 cells. N, normoxia; H, hypoxia. No antibody/normoxia condition was used as a control. The binding of HIF-1α to the *VEGF* promoter was used as a positive control. The asterisk (*) indicated statistical significance (P<0.05) between experimental and control groups.
(x) Knockdown of *HIF-2α* did not abolish the activation of METTL4 induced by hypoxia by Western blot analysis. Knockdown of *HIF-1β* abolished the activation of METTL4 induced by hypoxia by Western blot analysis. N, normoxia; H, hypoxia. Knockdown using the scrambled siRNA was used as a control.
(y-z) Actinomycin D treatment significantly decreased the activation of METTL4 (at the mRNA and protein levels) under hypoxia. N, normoxia; H, hypoxia. Treatment with dimethyl sulfoxide (DMSO) under normoxic condition was used as a control. The asterisk (*) indicated statistical significance (P<0.05) between experimental and control groups. Actinomycin D (Act. D; 2 μg/ml) in DMSO was used to study transcriptional regulation.
The error bars represented the standard deviation (SD). Student's *t*-test was used to compare two groups of independent samples. For details, see method section.

Figure S2

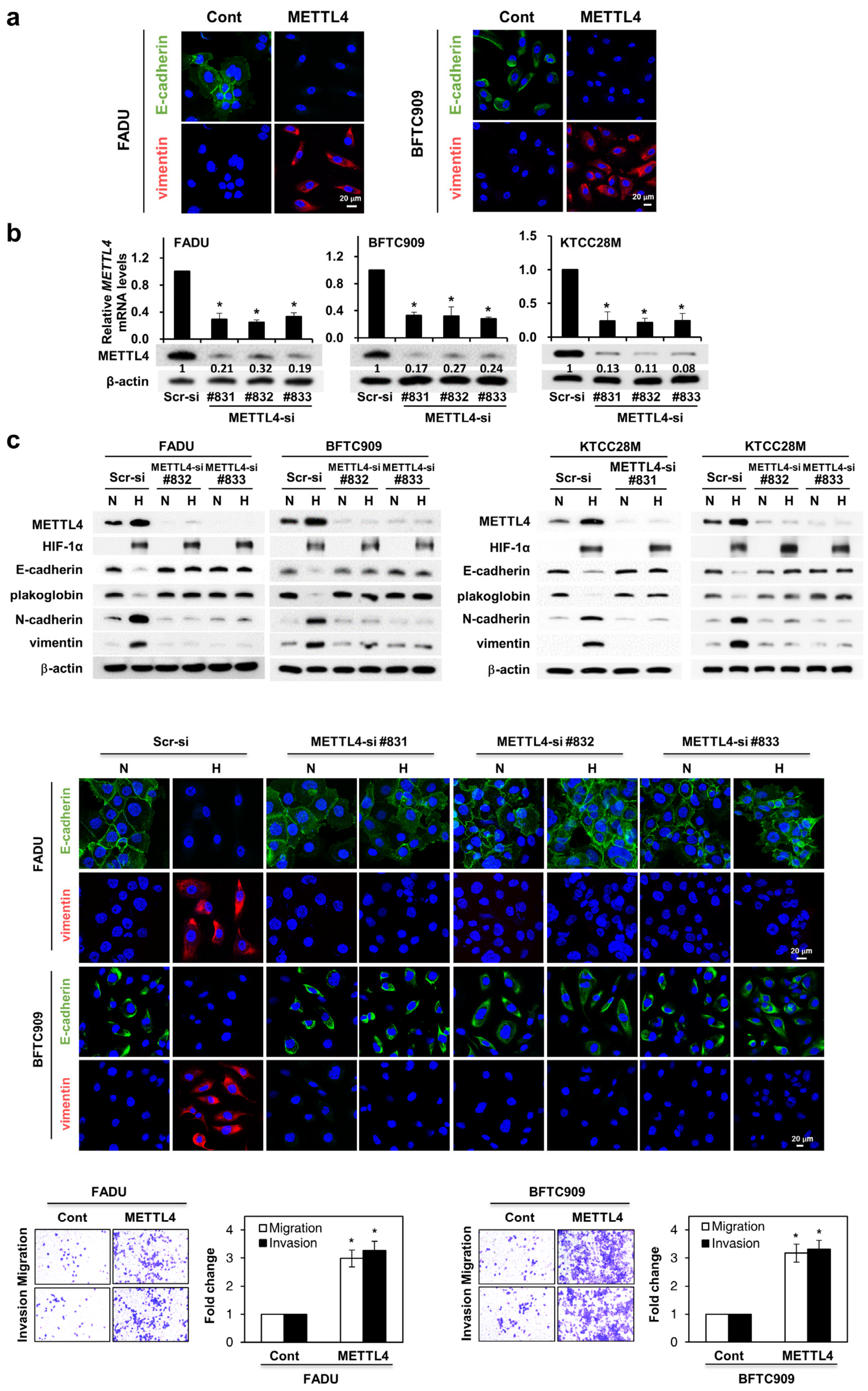

a
Cont
METTL4
FADU
E-cadherin
vimentin
BFTC909
20 µm
b
Relative METTL4 mRNA levels
FADU
BFTC909
KTCC28M
1.2
0.8
0.4
0.0
1.5
1.0
0.5
METTL4
β-actin
1
0.21
0.32
0.19
0.17
0.27
0.24
0.13
0.11
0.08
Scr-si
#831
#832
#833
METTL4-si
c
N
H
METTL4-si #832
METTL4-si #833
METTL4-si #831
HIF-1α
E-cadherin
plakoglobin
N-cadherin
vimentin
d
Scr-si
METTL4-si #831
METTL4-si #832
METTL4-si #833
e
Invasion Migration
Fold change
Migration
Invasion
4
3
2
1
0
*

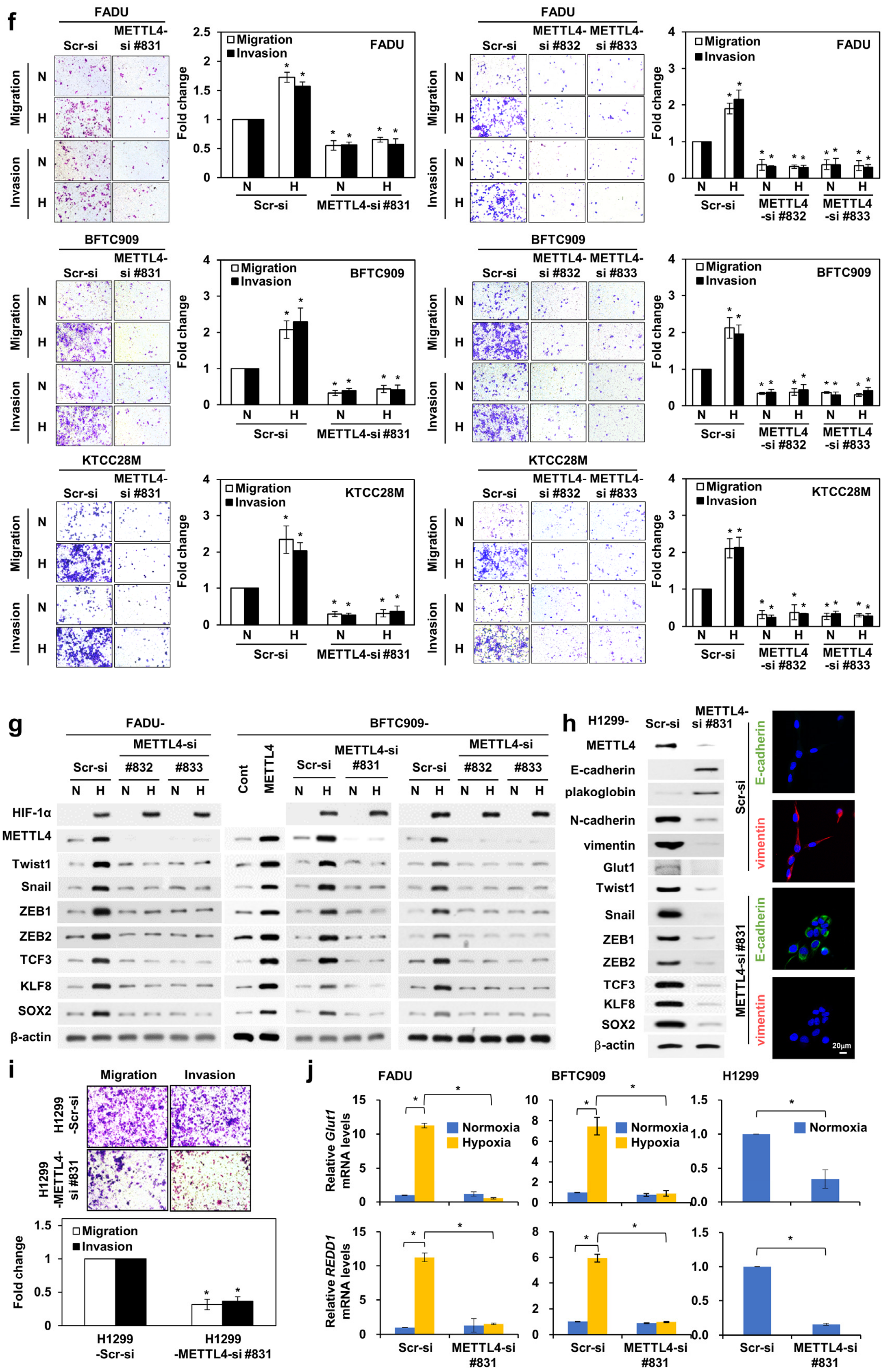
f
FADU
Scr-si
METTL4-si #831
Migration
Invasion
N
H
Fold change
Migration
Invasion
METTL4-si #832
METTL4-si #833
BFTC909
KTCC28M
g
FADU-
BFTC909-
METTL4-si
Cont
METTL4
HIF-1α
METTL4
Twist1
Snail
ZEB1
ZEB2
TCF3
KLF8
SOX2
β-actin
h
H1299-
E-cadherin
plakoglobin
N-cadherin
vimentin
Glut1
20μm
i
H1299 -Scr-si
H1299 -METTL4-si #831
j
Relative Glut1 mRNA levels
Relative REDD1 mRNA levels
Normoxia
Hypoxia
H1299

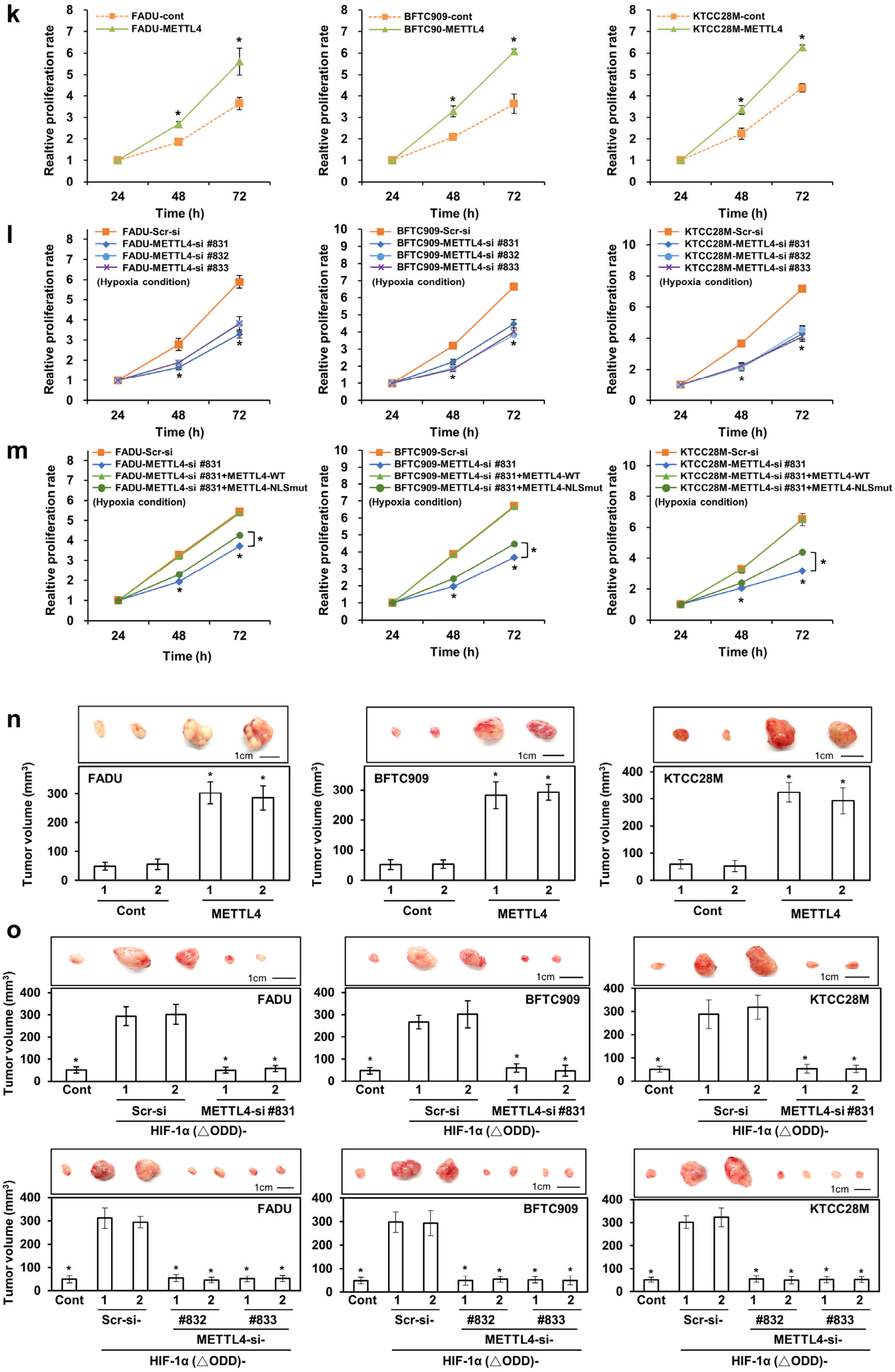
k
FADU-cont
FADU-METTL4
BFTC909-cont
BFTC90-METTL4
KTCC28M-cont
KTCC28M-METTL4
Realtive proliferation rate
Time (h)
l
FADU-Scr-si
FADU-METTL4-si #831
FADU-METTL4-si #832
FADU-METTL4-si #833
BFTC909-Scr-si
BFTC909-METTL4-si #831
BFTC909-METTL4-si #832
BFTC909-METTL4-si #833
KTCC28M-Scr-si
KTCC28M-METTL4-si #831
KTCC28M-METTL4-si #832
KTCC28M-METTL4-si #833
(Hypoxia condition)
m
FADU-Scr-si
FADU-METTL4-si #831
FADU-METTL4-si #831+METTL4-WT
FADU-METTL4-si #831+METTL4-NLSmut
BFTC909-Scr-si
BFTC909-METTL4-si #831
BFTC909-METTL4-si #831+METTL4-WT
BFTC909-METTL4-si #831+METTL4-NLSmut
KTCC28M-Scr-si
KTCC28M-METTL4-si #831
KTCC28M-METTL4-si #831+METTL4-WT
KTCC28M-METTL4-si #831+METTL4-NLSmut
(Hypoxia condition)
n
1cm
FADU
BFTC909
KTCC28M
Tumor volume (mm³)
Cont
METTL4
o
Scr-si
METTL4-si #831
HIF-1α (△ODD)-
Scr-si-
#832
#833
METTL4-si-

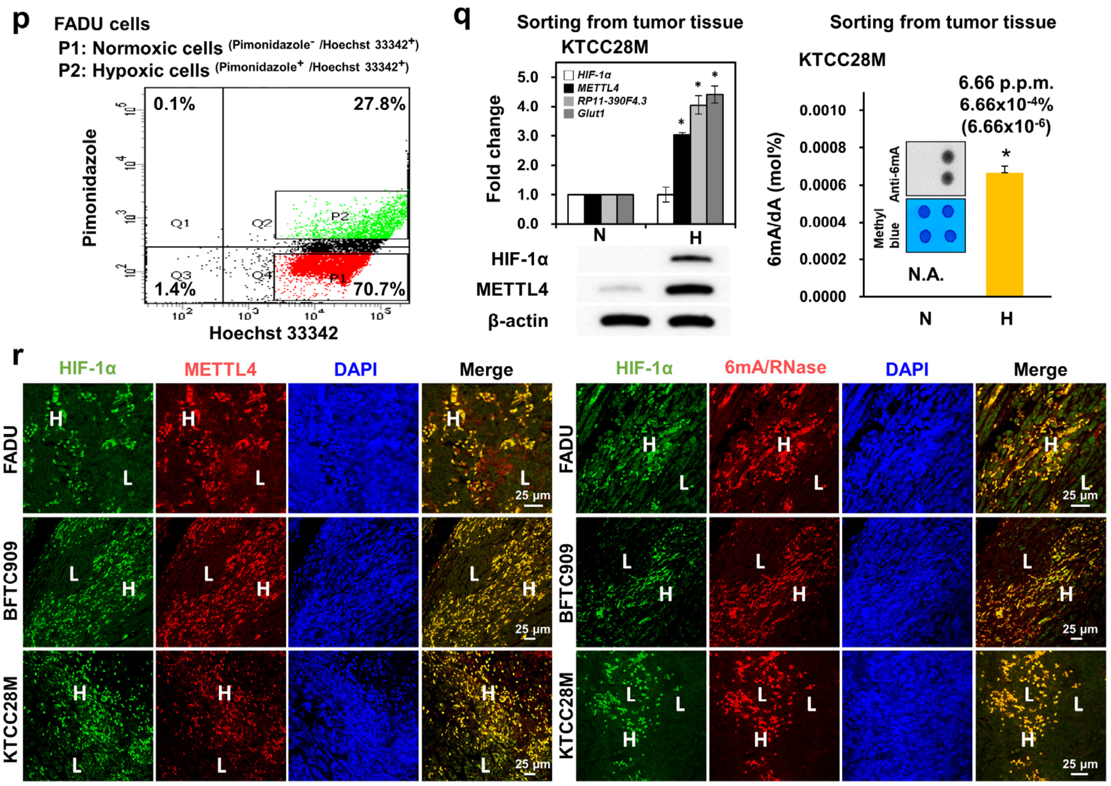
p
FADU cells
P1: Normoxic cells (Pimonidazole- /Hoechst 33342+)
P2: Hypoxic cells (Pimonidazole+ /Hoechst 33342+)
Pimonidazole
Hoechst 33342
0.1%
27.8%
1.4%
70.7%
Q1
Q2
Q3
Q4
P1
P2
q
Sorting from tumor tissue
KTCC28M
Fold change
HIF-1α
METTL4
RP11-390F4.3
Glut1
N
H
HIF-1α
METTL4
β-actin
Sorting from tumor tissue
KTCC28M
6mA/dA (mol%)
6.66 p.p.m.
6.66x10-4%
(6.66x10-6)
Anti-6mA
Methyl blue
N.A.
N
H
r
HIF-1α
METTL4
DAPI
Merge
HIF-1α
6mA/RNase
DAPI
Merge
FADU
BFTC909
KTCC28M
H
L
25 µm

Figure S2. The critical role of METTL4 in inducing EMT and metastasis, induction of tumorigenicity and *in vitro* migration/invasion activity by METTL4, the critical role of METTL4 in EMT and *in vitro* migration/invasion activity in two other cell lines, the set of EMT regulators activated by hypoxia, analysis of xenografted tumor samples after sorting by real-time PCR analysis and UPLC-ESI-MS/MS measurement of 6mA levels, the essential role of enzymatic activity of METTL4 in hypoxia-induced EMT, and increased expression of METTL4, 6mA and HIF-1α in the tumor samples of UTUC patients.

(a) Overexpression of METTL4 induced EMT by immunofluorescence staining of E-cadherin and vimentin. Green, E-cadherin; red, vimentin. Cell nuclei were stained by DAPI. The cell clone transfected with the control vector was used as a control.

(b) Knockdown of *METTL4* decreased the METTL4 mRNA and protein levels in FADU, BFTC909, and KTCC28M cells by qRT-PCR and Western blot analysis. Three different siRNAs were used to knockdown *METTL4*. The quantification of METTL4 protein levels after *METTL4* knockdown are listed below the lane. β-actin was used as a loading control. Knockdown using the scrambled siRNA was used as a control. The asterisk (*) indicated statistical significance ($P<0.05$) between experimental and control groups.

(c-d) Western blot analysis and immunofluorescence staining showed that knockdown of *METTL4* abolished the hypoxia-induced EMT in different cell lines. Three different siRNAs were used to knockdown *METTL4*. Knockdown using the scrambled siRNA was used as a control. N, normoxia; H, hypoxia. Cell nuclei were stained by DAPI. Green, E-cadherin; red, vimentin.

(e) METTL4 overexpression induced the *in vitro* migration and invasion activity of two different cell lines. Representative photos of the *in vitro* migration and invasion activity were shown in this panel. The cell clone transfected with the control vector was used as a control. The asterisk (*) indicated statistical significance ($P<0.05$) between experimental and control groups.

(f) Knockdown of *METTL4* significantly decreased the *in vitro* migration and invasion activity of three different cell lines. Representative photos of the *in vitro* migration and invasion activity were shown in this panel. Three different siRNAs were used to knockdown *METTL4*. Knockdown using the scrambled siRNA under normoxic condition was used as a control. The asterisk (*) indicated statistical significance ($P<0.05$) between experimental and control conditions. N, normoxia; H, hypoxia.

(g) METTL4 overexpression activated the expression of EMT regulator genes in BFTC909 cells and *METTL4* knockdown abolished the activation of EMT regulator gene expression induced by hypoxia in FADU and BFTC909 cells by Western blot analysis. Three different siRNAs were used to knockdown *METTL4*. Knockdown using

the scrambled siRNA was used as a control. N, normoxia; H, hypoxia.

(h) Knockdown of *METTL4* reversed the mesenchymal phenotypes of H1299 cells by Western blot and immunofluorescence staining experiments. Knockdown using the scrambled siRNA was used as a control. Cell nuclei were stained by DAPI. Green, E-cadherin; red, vimentin.

(i) Knockdown of *METTL4* significantly decreased the *in vitro* migration and invasion activity of H1299 cells. Representative photos of the *in vitro* migration and invasion activity were shown on the top. Knockdown using the scrambled siRNA was used as a control. The asterisk (*) indicated statistical significance (P<0.05) between experimental and control groups.

(j) Knockdown of *METTL4* decreased the RNA levels of *Glut1* and *REDD1*genes in three different cell lines. The measurement was assayed under normoxia vs. hypoxia in the FADU and BFTC909 cells (epithelial phenotype cells) and under normoxic condition in H1299 cells (mesenchymal phenotype cells). Measurement of *Glut1* (upper panel) and *REDD1* (lower panel) gene expression by real-time PCR analysis was performed. Knockdown using the scrambled siRNA under normoxic condition was used as a control for all cell lines. The asterisk (*) indicated statistical significance (P<0.05) between experimental and control groups.

(k) Overexpression of METTL4 increased the cell proliferation rates of FADU, BFTC909, and KTCC28M cells. The cell clone transfected with the control vector was used as a control. The asterisk (*) indicated statistical significance (P<0.05) between experimental and control groups.

(l) Knockdown of *METTL4* significantly decreased the cell proliferation rates of three different cell lines under hypoxia. Knockdown using the scrambled siRNA under hypoxia condition was used as a control. The asterisk (*) indicated statistical significance (P<0.05) between experimental and control groups.

(m) *METTL4* knockdown followed by rescue experiments showed that the METTL4-NLSmut only partially rescued the cell proliferation rates that were decreased by *METTL4* knockdown under hypoxia compared with the full rescue by METTL4 wild type in FADU, BFTC909, and KTCC28M cells. Knockdown using the scrambled siRNA under hypoxia condition was used as a control. The asterisk (*) indicated statistical significance (P<0.05) between experimental and control groups.

(n) Overexpression of METTL4 increased the tumor volume of FADU, BFTC909, and KTCC28M cells using xenograft implantation assays. The cell clone transfected with the control vector was used as a control. The asterisk (*) indicated statistical significance (P<0.05) between experimental and control groups.

(o) HIF-1α-overexpression induced tumor volume increase in three different cell lines, and the HIF-1α-induced increase in tumor volume was abolished by knockdown of

METTL4 using three different siRNA vectors. Knockdown using the scrambled siRNA in overexpressing a constitutively active HIF-1α(ΔODD) cells were used as a control. The asterisk (*) indicated statistical significance (P<0.05) between experimental and control groups.

(p) Hypoxic tumor cells sorted from xenografted tumors from FADU cells were shown by FACSCalibur instrument. Two-color staining with pimonidazole and Hoechst 33342 as the representative chromatograms. The hypoxic cell population was defined as Hoechst 33342+ and pimonidazole+ cells (Area P2), and the normoxic cell population was defined as Hoechst 33342+ and pimonidazole- cells (Area P1).

(q) Hypoxic tumor cells sorted from xenografted tumors from KTCC28M cells showed the increased HIF-1α and METTL4 protein levels together with increased HIF-1α target gene expression (*METTL4, RP11-390F4.3,* and *Glut1* expression were measured; left panel). Hypoxic tumor cells sorted from xenografted tumors from KTCC28M cells showed an increase in the 6mA levels by UPLC-ESI-MS/MS measurement. *Glut1* activation was used as a positive control. N, normoxia; H, hypoxia. Normoxic condition was used as a control. The asterisk (*) indicated statistical significance (P<0.05) between experimental and control groups. Corresponding 6mA dot blots with methyl blue loading controls were shown together with bar graphs.

(r) Immunofluorescence staining showed the co-localization of HIF-1α, METTL4, and 6mA in three different cell lines cells. Green fluorescence represented staining of HIF-1α; red fluorescence represented staining of METTL4 or 6mA. Cell nuclei were stained by DAPI. H, highly colocalized area; L, less colocalized area. Notably, HIF-1α signal was highly colocalized with METTL4 or 6mA signal.

The error bars represented the standard deviation (SD). Student's *t*-test was used to compare two groups of independent samples. For details, see method section.

Figure S3

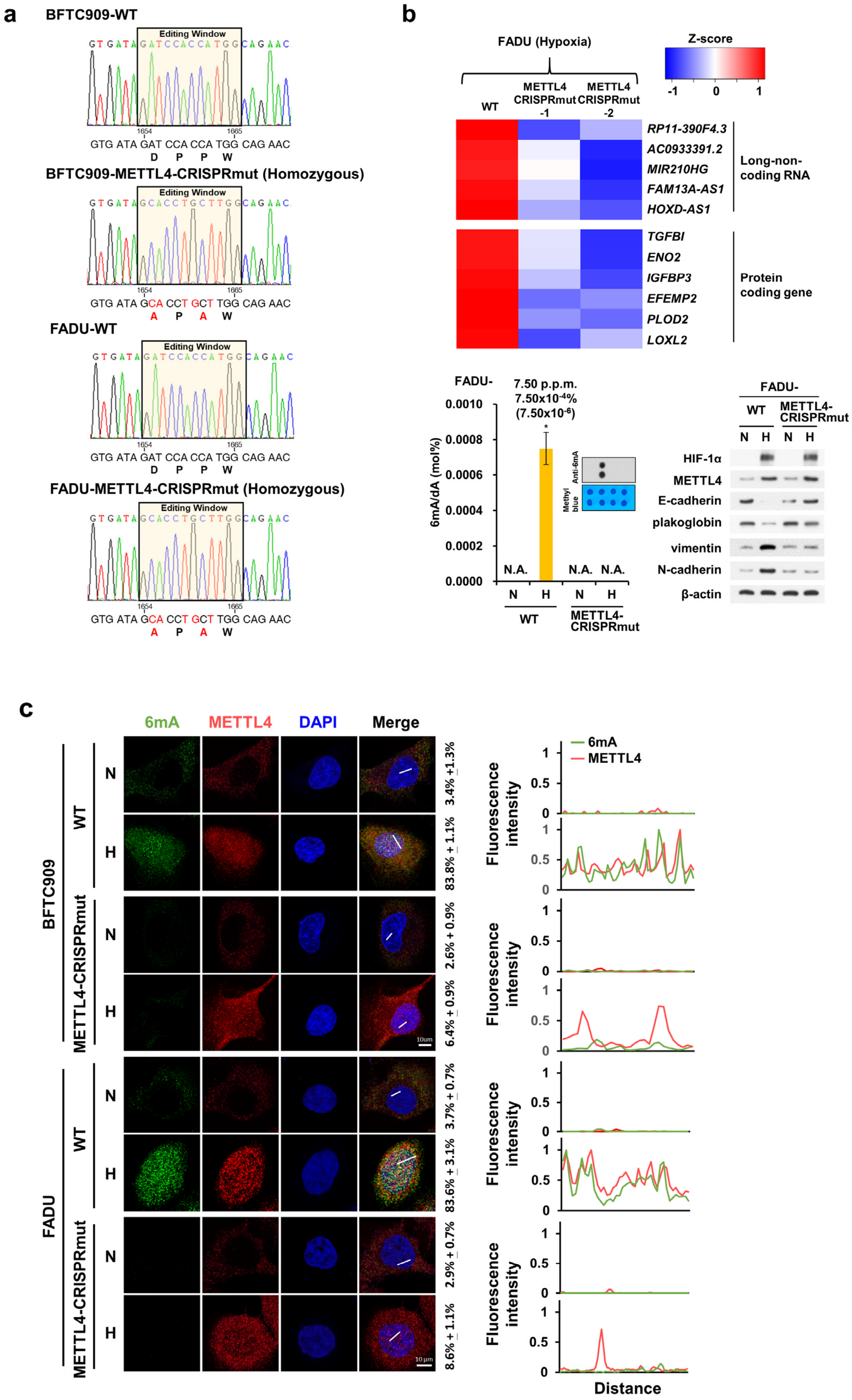
a
BFTC909-WT
Editing Window
GTG ATA GAT CCA CCA TGG CAG AAC
D P P W
BFTC909-METTL4-CRISPRmut (Homozygous)
GTG ATA GCA CCT GCT TGG CAG AAC
A P A W
FADU-WT
GTG ATA GAT CCA CCA TGG CAG AAC
D P P W
FADU-METTL4-CRISPRmut (Homozygous)
GTG ATA GCA CCT GCT TGG CAG AAC
A P A W
b
FADU (Hypoxia)
WT
METTL4 CRISPRmut -1
METTL4 CRISPRmut -2
Z-score
-1 0 1
RP11-390F4.3
AC0933391.2
MIR210HG
FAM13A-AS1
HOXD-AS1
Long-non-coding RNA
TGFBI
ENO2
IGFBP3
EFEMP2
PLOD2
LOXL2
Protein coding gene
FADU-
7.50 p.p.m.
7.50x10-4%
(7.50x10-6)
6mA/dA (mol%)
N.A.
N.A. N.A.
N H N H
WT
METTL4-CRISPRmut
Anti-6mA
Methyl blue
FADU-
WT
METTL4-CRISPRmut
N H N H
HIF-1α
METTL4
E-cadherin
plakoglobin
vimentin
N-cadherin
β-actin
c
6mA METTL4 DAPI Merge
BFTC909
FADU
WT
METTL4-CRISPRmut
N
H
3.4% + 1.3%
83.8% + 1.1%
2.6% + 0.9%
6.4% + 0.9%
3.7% + 0.7%
83.6% + 3.1%
2.9% + 0.7%
8.6% + 1.1%
6mA
METTL4
Fluorescence intensity
Distance

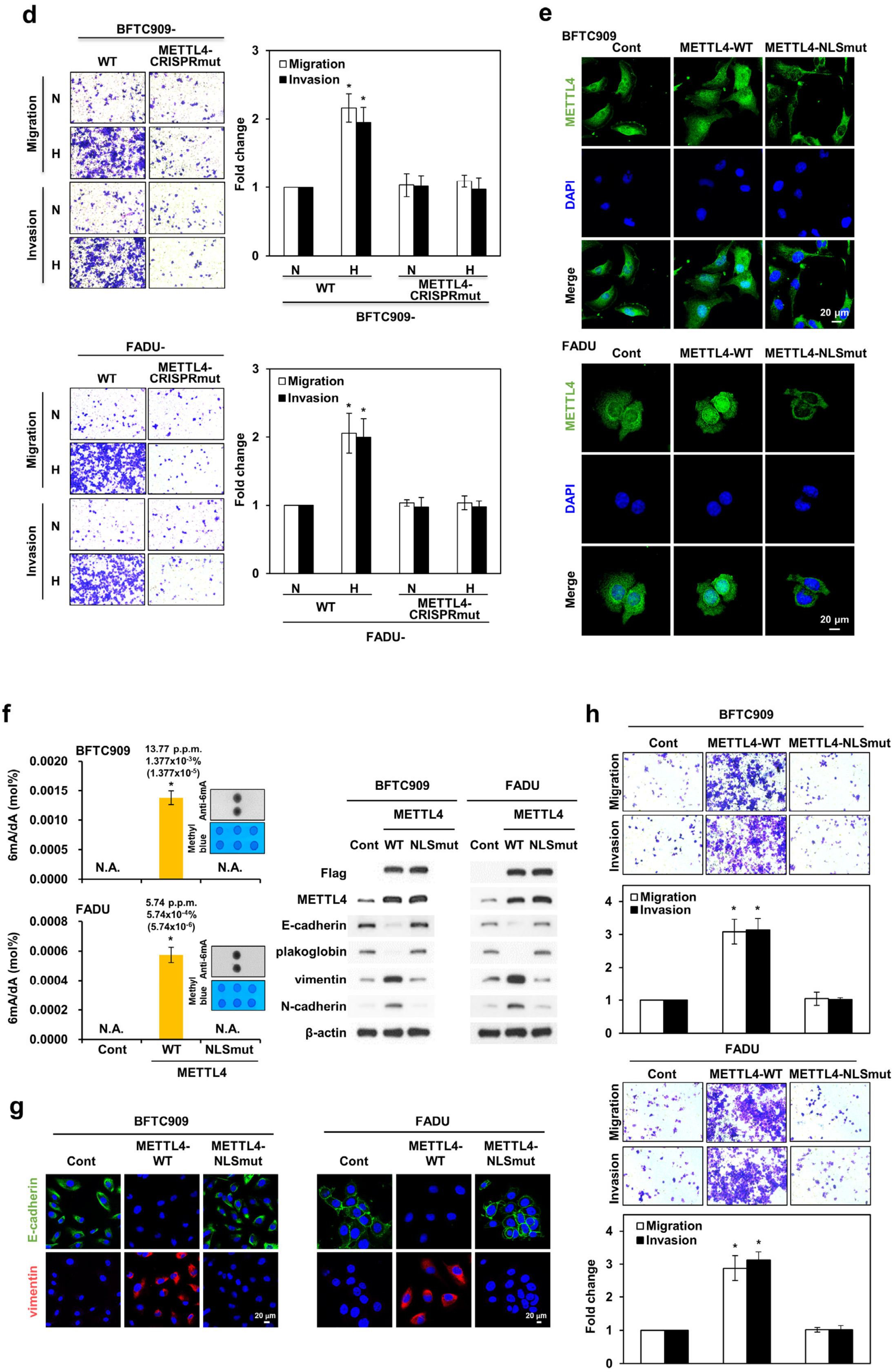
d
BFTC909-
WT
METTL4-CRISPRmut
Migration
Invasion
N
H
Fold change
Migration
Invasion
FADU-
e
BFTC909
FADU
Cont
METTL4-WT
METTL4-NLSmut
METTL4
DAPI
Merge
20 μm
f
6mA/dA (mol%)
13.77 p.p.m.
1.377x10-3%
(1.377x10-5)
5.74 p.p.m.
5.74x10-4%
(5.74x10-6)
Anti-6mA
Methyl blue
N.A.
Cont
WT
NLSmut
METTL4
Flag
E-cadherin
plakoglobin
vimentin
N-cadherin
β-actin
g
E-cadherin
vimentin
METTL4-WT
METTL4-NLSmut
h

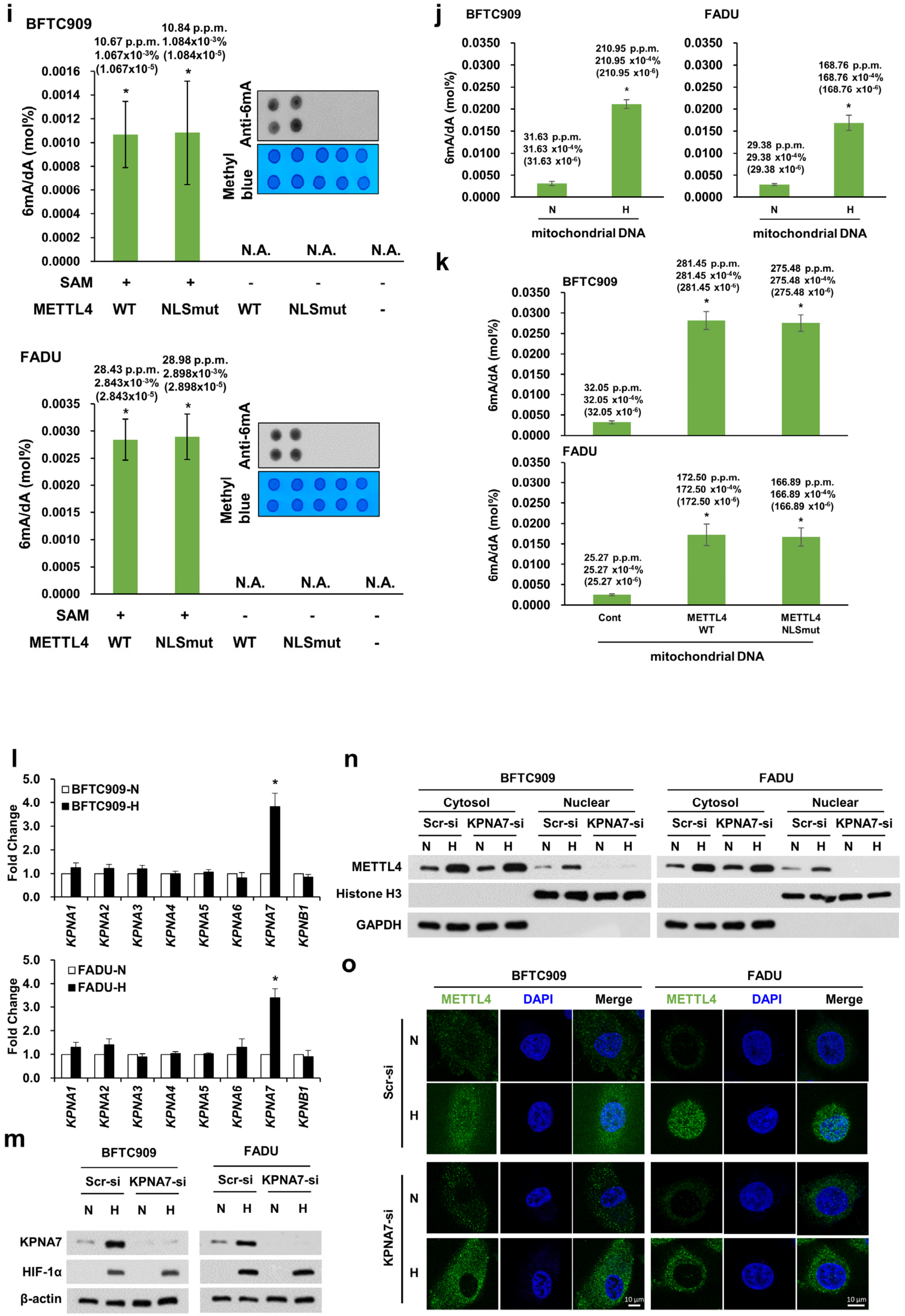

i
BFTC909
10.67 p.p.m. 1.067x10-3% (1.067x10-5)
10.84 p.p.m. 1.084x10-3% (1.084x10-5)
6mA/dA (mol%)
Anti-6mA
Methyl blue
N.A.
SAM
METTL4
WT
NLSmut
FADU
28.43 p.p.m. 2.843x10-3% (2.843x10-5)
28.98 p.p.m. 2.898x10-3% (2.898x10-5)
j
BFTC909
FADU
210.95 p.p.m. 210.95 x10-4% (210.95 x10-6)
31.63 p.p.m. 31.63 x10-4% (31.63 x10-6)
168.76 p.p.m. 168.76 x10-4% (168.76 x10-6)
29.38 p.p.m. 29.38 x10-4% (29.38 x10-6)
mitochondrial DNA
k
281.45 p.p.m. 281.45 x10-4% (281.45 x10-6)
275.48 p.p.m. 275.48 x10-4% (275.48 x10-6)
32.05 p.p.m. 32.05 x10-4% (32.05 x10-6)
172.50 p.p.m. 172.50 x10-4% (172.50 x10-6)
166.89 p.p.m. 166.89 x10-4% (166.89 x10-6)
25.27 p.p.m. 25.27 x10-4% (25.27 x10-6)
Cont
METTL4 WT
METTL4 NLSmut
l
BFTC909-N
BFTC909-H
FADU-N
FADU-H
Fold Change
KPNA1 KPNA2 KPNA3 KPNA4 KPNA5 KPNA6 KPNA7 KPNB1
m
Scr-si
KPNA7-si
KPNA7
HIF-1α
β-actin
n
Cytosol
Nuclear
METTL4
Histone H3
GAPDH
o
METTL4
DAPI
Merge
10 µm

Figure S3. Testing of the enzymatic activity of METTL4 in its ability to induce gene expression and induction of EMT phenotypes, examination of various activities of nuclear localization mutant of METTL4, and identification of KPNA7 as a nuclear transporter of METTL4.

(a) Sequencing results showed the prime-editing CRISPR-Cas9 approach to mutate the enzymatic site (DPPW changed to APAW) of endogenous METTL4 in BFTC909 and FADU cells. The METTL4 wild-type cells were used as controls.

(b) Mutation of the enzymatic site of endogenous METTL4 by a prime-cutting CRISPR-Cas9 approach in FADU cells abolished the induction of hypoxia-regulated gene expression, 6mA levels, and EMT phenotypes by RNA-seq, UPLC-ESI-MS/MS, and Western blot analysis. Corresponding 6mA dot blots with methyl blue loading controls were shown together with bar graphs. N, normoxia; H, hypoxia. The normoxic condition for METTL4 wild-type cells was used as a control. The asterisk (*) indicated statistical significance (P<0.05) between experimental and control groups.

(c) Immunofluorescence staining showed the co-localizations of 6mA and METTL4 induced by hypoxia in BFTC909 and FADU cells. The cells with endogenous mutation of METTL4 enzymatic site did not mediate 6mA deposition and no co-localizations of 6mA and METTL4 could be shown. Cell nuclei were stained by DAPI. Lines indicate the plot profiles shown to the right. Lines were only placed inside nucleus. The percentage and fluorescence intensity of 6mA and METTL4 co-localized signals in the nucleus were shown next to the immunofluorescence images.

(d) Mutation of the enzymatic site of endogenous METTL4 by a prime-cutting CRISPR-Cas9 approach in FADU and BFTC909 cells abolished the induction of the *in vitro* migration and invasion activity by hypoxia. Representative photos of the *in vitro* migration and invasion activity were shown in the panel.

(e) Immunofluorescence staining showed overexpressiion of METTL4-NLSmut could not increase nuclear levels of METTL4 in FADU and BFTC909 cells. Cell nuclei were stained by DAPI. The cell clone transfected with the control vector was used as a control.

(f) Overexpression of METTL4, but not METTL4-NLSmut, increased 6mA levels and induced EMT phenotypes in two different cell lines. The cell clone transfected with the control vector was used as a control. The asterisk (*) indicated statistical significance (P<0.05) between experimental and control groups. Corresponding 6mA dot blots with methyl blue loading controls were shown together with bar graphs.

(g) Immunofluorescence staining showed the inability of a nuclear-localization defective METTL4 mutant (METTL4-NLSmut) to induce EMT in the BFTC909 and FADU cells. Green fluorescence represented staining of E-cadherin; red fluorescence represented staining of vimentin. Cell nuclei were stained by DAPI. The cell clone transfected with the control vector was used as a control.

(h) Overexpression of METTL4, but not METTL4-NLSmut, induced EMT phenotypes in two different cell lines. Representative photos of the *in vitro* migration and invasion activity were shown in this panel. The cell clone transfected with the control vector was used as a control. The asterisk (*) indicated statistical significance (P<0.05) between experimental and control groups.
(i) *In vitro* DNA methylation assays showed an increase in the 6mA levels by incubating METTL4 (wild type or NLS mutant) with genomic DNAs from FADU or BFTC909 cells. Either using the METTL4-NLSmut or not incubating with SAM did not induce any measurable 6mA levels. The asterisk (*) indicated statistical significance (P<0.05) between experimental and control groups. Corresponding 6mA dot blots with methyl blue loading controls were shown together with bar graphs.
(j-k) Hypoxia or overexpression of either METTL4-WT or METTL4-NLSmut increased 6mA levels of mitochondrial DNA in two different cell lines by UPLC-ESI-MS/MS analysis. N, normoxia; H, hypoxia. Normoxic condition and the cell clone transfected with the control vector were used as a control. The asterisk (*) indicated statistical significance (P<0.05) between experimental and control groups.
(l) Hypoxia induced the *KPNA7* mRNA levels by real-time PCR analysis in BFTC909 and FADU cells. N, normoxia; H, hypoxia. Normoxic condition was used as a control. The asterisk (*) indicated statistical significance (P<0.05) between experimental and control groups.
(m) Knockdown of *KPNA7* abolished the KPNA7 levels induced by hypoxia in BFTC909 and FADU cells by Western blot analysis. N, normoxia; H, hypoxia. Knockdown using the scrambled siRNA was used as a control.
(n-o) Knockdown of *KPNA7* abolished the nuclear translocation of METTL4 induced by hypoxia by nuclear fractionation (Western blot) analysis and immunofluorescence staining. N, normoxia; H, hypoxia. Knockdown using the scrambled siRNA was used as a control. Histone H3 and GAPDH was used as a nuclear and cytoplasmic marker, respectively. Cell nuclei were stained by DAPI.
The error bars represented the standard deviation (SD). Student's *t*-test was used to compare two groups of independent samples. For details, see method section.

Figure S4

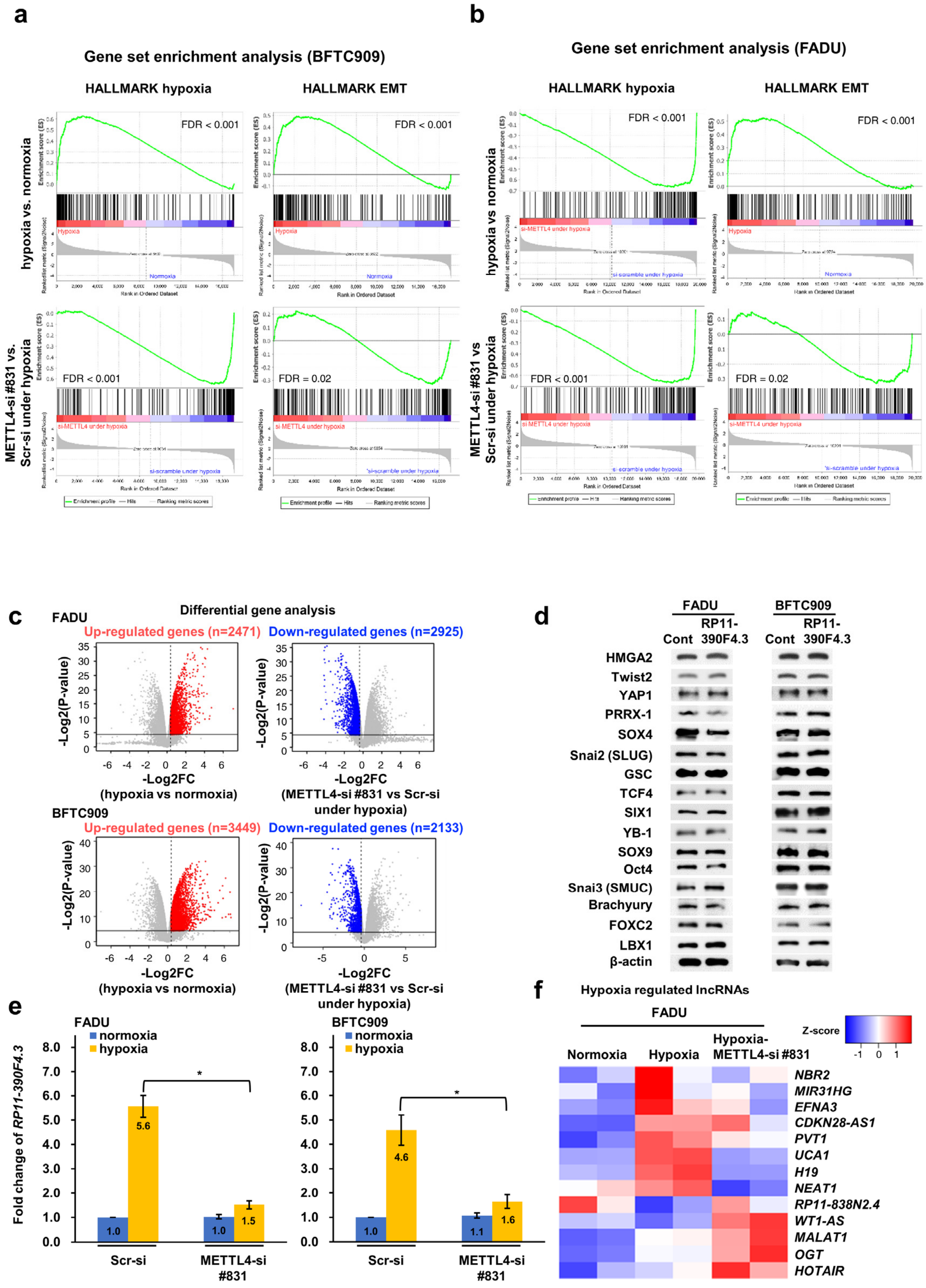
a
Gene set enrichment analysis (BFTC909)
HALLMARK hypoxia
HALLMARK EMT
hypoxia vs. normoxia
METTL4-si #831 vs. Scr-si under hypoxia
FDR < 0.001
FDR = 0.02
b
Gene set enrichment analysis (FADU)
HALLMARK hypoxia
HALLMARK EMT
hypoxia vs normoxia
METTL4-si #831 vs Scr-si under hypoxia
FDR < 0.001
FDR = 0.02
c
FADU
Differential gene analysis
Up-regulated genes (n=2471)
Down-regulated genes (n=2925)
-Log2(P-value)
-Log2FC
(hypoxia vs normoxia)
(METTL4-si #831 vs Scr-si under hypoxia)
BFTC909
Up-regulated genes (n=3449)
Down-regulated genes (n=2133)
d
FADU
BFTC909
Cont
RP11-390F4.3
HMGA2
Twist2
YAP1
PRRX-1
SOX4
Snai2 (SLUG)
GSC
TCF4
SIX1
YB-1
SOX9
Oct4
Snai3 (SMUC)
Brachyury
FOXC2
LBX1
β-actin
e
FADU
BFTC909
normoxia
hypoxia
Fold change of RP11-390F4.3
Scr-si
METTL4-si #831
*
f
Hypoxia regulated lncRNAs
FADU
Normoxia
Hypoxia
Hypoxia-METTL4-si #831
Z-score
NBR2
MIR31HG
EFNA3
CDKN28-AS1
PVT1
UCA1
H19
NEAT1
RP11-838N2.4
WT1-AS
MALAT1
OGT
HOTAIR

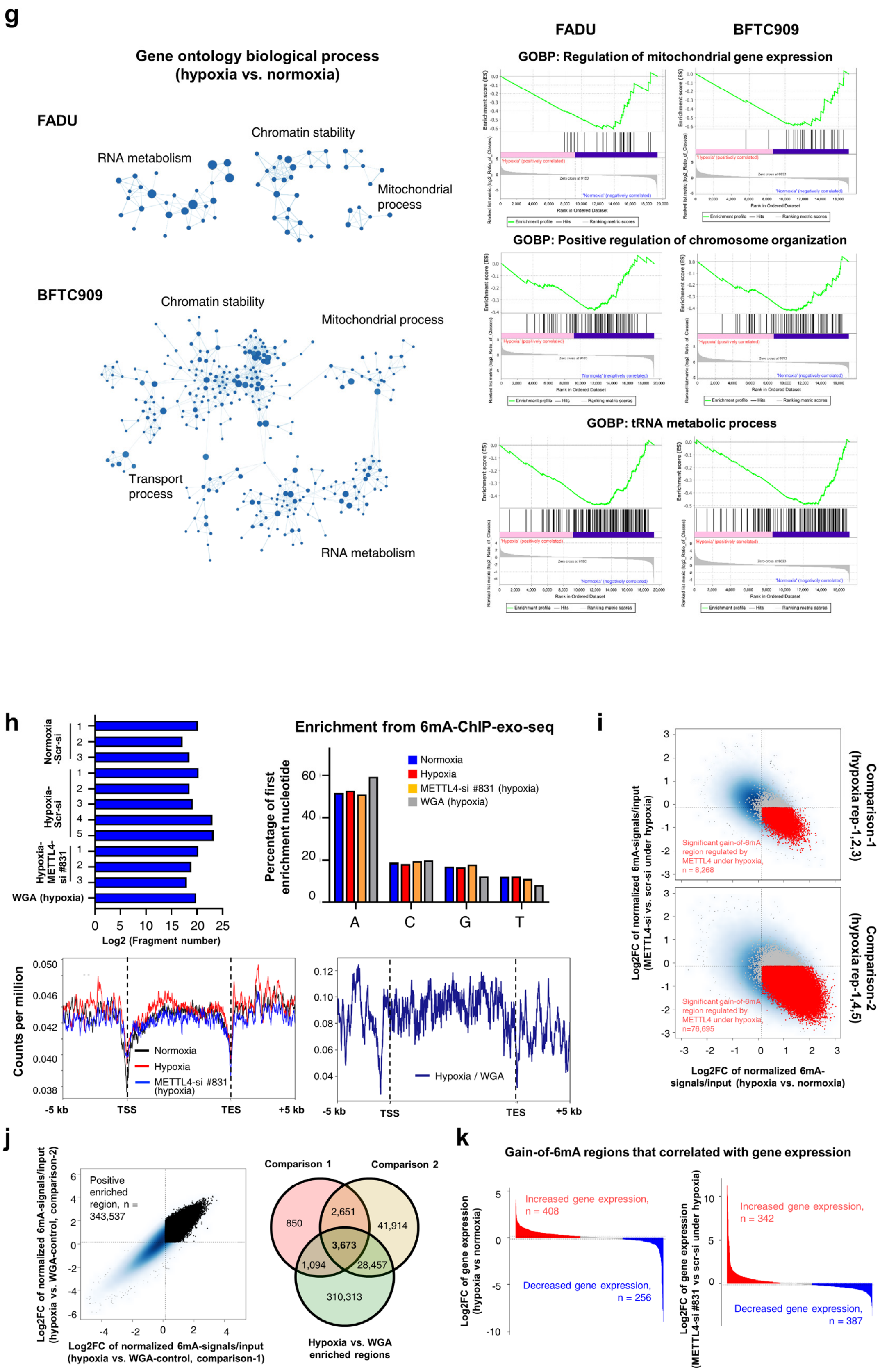
g
Gene ontology biological process
(hypoxia vs. normoxia)
FADU
RNA metabolism
Chromatin stability
Mitochondrial process
BFTC909
Chromatin stability
Mitochondrial process
Transport process
RNA metabolism
FADU
BFTC909
GOBP: Regulation of mitochondrial gene expression
GOBP: Positive regulation of chromosome organization
GOBP: tRNA metabolic process
h
Normoxia -Scr-si
Hypoxia-Scr-si
Hypoxia-METTL4-si #831
WGA (hypoxia)
Log2 (Fragment number)
Enrichment from 6mA-ChIP-exo-seq
Percentage of first enrichment nucleotide
Normoxia
Hypoxia
METTL4-si #831 (hypoxia)
WGA (hypoxia)
A
C
G
T
Counts per million
Normoxia
Hypoxia
METTL4-si #831 (hypoxia)
-5 kb
TSS
TES
+5 kb
Hypoxia / WGA
i
Log2FC of normalized 6mA-signals/input (METTL4-si vs. scr-si under hypoxia)
Comparison-1 (hypoxia rep-1,2,3)
Significant gain-of-6mA region regulated by METTL4 under hypoxia, n = 8,268
Comparison-2 (hypoxia rep-1,4,5)
Significant gain-of-6mA region regulated by METTL4 under hypoxia, n=76,695
Log2FC of normalized 6mA-signals/input (hypoxia vs. normoxia)
j
Log2FC of normalized 6mA-signals/input (hypoxia vs. WGA-control, comparison-2)
Positive enriched region, n = 343,537
Log2FC of normalized 6mA-signals/input (hypoxia vs. WGA-control, comparison-1)
Comparison 1
Comparison 2
850
2,651
41,914
3,673
1,094
28,457
310,313
Hypoxia vs. WGA enriched regions
k
Gain-of-6mA regions that correlated with gene expression
Log2FC of gene expression (hypoxia vs normoxia)
Increased gene expression, n = 408
Decreased gene expression, n = 256
Log2FC of gene expression (METTL4-si #831 vs scr-si under hypoxia)
Increased gene expression, n = 342
Decreased gene expression, n = 387

l

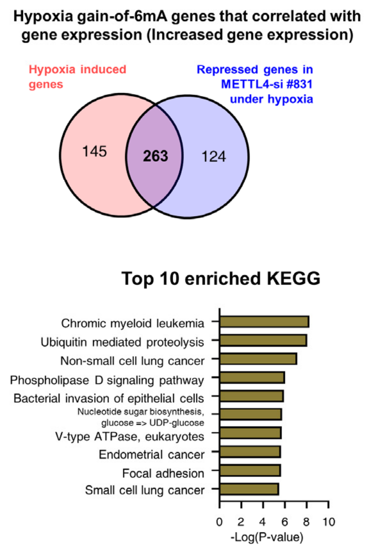

m

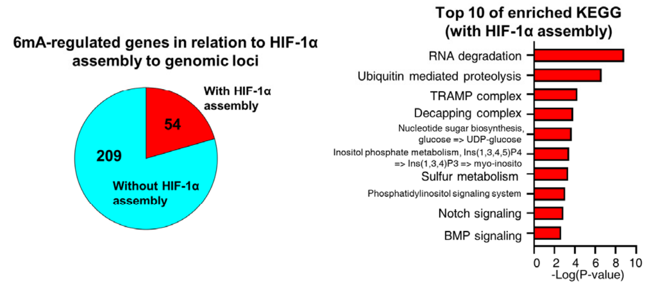

**Angiogenesis**	*AKT3,DAB2IP,SLC12A6, SPINK5*
**Stemness**	***CTNNA1,****CDK6,RUNX2, SEMA6D,SMAD3,TP63*
**Cancer metabolism**	***PIK3CA,****CERT1,DGKH, EXOC4,EXT1,GNG12, GNPAT,HKDC1,HMGCL, HPGD,MKLN1,MME,NCOA1, PDE1C,PDGFC,PLA2R1, RAPGEF4,SOS2, SUCLG2,TPK1,VLDLR*

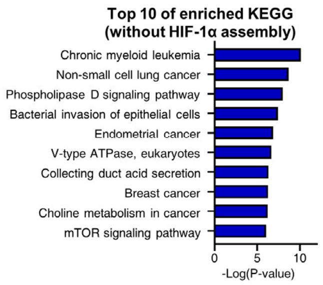

n

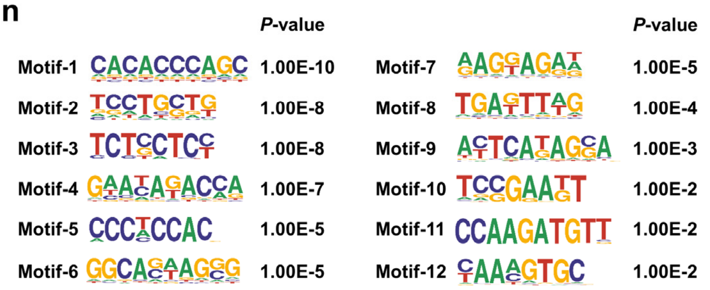

o

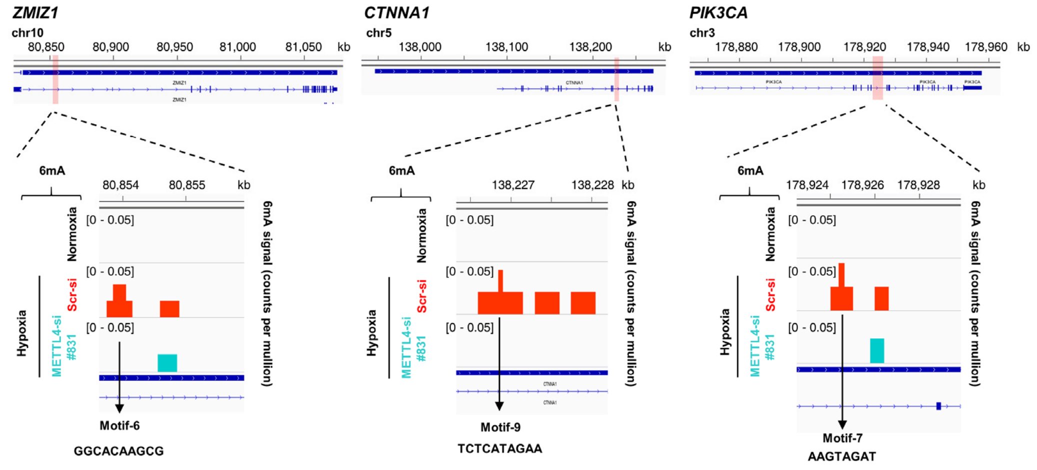

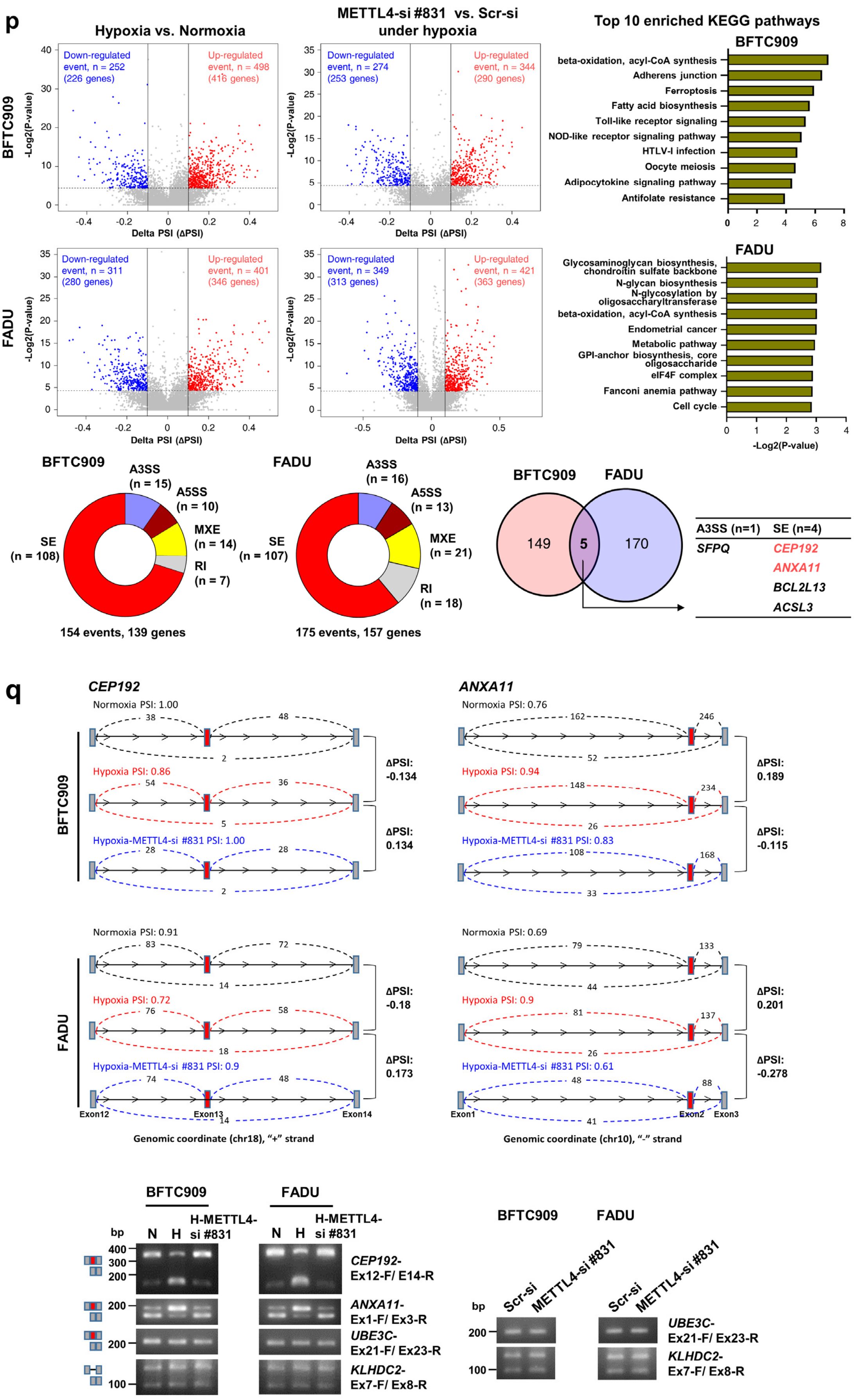
p
Hypoxia vs. Normoxia
METTL4-si #831 vs. Scr-si under hypoxia
Top 10 enriched KEGG pathways
BFTC909
Down-regulated event, n = 252 (226 genes)
Up-regulated event, n = 498 (418 genes)
Down-regulated event, n = 274 (253 genes)
Up-regulated event, n = 344 (290 genes)
-Log2(P-value)
Delta PSI (ΔPSI)
beta-oxidation, acyl-CoA synthesis
Adherens junction
Ferroptosis
Fatty acid biosynthesis
Toll-like receptor signaling
NOD-like receptor signaling pathway
HTLV-I infection
Oocyte meiosis
Adipocytokine signaling pathway
Antifolate resistance
FADU
Down-regulated event, n = 311 (280 genes)
Up-regulated event, n = 401 (346 genes)
Down-regulated event, n = 349 (313 genes)
Up-regulated event, n = 421 (363 genes)
Glycosaminoglycan biosynthesis, chondroitin sulfate backbone
N-glycan biosynthesis
N-glycosylation by oligosaccharyltransferase
beta-oxidation, acyl-CoA synthesis
Endometrial cancer
Metabolic pathway
GPI-anchor biosynthesis, core oligosaccharide
eIF4F complex
Fanconi anemia pathway
Cell cycle
-Log2(P-value)
BFTC909
A3SS (n = 15)
A5SS (n = 10)
MXE (n = 14)
RI (n = 7)
SE (n = 108)
154 events, 139 genes
FADU
A3SS (n = 16)
A5SS (n = 13)
MXE (n = 21)
RI (n = 18)
SE (n = 107)
175 events, 157 genes
BFTC909
FADU
149
5
170
A3SS (n=1)
SFPQ
SE (n=4)
CEP192
ANXA11
BCL2L13
ACSL3
q
CEP192
ANXA11
BFTC909
Normoxia PSI: 1.00
Hypoxia PSI: 0.86
Hypoxia-METTL4-si #831 PSI: 1.00
ΔPSI: -0.134
ΔPSI: 0.134
Normoxia PSI: 0.76
Hypoxia PSI: 0.94
Hypoxia-METTL4-si #831 PSI: 0.83
ΔPSI: 0.189
ΔPSI: -0.115
FADU
Normoxia PSI: 0.91
Hypoxia PSI: 0.72
Hypoxia-METTL4-si #831 PSI: 0.9
ΔPSI: -0.18
ΔPSI: 0.173
Normoxia PSI: 0.69
Hypoxia PSI: 0.9
Hypoxia-METTL4-si #831 PSI: 0.61
ΔPSI: 0.201
ΔPSI: -0.278
Exon12
Exon13
Exon14
Exon1
Exon2
Exon3
Genomic coordinate (chr18), "+" strand
Genomic coordinate (chr10), "-" strand
BFTC909
FADU
bp
N
H
H-METTL4-si #831
CEP192-Ex12-F/ E14-R
ANXA11-Ex1-F/ Ex3-R
UBE3C-Ex21-F/ Ex23-R
KLHDC2-Ex7-F/ Ex8-R
Scr-si
METTL4-si #831
UBE3C-Ex21-F/ Ex23-R
KLHDC2-Ex7-F/ Ex8-R

r

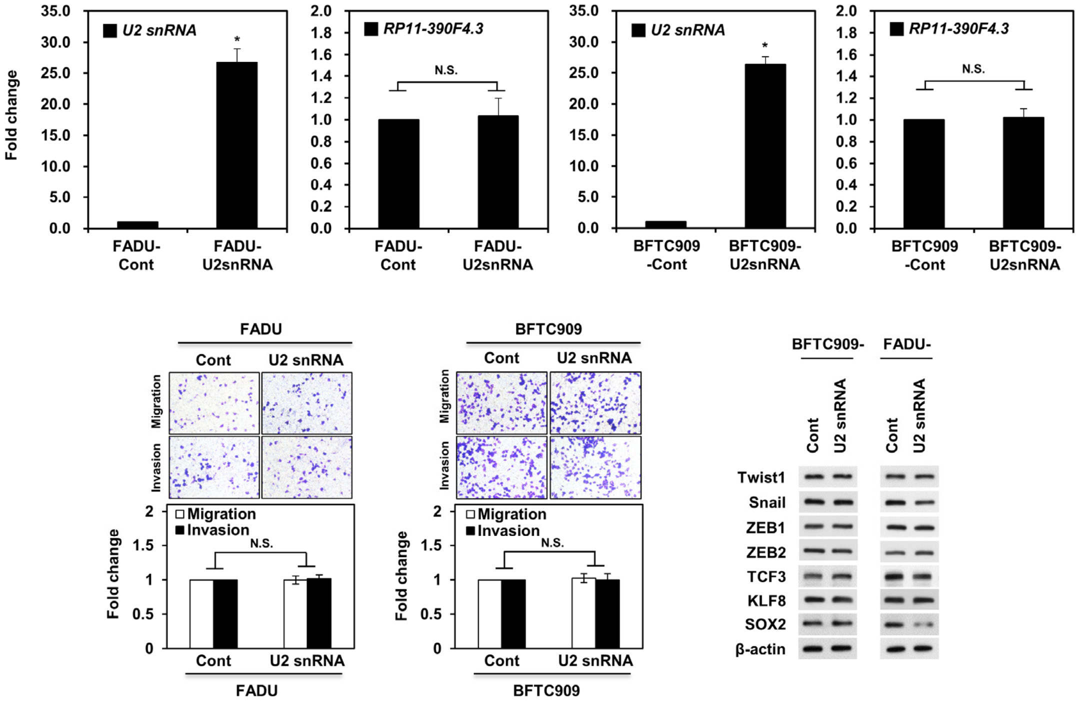
U2 snRNA
RP11-390F4.3
Fold change
N.S.
FADU-Cont
FADU-U2snRNA
BFTC909-Cont
BFTC909-U2snRNA
FADU
BFTC909
Cont
U2 snRNA
Migration
Invasion
BFTC909-
FADU-
Twist1
Snail
ZEB1
ZEB2
TCF3
KLF8
SOX2
β-actin

s

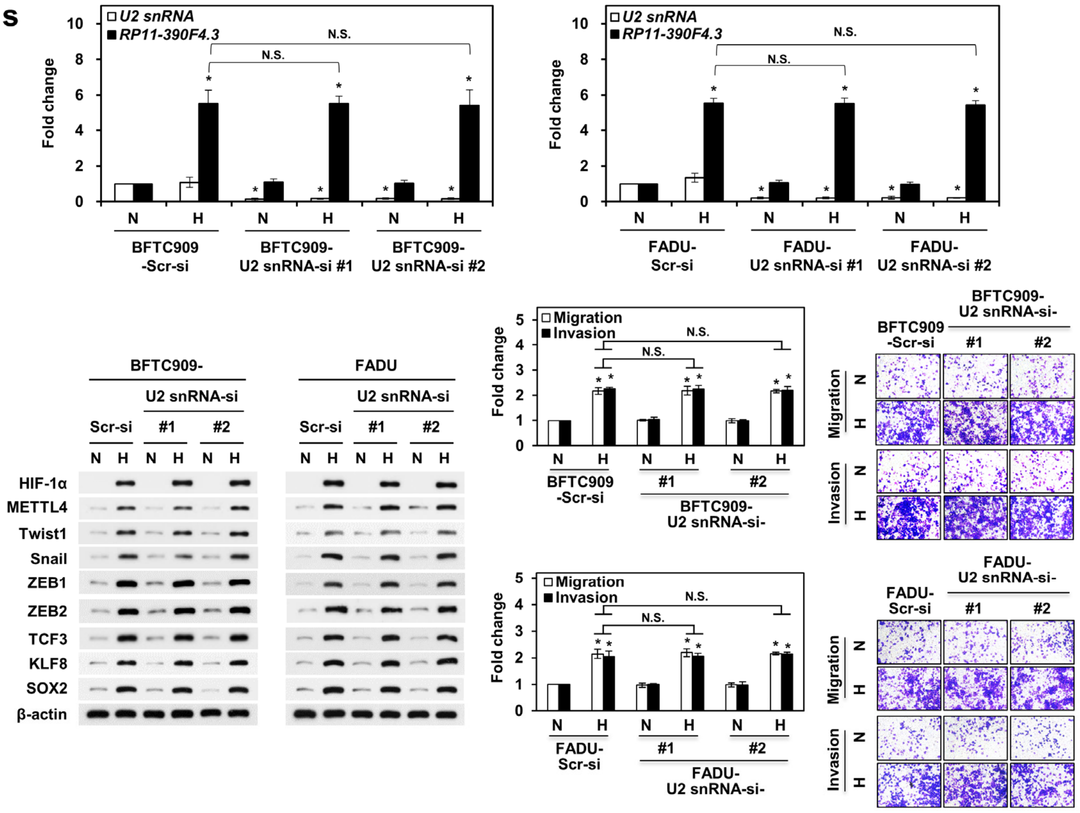
U2 snRNA
RP11-390F4.3
Fold change
N.S.
N
H
BFTC909-Scr-si
BFTC909-U2 snRNA-si #1
BFTC909-U2 snRNA-si #2
FADU-Scr-si
FADU-U2 snRNA-si #1
FADU-U2 snRNA-si #2
BFTC909-
FADU
U2 snRNA-si
Scr-si
#1
#2
HIF-1α
METTL4
Twist1
Snail
ZEB1
ZEB2
TCF3
KLF8
SOX2
β-actin
Migration
Invasion
BFTC909-U2 snRNA-si-
FADU-U2 snRNA-si-

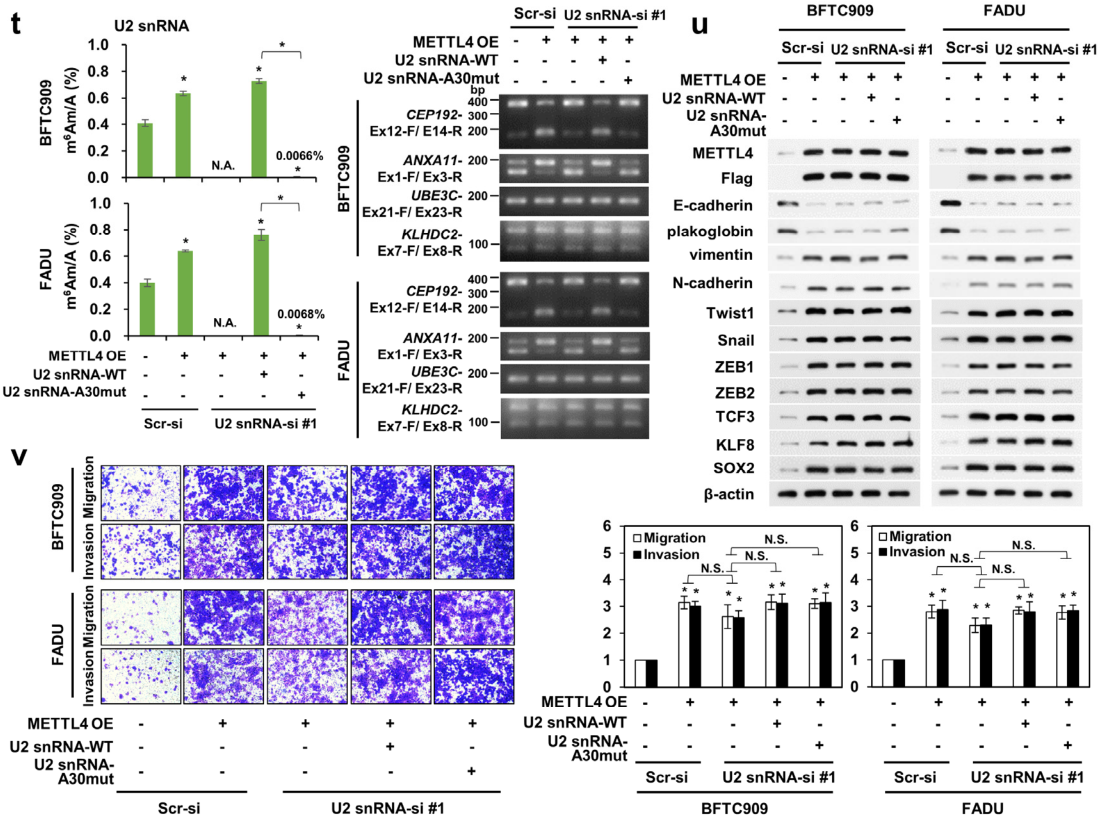
t
U2 snRNA
BFTC909 m6Am/A (%)
FADU m6Am/A (%)
N.A.
0.0066%
0.0068%
METTL4 OE
U2 snRNA-WT
U2 snRNA-A30mut
Scr-si
U2 snRNA-si #1
CEP192-Ex12-F/ E14-R
ANXA11-Ex1-F/ Ex3-R
UBE3C-Ex21-F/ Ex23-R
KLHDC2-Ex7-F/ Ex8-R
bp
u
BFTC909
FADU
METTL4
Flag
E-cadherin
plakoglobin
vimentin
N-cadherin
Twist1
Snail
ZEB1
ZEB2
TCF3
KLF8
SOX2
β-actin
v
Migration
Invasion
N.S.

Figure S4. GSEA of RNA-seq datasets, DE analysis of RNA-seq datasets, Western blot analysis of EMT regulators, signaling connection of METTL4 and lncRNA *RP11-390F4.3,* heatmap analysis of hypoxia-activated lncRNAs, quality control and correlation analysis of 6mA signals between different conditions, 6mA signal analysis and profiling using two different comparisons, consensus sequences of 6mA signal regions, examples of 6mA-deposited genes, profiling of differential RNA splicing events, confirmation of splicing event pattern changes, and knockdown of *U2 snRNA* followed by various assays.

(a-b) Gene Set Enrichment Analysis (GSEA) showed two major gene sets (hypoxia and EMT) could be positively enriched in our RNA-seq results (hypoxia vs normoxia), and negatively enriched with knockdown of *METTL4* under hypoxia.

(c) Differential (DE) gene analysis showed the numbers of significantly regulated genes under hypoxia or *METTL4* knockdown under hypoxia in FADU and BFTC909 with the consideration of 1.3 positive/negative fold change with p-value < 0.05.

(d) Western blot analysis showed the multiple EMT regulators (Snai2, FOXC2, etc) that were not activated by lncRNA *RP11-390F4.3* overexpression in two different cell lines. The cell clone transfected with the control vector was used as a control.

(e) Knockdown of *METTL4* decreased the expression of lncRNA *RP11-390F4.3* under hypoxia in two different cell lines by real-time PCR analysis. Knockdown using the scrambled siRNA was used as a control. The asterisk (*) indicated statistical significance (P<0.05) between experimental and control conditions.

(f) Heatmap analysis showed that most of the reported hypoxia-activated lncRNAs were up-regulated, but only some of them were regulated by METTL4.

(g) Gene ontology of different biological processes were shown for repressed genes co-regulated by hypoxia and METTL4 (left panel). Gene Set Enrichment analysis (GSEA) showed that mitochondrial process, chromatin stability, and tRNA metabolic process were enriched in repressed genes co-regulated by hypoxia and METTL4.

(h) Quality control of 6mA-ChIP-exo-seq results showed the enriched fragment numbers from different sequencing datasets. The bar plot showed that a higher percentage of A could be enriched in 6mA ChIP-exo-seq (including WGA control). Genome-wide 6mA signal profiling across the genes in different conditions including normoxia (black), hypoxia (red) and hypoxic status undergoing *METTL4* knockdown (blue). Right lower panel showed the 6mA signals in hypoxia condition after subtracting WGA background signals.

(i) Correlation of differential 6mA region analysis between hypoxia and *METTL4* knockdown status under hypoxia showed a significant negative correlation in both results of comparison 1 and 2, indicating significant gain-of-6mA regions in red dots (comparison 1, n = 8,265; comparison 2, n = 76,695).

(j) Graph showed the log2 fold change of 6mA signals generated from hypoxia-induced 6mA signal regions (after subtracting 6mA WGA control signals). Venn diagram showed the number of intersected 6mA signal regions between comparison 1, comparison 2, and hypoxia 6mA signal regions (after subtracting 6mA WGA control signals) and 3673 6mA signal regions were identified after the triple dataset overlapping.
(k) Correlation of hypoxia-induced/METTL4 dependent 6mA regulated regions with gene activation or gene repression was comparable. Graph showed the log2 fold change of different RNA-seq comparison results of hypoxia vs. normoxia or hypoxic status undergoing METTL4 knockdown vs. hypoxia after correlating hypoxia-induced/METTL4 dependent 6mA to gene expression. In the result of hypoxia vs. normoxia, 408 increased-expression genes and 256 decreased-expression genes were shown. In the result of hypoxic status undergoing *METTL4* knockdown vs. hypoxia, 342 increased-expression genes and 387 decreased-expression genes were shown.
(l) Venn diagram showed that after overlapping gene expression data with hypoxia-induced/METTL4 dependent gain-of-6mA regions, 263 genes were identified as hypoxia-induced/METTL4 dependent 6mA regulated genes. Analyses of 6mA ChIP-exo-seq and RNA-seq datasets from FADU cells were performed. The top 10 enriched KEGG pathways in hypoxia-induced/METTL4 dependent 6mA regulated genes were shown.
(m) After overlapping hypoxia-induced/METTL4 dependent 6mA regulated genes with HIF-1α ChIP-seq data, 54 HIF-1α assembly-dependent genes and 209 HIF-1α assembly-independent genes were categorized. After functional gene ontology annotation of genes without HIF-1α assembly to their loci, three major cellular functional pathways induced by hypoxia (angiogenesis, stemness, and cancer metabolism) were categorized. The results of top 10 enriched KEGG pathways in 6mA-upregulated genes with or without HIF-1α assembly to their loci were shown.
(n) 6mA motifs were calculated by HOMER from the 6mA signals in hypoxia-induced/METTL4 dependent gain-of-6mA regions from up-regulated genes. *P*-values were shown on the right of each motif.
(o) The examples of hypoxia-induced/METTL4 dependent 6mA up-regulated gene (*ZMIZ1*) and two 6mA-upregulated genes without HIF-1α assembly to their loci (*CTNNA1*-stemness, and *PIK3CA*-cancer metabolism) were shown. All three genes contained 6mA consensus motifs in their introns. Different 6mA motifs calculated by HOMER were identified on the intron 1 of *ZMIZ1* (homology with motif- 6) and different motifs homologous to the different consensus sequences were indicated underneath the intron of *CTNNA1* (motif-9), and *PIK3CA* (motif-7) genes. A magnified window around the designated motif area for each gene was shown. Only motif-6 on the intron 1 of *ZMIZ1*, motif-9 on the intron 10 of *CTNNA1*, and motif-7 on the intron

6 of *PIK3CA* were shown. These motifs were further analyzed on Fig. S6.

(p) Volcano plots showed the genome-wide alternative splicing event analysis through comparison of hypoxia vs. normoxia and METTL4-si vs. scrambled-si under hypoxia in BFTC909 and FADU cells. Up-regulated events were shown in red dots and down-regulated events were shown in blue dots (left upper part). Pie plots showed the number of different differential splicing events regulated by METTL4 under hypoxia in BFTC909 (A3SS, n = 15; A5SS, n = 10; MXE, n = 14; RI, n = 7; SE, n = 108) and FADU (A3SS, n = 16; A5SS, n = 13; MXE, n = 21; RI, n = 18; SE, n = 107) cells (left lower part). Bar graphs showed the top 10 enriched KEGG pathways of differential splicing events in BFTC909 and FADU cells (right upper part). Venn diagram showed 5 events (A3SS, n = 1; SE, n = 4) that were regulated by METTL4 under hypoxia in both BFTC909 and FADU cells (right lower part). A3SS, alternative 3' sites; A5SS, alternative 5' sites; MXE, mutually exclusive exons; RI, retention of introns; SE, skipped exon.

(q) Graphs showed the differential splicing events (exon inclusion or exclusion) of *CEP192* and *ANXA11* in the condition of hypoxia vs. normoxia and METTL4-si vs. scrambled-si under hypoxia in BFTC909 and FADU cells (upper part). Validation of alternative splicing events in the *CEP192*, *ANXA11*, *UBE3C*, and *KLHDC2* genes by RT-PCR with gene-specific primers. The identity of splicing products by exon inclusion or exclusion is schematically shown next to the gels. Differential splicing of *CEP192* and *ANXA11* genes were regulated by hypoxia and returned to the original splicing pattern under METTL4 knockdown (left lower part). The *UBE3C* and *KLHDC2* genes were not regulated by hypoxia or METTL4 (right lower part).

(r) Overexpression of *U2 snRNA* did not increase the mRNA levels of *RP11-390F4.3*, the *in vitro* migration/invasion activity, or the protein levels of various EMT regulators in two cell lines. Representative photos of the *in vitro* migration and invasion activity were shown in this panel. The cell clone transfected with the control vector was used as a control. The asterisk (*) indicated statistical significance ($P<0.05$) between experimental and control groups. N.S., not statistically significant.

(s) Knockdown of *U2 snRNA* did not change the RNA levels of *RP11-390F4.3*, the protein levels of different EMT regulators induced by hypoxia, or the *in vitro* migration/invasion activity induced by hypoxia. Representative photos of the *in vitro* migration and invasion activity were shown. Two different siRNAs were used to knockdown *U2 snRNA*. N, normoxia; H, hypoxia. Knockdown using the scrambled siRNA under normoxia condition was used as a control. The asterisk (*) indicated statistical significance ($P<0.05$) between experimental and control conditions. N.S., not statistically significant.

(t) The m6Am methylation levels of these reconstituted *U2 snRNAs* (wild type vs. A30

point mutant) were measured by UPLC-ESI-MS/MS (left part). Knockdown of *U2 snRNA* under METTL4 overexpression status followed by reconstitution with either wild type *U2 snRNA* or point mutant *U2 snRNA* experiments were performed. Assays of differential splicing showed that wild type *U2 snRNA* was able to rescue the differential splicing events of *CEP192* and *ANXA11* induced by METTL4 overexpression, but not the A30-mutated *U2 snRNA* (right part).

(u) Knockdown of *U2 snRNA* under METTL4 overexpression status followed by reconstitution with either wild type *U2 snRNA* or point mutant *U2 snRNA* did not change the protein levels of various EMT regulators or EMT markers by Western blot analysis.

(v) Knockdown of *U2 snRNA* under METTL4 overexpression status followed by reconstitution with either wild type *U2 snRNA* or point mutant *U2 snRNA* did not change the *in vitro* migration and invasion activity of two different cell lines. Representative photos of the *in vitro* migration and invasion activity were shown. Knockdown using the scrambled siRNA was used as a control. The asterisk (*) indicated statistical significance (P<0.05) between experimental and control groups. N.S., not statistically significant.

Figure S5

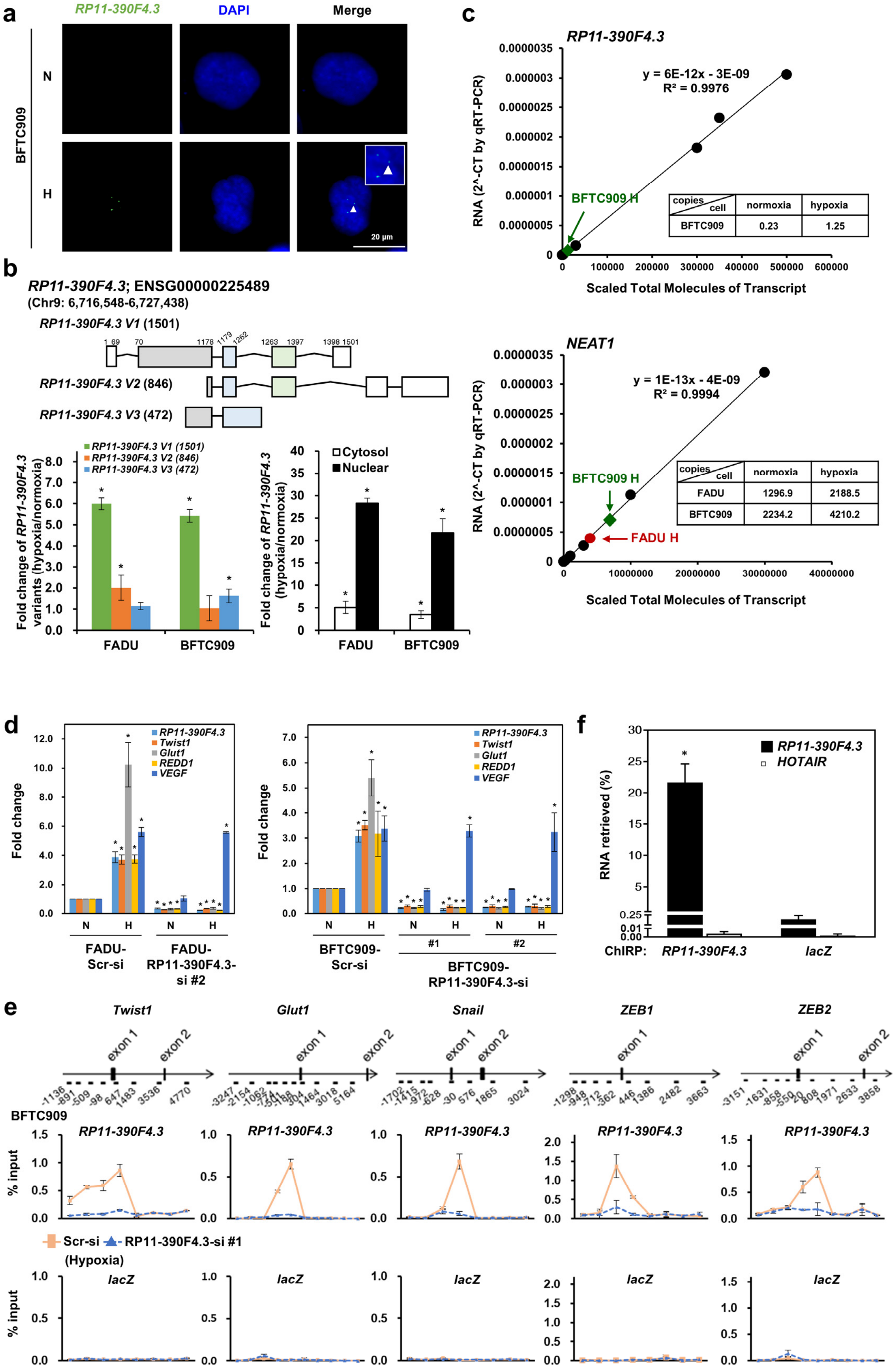

copies / cell	normoxia	hypoxia
BFTC909	0.23	1.25

copies / cell	normoxia	hypoxia
FADU	1296.9	2188.5
BFTC909	2234.2	4210.2

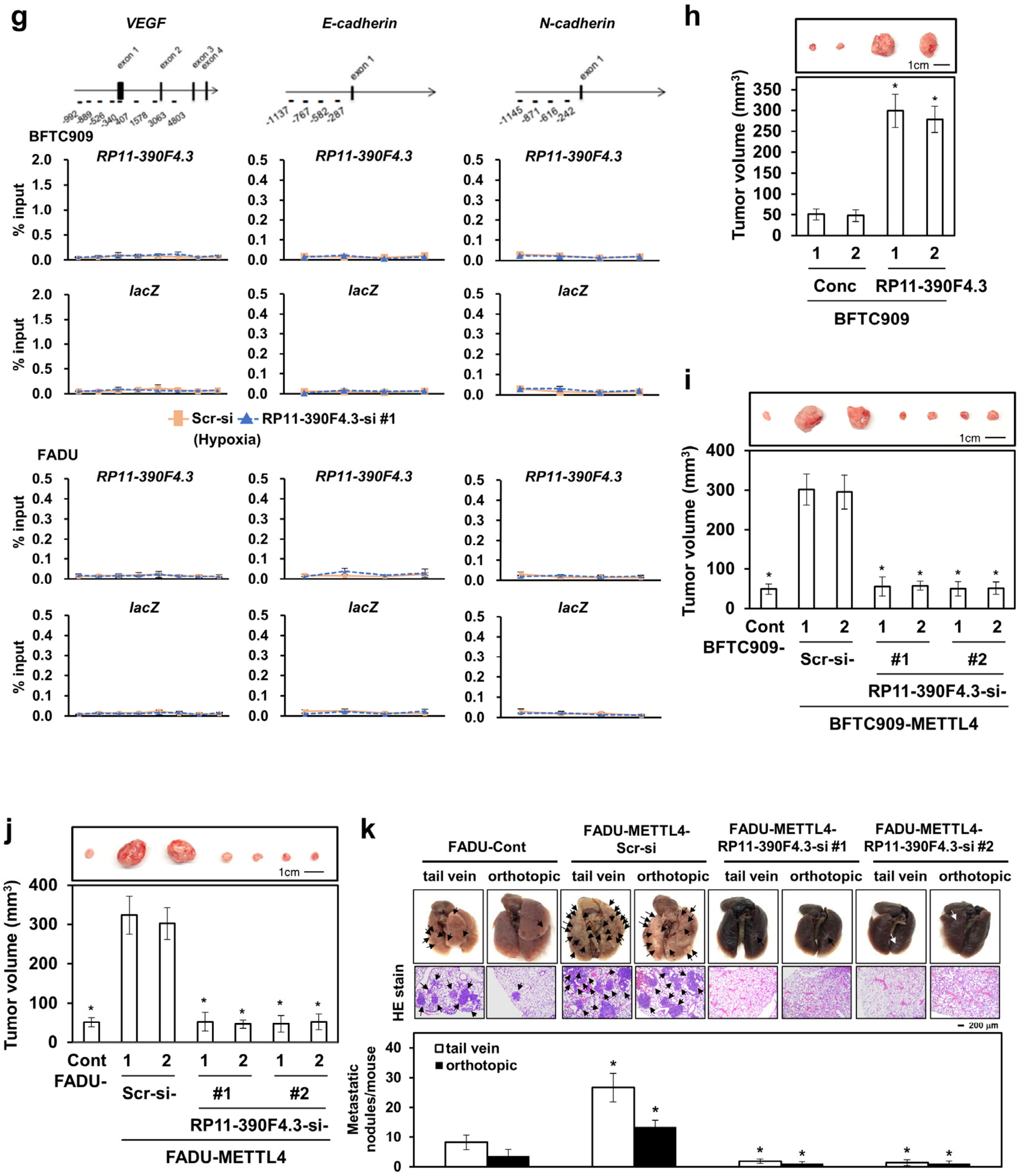

l

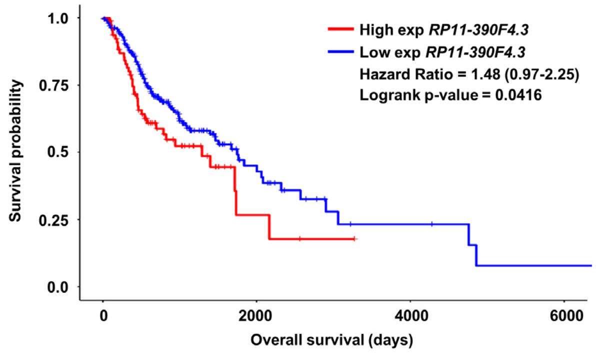

RP11-390F4.3 expression level	number of patients
High express (Top 25% of log2CPM > -1.5)	79
low express (Bottom 75% of log2CPM > -1.5)	236
No express (Log2CPM < -1.5)	185

Figure S5. RNA FISH assay, analysis of different versions of lncRNA *RP11-390F4.3*, copy number of lncRNA *RP11-390F4.3*, ChIRP peaks of three different EMT regulator genes, *in vivo* tumorigenicity assay of lncRNA *RP11-390F4.3*, and the TCGA survival analysis of head and neck cancer patient groups with high lncRNA *RP11-390F4.3* levels.

(a) RNA FISH staining showed the increased nuclear staining of lncRNA *RP11-390F4.3* in BFTC909 cells under hypoxia. Cell nuclei were stained by DAPI. The white frame line represents the result of the magnification of the cell. N, normoxia; H, hypoxia. Normoxic condition was used as a control.

(b) LncRNA *RP11-390F4.3* V1 had the highest fold of activation (~4 to 6 fold) inside nucleus in FADU and BFTC909 cells using real-time PCR analysis. The diagram shows the genotypes of different variants of lncRNA *RP11-390F4.3* V1 to V3 (upper panel). Normoxic condition was used as a control. The asterisk (*) indicated statistical significance (P<0.05) between experimental and control groups.

(c) Measurement of the copy number of lncRNA *RP11-390F4.3* or *NEAT1* in BFTC909 and FADU cells (normoxia vs. hypoxia). Titration standard curve was used for measurement of the copy number of lncRNA *RP11-390F4.3* or *NEAT1* per 500,000 cells. The green and red points represent the real-time PCR value from a standard sample of 500,000 BFTC909 and FADU cells under hypoxia.

(d) Knockdown of lncRNA *RP11-390F4.3* significantly decreased the induction of various HIF-1α target genes using real-time PCR analysis in BFTC909 and FADU cells (normoxia vs. hypoxia). Knockdown using the scrambled siRNA under normoxia condition was used as a control. Two different siRNAs were used to knockdown *RP11-390F4.3* in BFTC909 cells N, normoxia; H, hypoxia. The asterisk (*) indicated statistical significance (P<0.05) between experimental and control clones.

(e-g) qChIRP assays showed the significantly decreased lncRNA *RP11-390F4.3* binding to the promoter regions of EMT regulator and *Glut1* genes in hypoxic BFTC909 cells undergoing lncRNA *RP11-390F4.3* knockdown compared to the control knockdown hypoxic cells. The genomic regions of *Twist1*, *Glut1*, *Snail*, *ZEB1*, and *ZEB2* genes and the regions that were checked by qChIRP assays were shown on the top. Different regions located in the promoters and inside the gene body of these genes were shown underneath the plotting of genes. The different numbers indicated the starting nucleotide positions in the regions (labeled by brackets) before and after the initiation site. Retrieval of lncRNA *RP11-390F4.3,* but not *HOTAIR*, by tiling probes was shown **(f).** The promoters of *VEGF, E-cadherin*, and *N-cadherin* were used as a negative control **(g)**.

(h) Overexpression of lncRNA *RP11-390F4.3* increased the tumor volume of BFTC909 cells using xenograft implantation assays. The cell clone transfected with the

control vector was used as a control. The asterisk (*) indicated statistical significance (P<0.05) between experimental and control groups.
(i-j) Knockdown of lncRNA *RP11-390F4.3* in METTL4-overexpressing BFTC909 **(i)** and FADU **(j)** cells significantly decreased the tumor volume induced by METTL4. The cell clone transfected with the control vector was used as a control. The asterisk (*) indicated statistical significance (P<0.05) between experimental and control groups.
(k) Knockdown of lncRNA *RP11-390F4.3* in FADU cells overexpressing METTL4 significantly decreased the metastatic lung nodules in mice. Representative gross anatomy and histology were shown on the upper panel and measurement of metastatic lung nodules was shown on the lower panel. The cell clone transfected with the control vector was used as a control. The asterisk (*) indicated statistical significance (P<0.05) between experimental and control groups.
(l) Increased expression of lncRNA *RP11-390F4.3* indicated a poor prognosis in head and neck cancer patients from the TCGA dataset analysis.
The error bars represented the standard deviation (SD). Student's *t*-test was used to compare two groups of independent samples. For details, see method section.

Figure S6

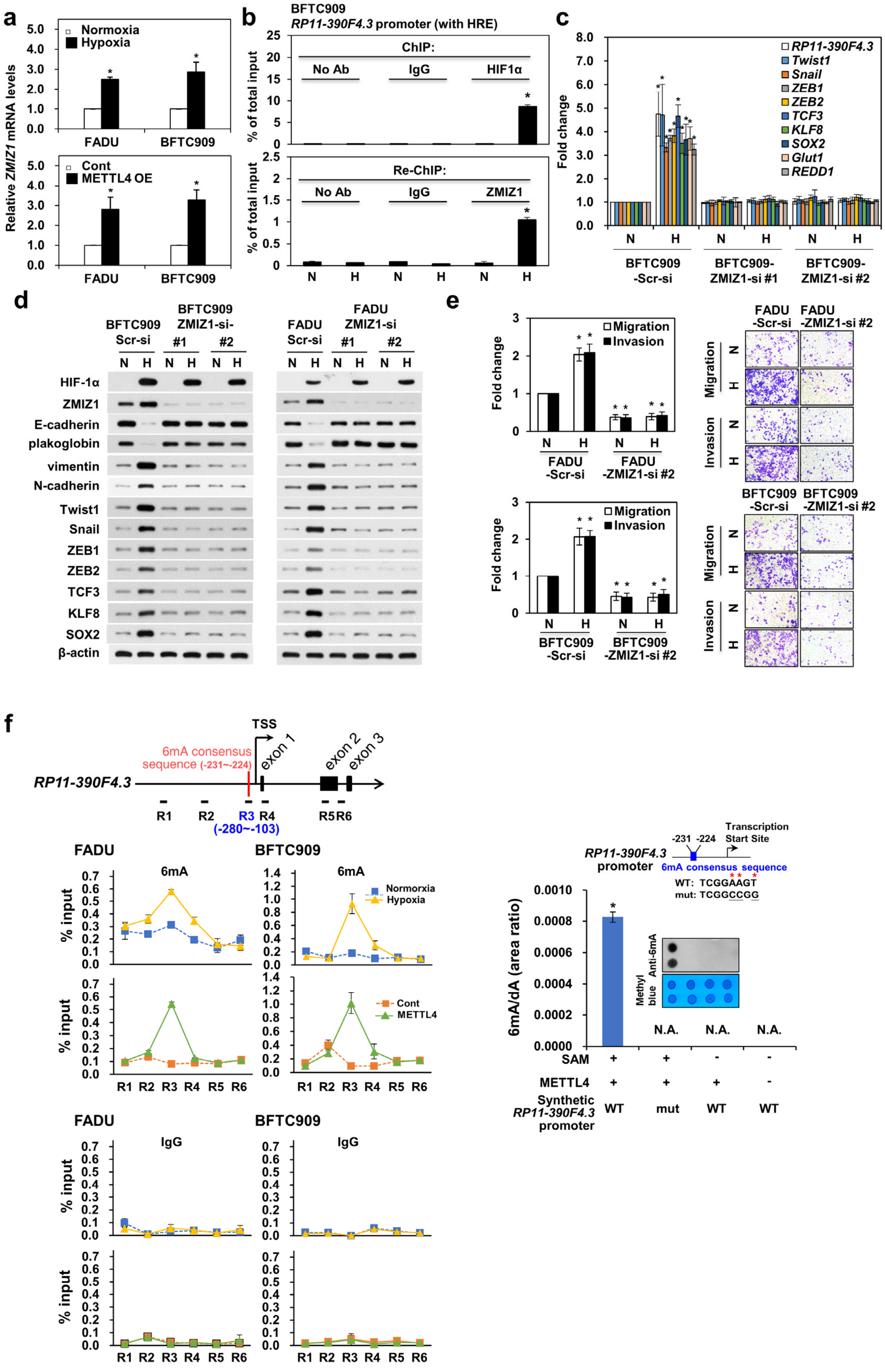

a
Normoxia
Hypoxia
Cont
METTL4 OE
Relative ZMIZ1 mRNA levels
FADU
BFTC909
b
BFTC909
RP11-390F4.3 promoter (with HRE)
ChIP:
Re-ChIP:
No Ab
IgG
HIF1α
ZMIZ1
% of total input
c
RP11-390F4.3
Twist1
Snail
ZEB1
ZEB2
TCF3
KLF8
SOX2
Glut1
REDD1
Fold change
BFTC909 -Scr-si
BFTC909- ZMIZ1-si #1
BFTC909- ZMIZ1-si #2
d
BFTC909 Scr-si
BFTC909 ZMIZ1-si- #1 #2
FADU Scr-si
FADU ZMIZ1-si #1 #2
HIF-1α
ZMIZ1
E-cadherin
plakoglobin
vimentin
N-cadherin
Twist1
Snail
ZEB1
ZEB2
TCF3
KLF8
SOX2
β-actin
e
Migration
Invasion
Fold change
FADU -Scr-si
FADU -ZMIZ1-si #2
BFTC909 -Scr-si
BFTC909 -ZMIZ1-si #2
f
TSS
6mA consensus sequence (-231~-224)
exon 1
exon 2
exon 3
RP11-390F4.3
R1 R2 R3 R4 R5 R6
(-280~-103)
FADU
BFTC909
6mA
IgG
Normorxia
Hypoxia
Cont
METTL4
% input
RP11-390F4.3 promoter
-231 -224
Transcription Start Site
6mA consensus sequence
WT: TCGGAAGT
mut: TCGGCCGG
6mA/dA (area ratio)
Anti-6mA
Methyl blue
N.A.
SAM
METTL4
Synthetic RP11-390F4.3 promoter
WT
mut

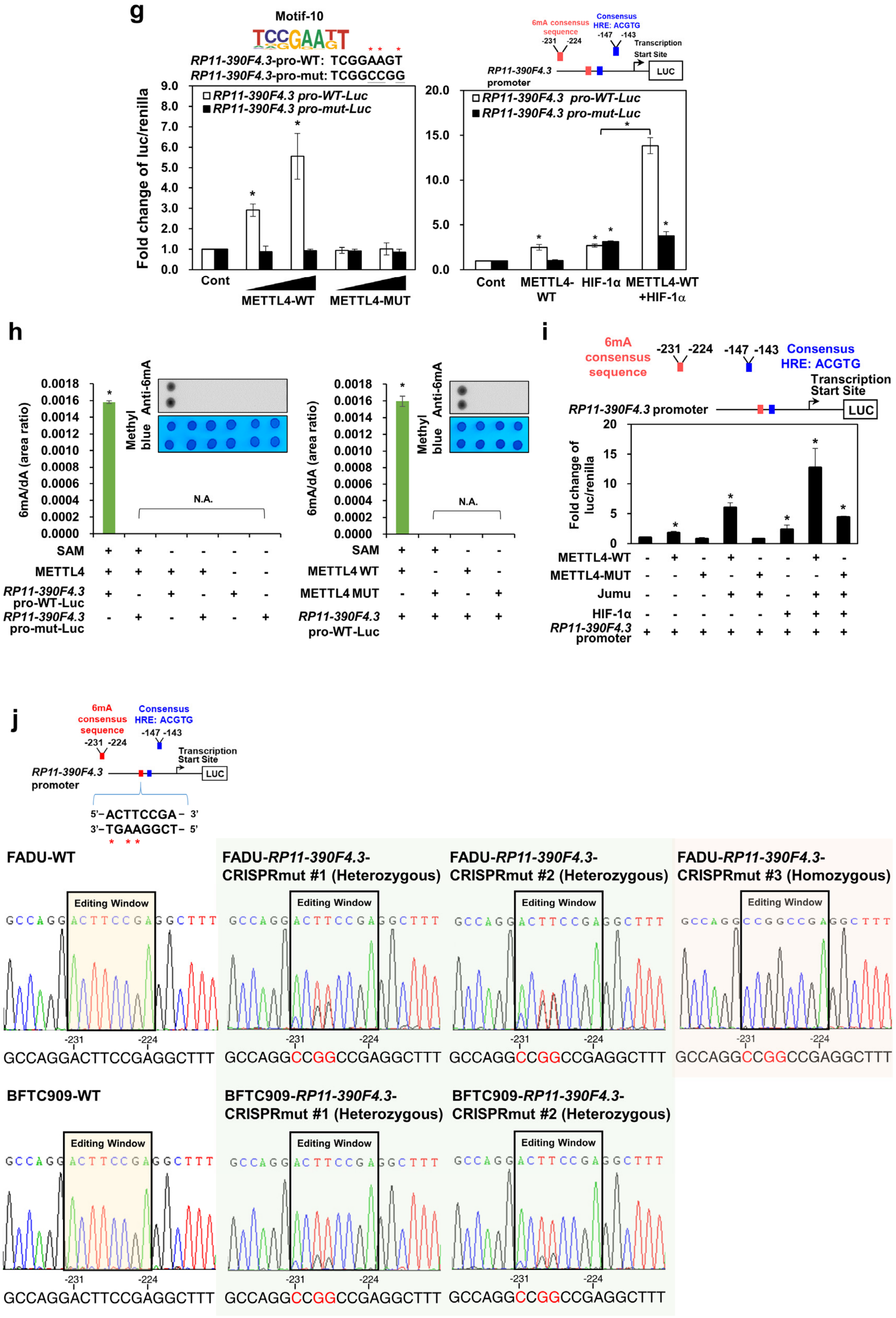
g
Motif-10
RP11-390F4.3-pro-WT: TCGGAAGT
RP11-390F4.3-pro-mut: TCGGCCGG
Fold change of luc/renilla
RP11-390F4.3 pro-WT-Luc
RP11-390F4.3 pro-mut-Luc
Cont
METTL4-WT
METTL4-MUT
6mA consensus sequence
Consensus HRE: ACGTG
Transcription Start Site
RP11-390F4.3 promoter
LUC
METTL4-WT
HIF-1α
METTL4-WT +HIF-1α
h
6mA/dA (area ratio)
Anti-6mA
Methyl blue
N.A.
SAM
METTL4
RP11-390F4.3 pro-WT-Luc
RP11-390F4.3 pro-mut-Luc
METTL4 WT
METTL4 MUT
i
Jumu
METTL4-MUT
RP11-390F4.3 promoter
j
5'-ACTTCCGA- 3'
3'-TGAAGGCT- 5'
FADU-WT
FADU-RP11-390F4.3-CRISPRmut #1 (Heterozygous)
FADU-RP11-390F4.3-CRISPRmut #2 (Heterozygous)
FADU-RP11-390F4.3-CRISPRmut #3 (Homozygous)
Editing Window
GCCAGGACTTCCGAGGCTTT
GCCAGGCCGGCCGAGGCTTT
BFTC909-WT
BFTC909-RP11-390F4.3-CRISPRmut #1 (Heterozygous)
BFTC909-RP11-390F4.3-CRISPRmut #2 (Heterozygous)

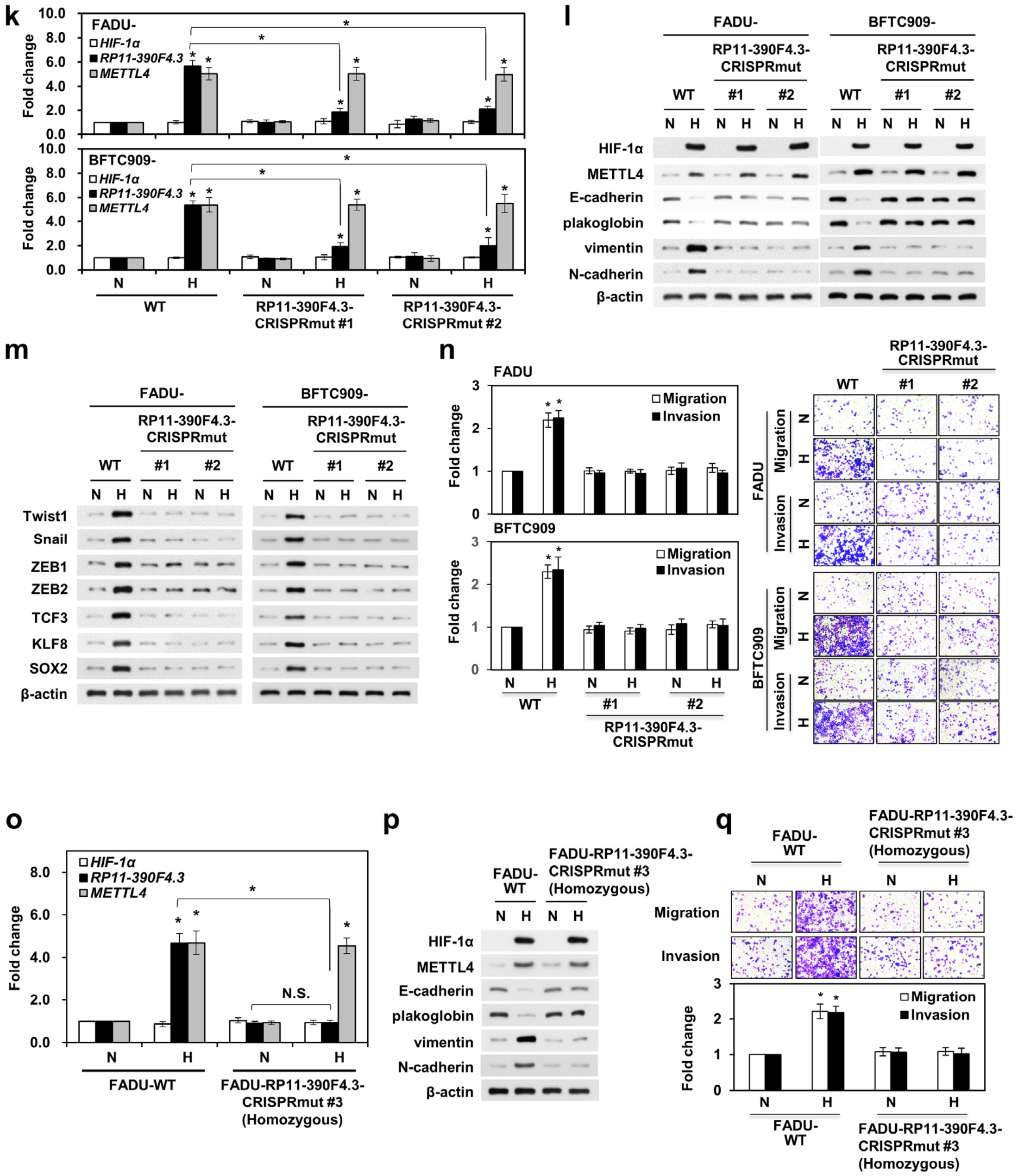
k
FADU-
HIF-1α
RP11-390F4.3
METTL4
BFTC909-
Fold change
10.0
8.0
6.0
4.0
2.0
0.0
N
H
WT
RP11-390F4.3-CRISPRmut #1
RP11-390F4.3-CRISPRmut #2
l
FADU-
BFTC909-
RP11-390F4.3-CRISPRmut
WT
#1
#2
HIF-1α
METTL4
E-cadherin
plakoglobin
vimentin
N-cadherin
β-actin
m
Twist1
Snail
ZEB1
ZEB2
TCF3
KLF8
SOX2
β-actin
n
FADU
BFTC909
Migration
Invasion
RP11-390F4.3-CRISPRmut
o
FADU-WT
FADU-RP11-390F4.3-CRISPRmut #3 (Homozygous)
N.S.
p
FADU-WT
FADU-RP11-390F4.3-CRISPRmut #3 (Homozygous)
q
FADU-WT
FADU-RP11-390F4.3-CRISPRmut #3 (Homozygous)
Migration
Invasion

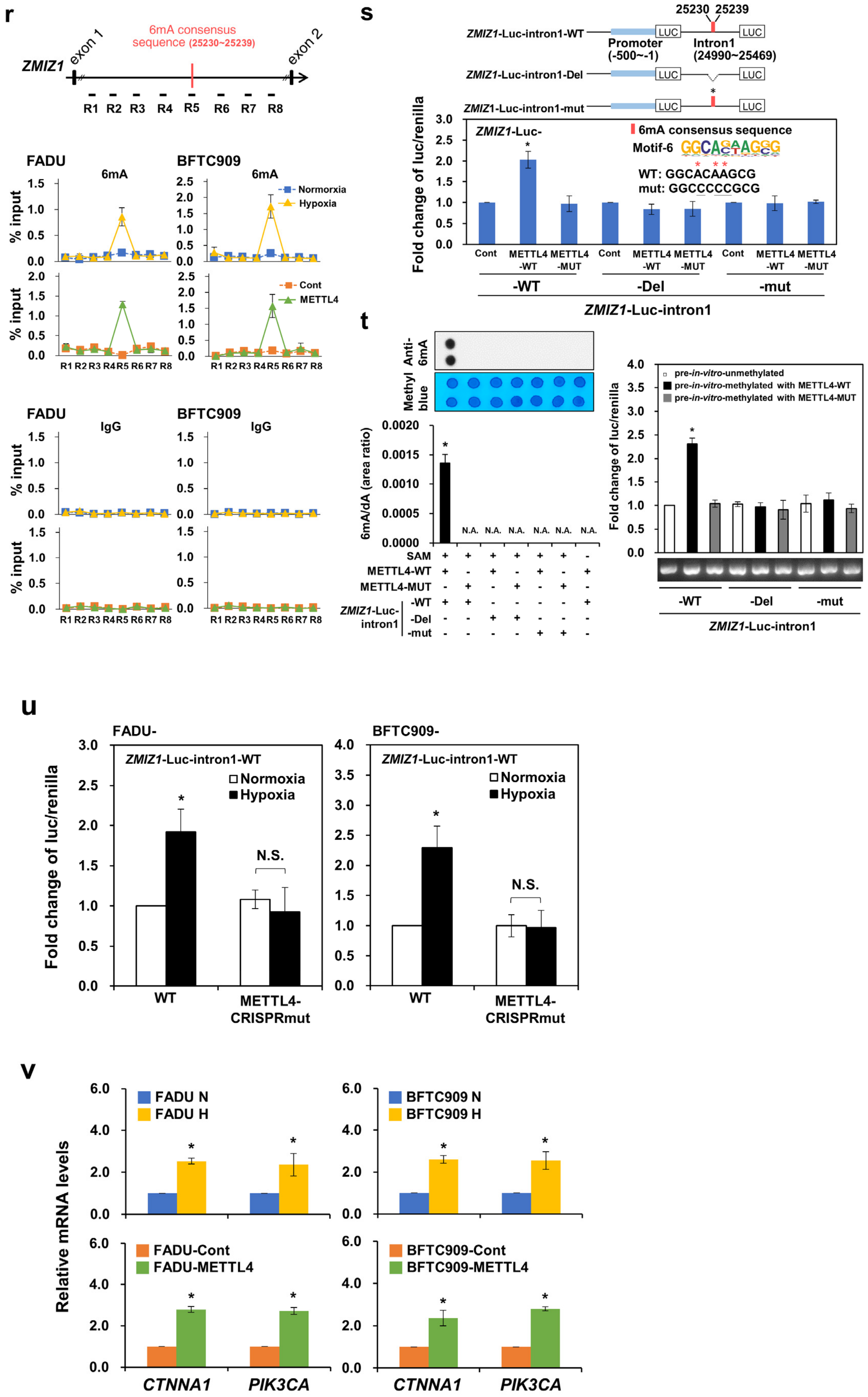
r
ZMIZ1
exon 1
exon 2
6mA consensus sequence (25230~25239)
R1 R2 R3 R4 R5 R6 R7 R8
FADU
BFTC909
6mA
IgG
% input
Normorxia
Hypoxia
Cont
METTL4
s
ZMIZ1-Luc-intron1-WT
ZMIZ1-Luc-intron1-Del
ZMIZ1-Luc-intron1-mut
Promoter (-500~-1)
Intron1 (24990~25469)
25230 25239
LUC
ZMIZ1-Luc-
6mA consensus sequence
Motif-6
WT: GGCACAAGCG
mut: GGCCCCCGCG
Fold change of luc/renilla
Cont
METTL4 -WT
METTL4 -MUT
-WT
-Del
-mut
ZMIZ1-Luc-intron1
t
Anti-6mA
Methyl blue
6mA/dA (area ratio)
N.A.
SAM
METTL4-WT
METTL4-MUT
ZMIZ1-Luc-intron1
pre-in-vitro-unmethylated
pre-in-vitro-methylated with METTL4-WT
pre-in-vitro-methylated with METTL4-MUT
u
FADU-
BFTC909-
ZMIZ1-Luc-intron1-WT
Normoxia
Hypoxia
N.S.
WT
METTL4-CRISPRmut
v
Relative mRNA levels
FADU N
FADU H
BFTC909 N
BFTC909 H
FADU-Cont
FADU-METTL4
BFTC909-Cont
BFTC909-METTL4
CTNNA1
PIK3CA

w

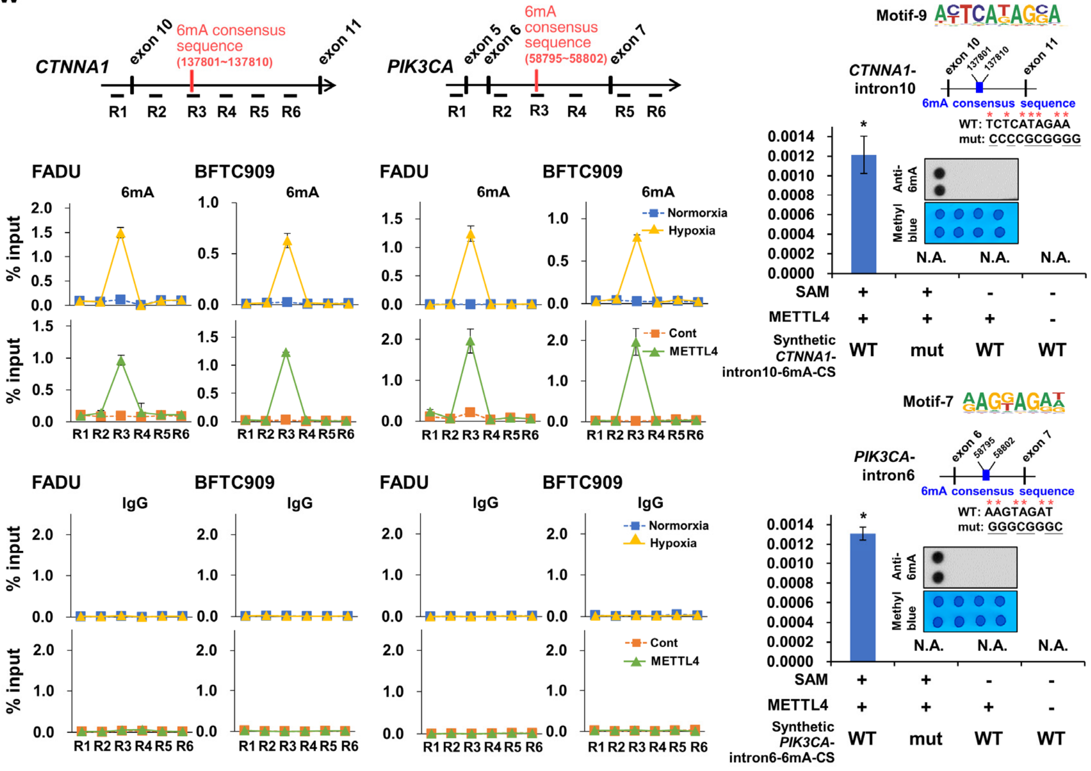

CTNNA1
exon 10
6mA consensus sequence (137801~137810)
exon 11
R1 R2 R3 R4 R5 R6
PIK3CA
exon 5
exon 6
6mA consensus sequence (58795~58802)
exon 7
FADU
BFTC909
6mA
IgG
% input
Normorxia
Hypoxia
Cont
METTL4
Motif-9
CTNNA1-intron10
6mA consensus sequence
WT: TCTCATAGAA
mut: CCCCGCGGGG
Anti-6mA
Methyl blue
N.A.
SAM
METTL4
Synthetic CTNNA1-intron10-6mA-CS
WT
mut
Motif-7
PIK3CA-intron6
WT: AAGTAGAT
mut: GGGCGGGC
Synthetic PIK3CA-intron6-6mA-CS

x

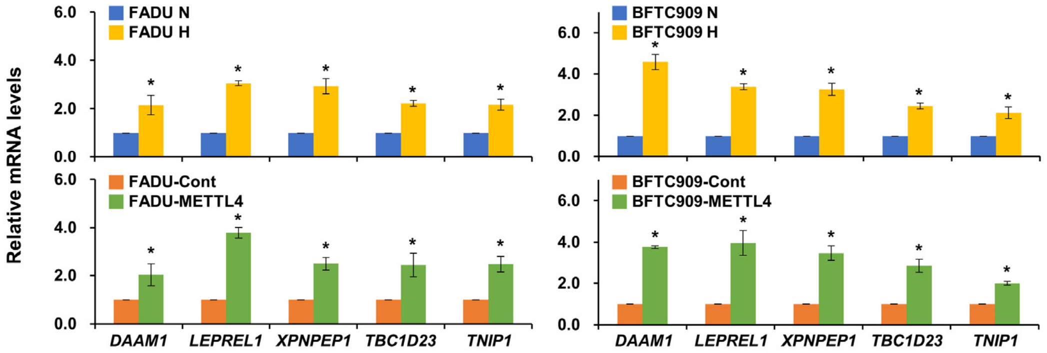

Relative mRNA levels
FADU N
FADU H
BFTC909 N
BFTC909 H
FADU-Cont
FADU-METTL4
BFTC909-Cont
BFTC909-METTL4
DAAM1
LEPREL1
XPNPEP1
TBC1D23
TNIP1

y

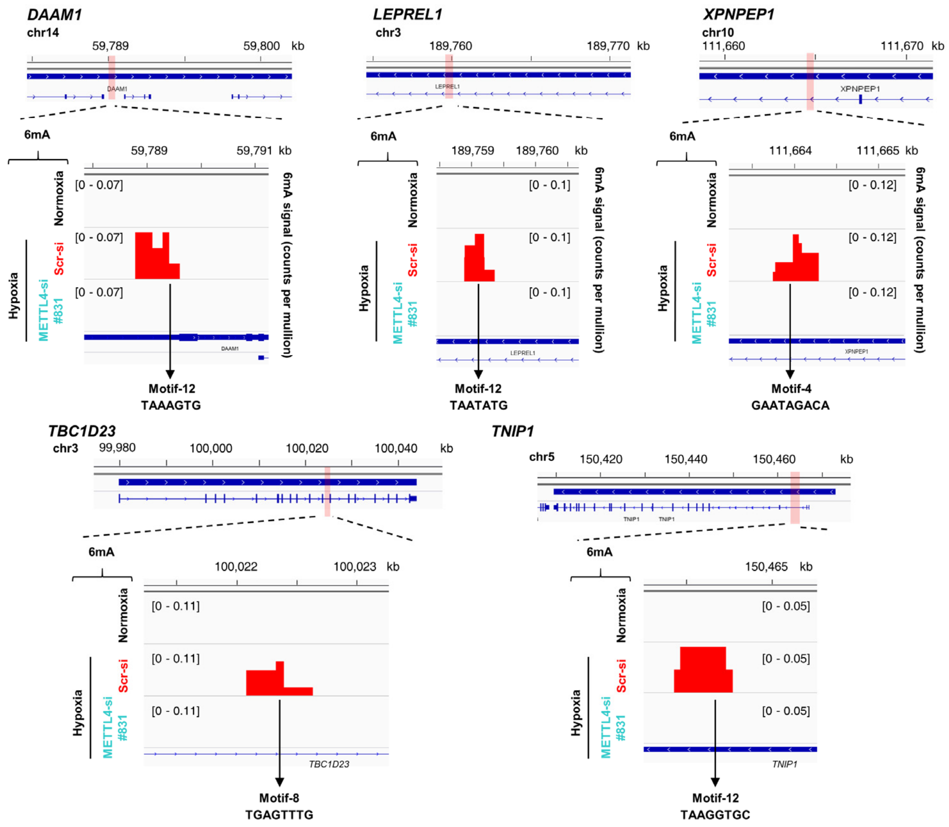
DAAM1
chr14
LEPREL1
chr3
XPNPEP1
chr10
6mA
Normoxia
Hypoxia
Scr-si
METTL4-si #831
6mA signal (counts per million)
Motif-12
TAAAGTG
Motif-12
TAATATG
Motif-4
GAATAGACA
TBC1D23
chr3
TNIP1
chr5
Motif-8
TGAGTTTG
Motif-12
TAAGGTGC

z

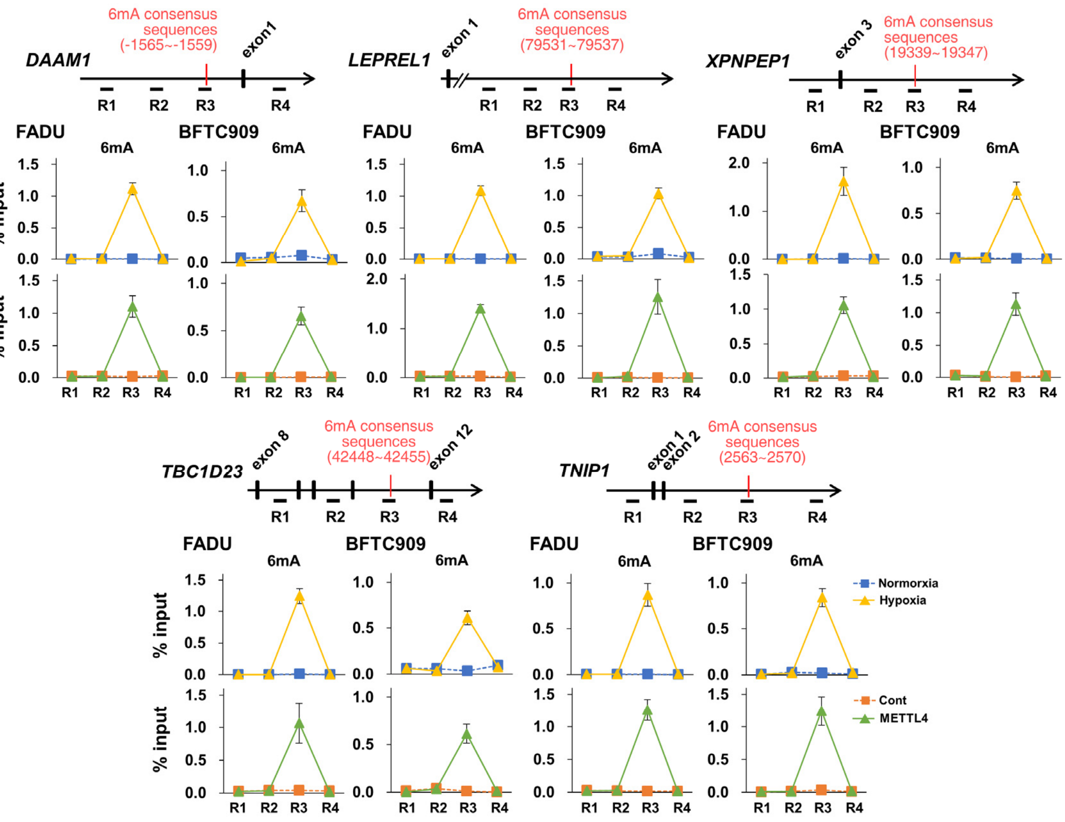
DAAM1
6mA consensus sequences (-1565~-1559)
exon1
LEPREL1
exon 1
6mA consensus sequences (79531~79537)
XPNPEP1
exon 3
6mA consensus sequences (19339~19347)
R1
R2
R3
R4
FADU
BFTC909
6mA
% input
TBC1D23
exon 8
6mA consensus sequences (42448~42455)
exon 12
TNIP1
exon 1
exon 2
6mA consensus sequences (2563~2570)
Normorxia
Hypoxia
Cont
METTL4

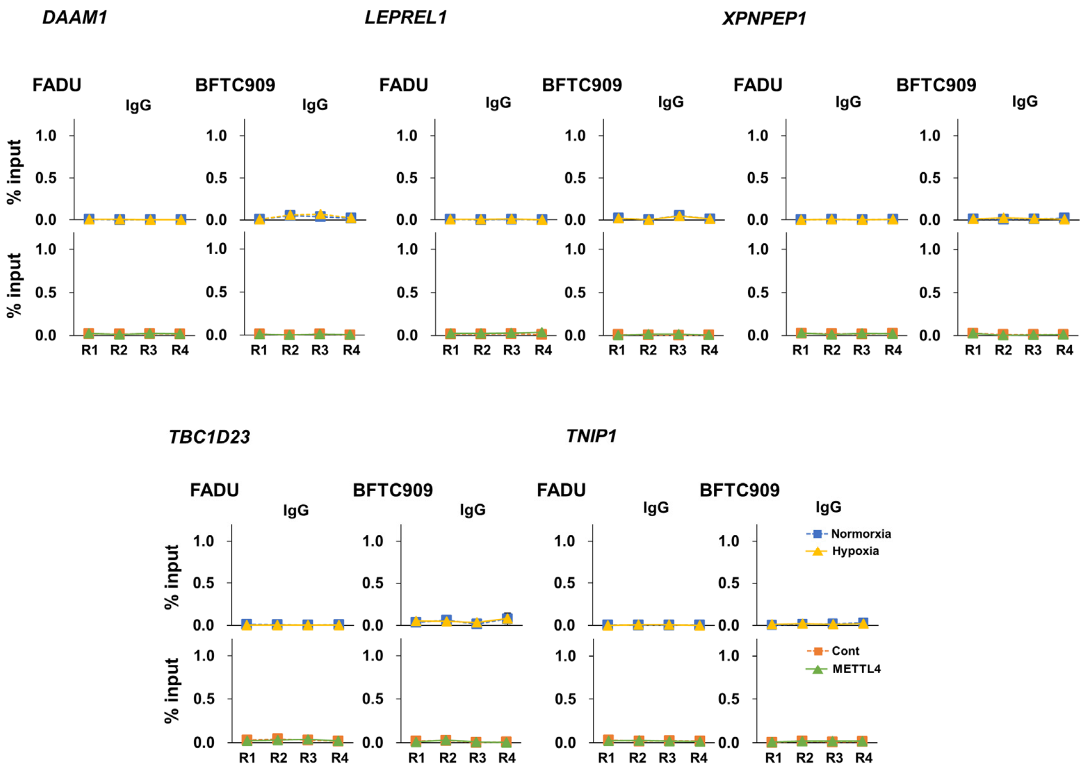
DAAM1
LEPREL1
XPNPEP1
TBC1D23
TNIP1
FADU
BFTC909
IgG
% input
1.0
0.5
0.0
R1
R2
R3
R4
Normorxia
Hypoxia
Cont
METTL4

Figure S6. Real-time PCR analysis of gene expression, ChIP-re-ChIP assays, Western blot analysis and in vitro migration/invasion activity of two cell lines with *ZMIZ1* knockdown under hypoxia, *in vitro* DNA methylation assays to confirm the 6mA sites methylated by METTL4, the activation of the 6mA site-containing *ZMIZ1* reporter gene construct by METTL4, DNA sequencing analysis of 6mA site mutation on the lncRNA *RP11-390F4.3* promoter, real-time PCR, Western blot, and *in vitro* migration/invasion activity assays of cells with heterozygous or homozygous mutation of the 6mA site on the lncRNA *RP11-390F4.3* promoter, reporter gene assays of the pre-methylated *ZMIZ1* reporter construct, and *in vitro* methylation assays to the 6mA site on the introns of *CTNNA1* or *PIK3CA* gene.

(a) Hypoxia or METTL4 overexpression increased the *ZMIZ1* mRNA levels in FADU and BFTC909 cell lines. The asterisk (*) indicated statistical significance ($P<0.05$) between experimental and control groups. Normoxic condition and the cell clone transfected with the control vector were used as a control.

(b) ChIP-re-ChIP assays in BFTC909 cell showed that ZMIZ1 could be pulled down after chromatin immunoprecipitation to pull down HIF-1α, indicating the co-existence of HIF-1α and ZMIZ1 in the transcription complex on the lncRNA *RP11-390F4.3* promoter (with hypoxia response element, HRE). N, normoxia; H, hypoxia. No antibody/normoxia condition was used as a control. The asterisk (*) indicated statistical significance ($P<0.05$) between experimental and control groups.

(c) Knockdown of *ZMIZ1* significantly decreased the induction of various HIF-1α target genes in BFTC909 cells by real-time PCR analyses. Two different siRNAs were used to knockdown *ZMIZ1*. Knockdown using the scrambled siRNA under normoxia condition was used as a control. N, normoxia; H, hypoxia. The asterisk (*) indicated statistical significance ($P<0.05$) between experimental and control condition.

(d) Knockdown of *ZMIZ1* abolished hypoxia-induced EMT in BFTC909 and FADU cell lines by Western blot analysis. Knockdown using the scrambled siRNA under normoxia condition was used as a control. N, normoxia; H, hypoxia. The asterisk (*) indicated statistical significance ($P<0.05$) between experimental and control condition.

(e) Knockdown of *ZMIZ1* decreased the *in vitro* migration and invasion activity induced by hypoxia in FADU or BFTC909 cell lines. A different siRNA (compared to the siRNA used in Fig. 6d) was used to knockdown *ZMIZ1* gene. This experiment was performed at a different time (compared to Fig. 6d). Representative photos of the *in vitro* migration and invasion activity were shown in this panel. N, normoxia; H, hypoxia. Knockdown using the scrambled siRNA under normoxia condition was used as a control. The asterisk (*) indicated statistical significance ($P<0.05$) between experimental and control groups.

(f) MeDIP assays showed the presence of 6mA site on the promoter of lncRNA *RP11-*

390F4.3. IgG was used as a control. The figure above shows the 6mA consensus sequence (–231 to –224 upstream of TSS of the lncRNA *RP11-390F4.3* gene) in the promoter R3 region (–280 to –103 upstream of TSS) of the lncRNA *RP11-390F4.3* gene. METTL4 increased the 6mA levels on the oligonucleotides containing the motif-10 of the lncRNA *RP11-390F4.3* promoter using *in vitro* methylation assays, whereas no increased 6mA levels could be shown on the oligonucleotides containing mutated 6mA sequences. The oligonucleotide only was used as a control. The asterisk (*) indicated statistical significance (P<0.05) between experimental and control groups. Corresponding 6mA dot blots with methyl blue loading controls were shown together with bar graphs.

(g) Reporter gene assays showed that METTL4, but not the METTL4 mutant, activated the lncRNA *RP11-390F4.3* gene promoter driven reporter construct. No activation of the lncRNA *RP11-390F4.3* gene promoter driven reporter constructs when the 6mA consensus sequence was mutated. The 6mA consensus sequence of wild type and mutant on the lncRNA *RP11-390F4.3* gene promoter are shown in the top (left panel). METTL4 and HIF-1α synergistically activated the *RP11-390F4.3* promoter-driven reporter construct that contained a 6mA consensus sequence (-231 to -224 upstream of TSS of the *RP11-390F4.3* gene) and a HIF-1α response element (HRE, -147 to -143 upstream of TSS of the *RP11-390F4.3* gene) (right panel). No synergistic activation of the *RP11-390F4.3* promoter-driven reporter constructs when the 6mA consensus sequence was mutated. The position of the 6mA consensus sequence on the *RP11-390F4.3* promoter and the 6mA consensus sequence of wild and mutation are shown in the figure above (right panel). The luciferase/renilla activities of FADU cells co-transfected with reporter construct and pcDNA3 control vector under normoxia were used as the baseline control. The asterisk (*) indicated statistical significance (P<0.05) between experimental and control groups.

(h) The 6mA levels of the *RP11-390F4.3*-driven wild type reporter construct that underwent *in vitro* methylation (vs. a 6mA-site mutated reporter construct) was measured via UPLC-ESI-MS/MS assays (left panel). 6mA methylation was mediated by wild type METTL4, not by the mutated METTL4 (right panel). The reporter construct without incubating with wild type METTL4 and SAM was used as a control. The asterisk (*) indicated statistical significance (P<0.05) between experimental and control groups. Corresponding 6mA dot blots with methyl blue loading controls were shown together with bar graphs.

(i) Reporter gene assays showed that METTL4, HIF-1α, and Jumu (a Drosophila 6mA-binding protein) synergistically activated the *RP11-390F4.3* promoter-driven reporter construct that contained a 6mA consensus sequence. The figure above shows the positions of the 6mA consensus sequence and the consensus HRE on the *RP11-390F4.3*

promoter. The luciferase/renilla activities of FADU cells co-transfected with reporter construct and pcDNA3 control vector under normoxia were used as the baseline control. The asterisk (*) indicated statistical significance ($P<0.05$) between experimental and control group.

(j) Representative sequencing results of the *in vivo* mutation of the 6mA site on the *RP11-390F4.3* promoter in FADU and BFTC909 cells using a prime-cutting CRISPR-Cas9 approach were shown. Mut #3 in FADU cells represented the homozygous mutations of the 6mA site. The wild-type (WT) cells was used as a control.

(k) Mutation of one copy of the 6mA site on the promoter of *RP11-390F4.3* gene already reduced the activation of *RP11-390F4.3* down to ~30% of the wild type *RP11-390F4.3* expression induced by hypoxia in FADU and BFTC909 cells. N, normoxia; H, hypoxia. Normoxic condition of the WT cell line was used as a control. The asterisk (*) indicated statistical significance ($P<0.05$) between experimental and control conditions.

(l-m) The regulation of EMT markers and regulators mediated by hypoxia was abolished in the *RP11-390F4.3* 6mA site heterozygous mutated clones in FADU and BFTC909 cells by Western blot analysis. N, normoxia; H, hypoxia.

(n) The activation of *in vitro* migration and invasion activity induced by hypoxia was abolished in the *RP11-390F4.3* 6mA site heterozygous mutated clones in FADU and BFTC909 cells. Representative photos of the *in vitro* migration and invasion activity were shown on the right. N, normoxia; H, hypoxia. Normoxic condition of the WT cell line was used as a control. The asterisk (*) indicated statistical significance ($P<0.05$) between experimental and control conditions.

(o) The activation of *RP11-390F4.3* by hypoxia was totally abolished in the *RP11-390F4.3* 6mA site homozygous mutated clone in FADU cells. N, normoxia; H, hypoxia. Normoxic condition of the WT cell line was used as a control. The asterisk (*) indicated statistical significance ($P<0.05$) between experimental and control conditions. N.S., not statistically significant.

(p) The regulation of EMT markers mediated by hypoxia was abolished in the *RP11-390F4.3* 6mA site homozygous mutated clone in FADU cells. N, normoxia; H, hypoxia.

(q) The activation of *in vitro* migration and invasion activity induced by hypoxia was abolished in the *RP11-390F4.3* 6mA site homozygous mutated clone in FADU cells. Representative photos of the *in vitro* migration and invasion activity were shown. N, normoxia; H, hypoxia. Normoxic condition of the WT cell line was used as a control. The asterisk (*) indicated statistical significance ($P<0.05$) between experimental and control conditions.

(r) MeDIP assays showed the presence of 6mA site on the intron 1 of the *ZMIZ1* gene. IgG was used as a control. The figure above showed the 6mA consensus sequence (25230 to 25239, from the TSS of *ZMIZ1* gene) in the intron 1 R5 region of *ZMIZ1*

gene.

(s) The reporter construct containing the 6mA consensus sequence (25230 to 25239, start from the *ZMIZ1* gene) in the intron 1 region (24990 to 25469, start from the *ZMIZ1* gene) of *ZMIZ1* was activated by co-expressing METTL4, but not the reporter construct containing the deleted or mutated 6mA sites. The 6mA consensus sequence of wild-type and mutation on the intron 1 of *ZMIZ1* are shown in the top. The luciferase/renilla activities of FADU cells co-transfected with reporter construct and pcDNA3 control vector under normoxia were used as the baseline control. The asterisk (*) indicated statistical significance (P<0.05) between experimental and control groups.

(t) The 6mA levels of the *ZMIZ1* intron 1-driven wild type reporter construct that underwent *in vitro* methylation (vs. a 6mA-site deleted or mutated reporter construct) was measured via UPLC-ESI-MS/MS assays. 6mA methylation was mediated by wild type METTL4, not by the mutated METTL4. The reporter construct without incubating with wild type METTL4 was used as a control. The asterisk (*) indicated statistical significance (P<0.05) between experimental and control groups. Corresponding 6mA dot blots with methyl blue loading controls were shown together with bar graphs. Reporter gene assays showed that the 6mA site pre-methylated *ZMIZ1* intron 1-driven reporter construct had higher luciferase activities compared to the unmethylated construct after transfection. The 6mA site deleted or mutated reporter construct also had only baseline luciferase activities. *In vitro* methylation experiments were performed to generate the 6mA site pre-methylated reporter construct. The luciferase/renilla activities of FADU cells co-transfected with reporter construct and pcDNA3 control vector under normoxia were used as the baseline control. The asterisk (*) indicated statistical significance (P<0.05) between experimental and control groups.

(u) No activation of the reporter gene construct by hypoxia was shown when the reporter construct was transfected into the enzymatically inactive METTL4 mutant FADU and BFTC909 cells. The reporter construct contained the 6mA consensus sequence (25230 to 25239, start from the *ZMIZ1* gene) in the intron 1 region (24990 to 25469, start from the *ZMIZ1* gene) of *ZMIZ1* gene. N, normoxia; H, hypoxia. The luciferase/renilla activities of the wild type cells transfected with reporter construct under normoxia were used as the baseline control. The asterisk (*) indicated statistical significance (P<0.05) between experimental and control conditions. N.S.: not statistically significant.

(v) Real-time PCR assays confirmed the activation of two hypoxia-regulated target genes *(CTNNA1, PIK3CA)* induced by hypoxia or METTL4 overexpression in FADU or BFTC909 cells. The asterisk (*) indicated statistical significance (P<0.05) between experimental and control groups. N, normoxia; H, hypoxia. Normoxic condition and the cell clone transfected with the control vector were used as a control.

(w) MeDIP assays showed the presence of 6mA site on the intron 10 of *CTNNA1* gene and the intron 6 of the *PIK3CA* gene. The red-inked labeling indicated the 6mA consensus sequence in the intronic regions of the HIF-1α indirect target genes. METTL4 increased the 6mA levels on the oligonucleotides containing the 6mA consensus motifs of the intron 10 region of *CTNNA1*, and intron 6 region of *PIK3CA* using *in vitro* DNA methylation assays, whereas no increased 6mA levels could be shown on the oligonucleotides containing mutated 6mA sequences. The oligonucleotide only was used as a control. The asterisk (*) indicated statistical significance (P<0.05) between experimental and control groups. Corresponding 6mA dot blots with methyl blue loading controls were shown together with bar graphs.
(x) Real-time PCR assays confirmed the activation of five hypoxia-regulated target genes *(DAAM1, LEPREL1, XPNPEP1, TBC1D23, and TNIP1)* induced by hypoxia or METTL4 overexpression in FADU or BFTC909 cells. The asterisk (*) indicated statistical significance (P<0.05) between experimental and control groups. N, normoxia; H, hypoxia. Normoxic condition and the cell clone transfected with the control vector were used as a control.
(y) The gene tracks of 5 hypoxia/METTL4 co-regulated genes with their sites of 6mA deposition (*DAAM1, LEPREL1, XPNPEP1, TBC1D23, and TNIP1*) were shown. All five genes contained 6mA consensus motifs in their promoter or introns.
(z) MeDIP assays confirmed the presence of 6mA sites on the genomic locations of *DAAM1, LEPREL1, XPNPEP1, TBC1D23,* and *TNIP1* genes in FADU and BFTC909 cells. The red-inked labeling indicated the 6mA consensus sequence in the promoter or intronic regions of these genes.
The error bars represented the standard deviation (SD). Student's t-test was used to compare two groups of independent samples. For details, see method section.
