# Supplementary material for: METTL4-mediated nuclear N6-deoxyadenosine methylation promotes metastasis through activating multiple metastasis-inducing targets
Source: Genome Biol. 2022 Dec 2;23:249. doi: 10.1186/s13059-022-02819-3 (PMC9716733; doi:10.1186/s13059-022-02819-3)

Source Data 1

Fig. 1a

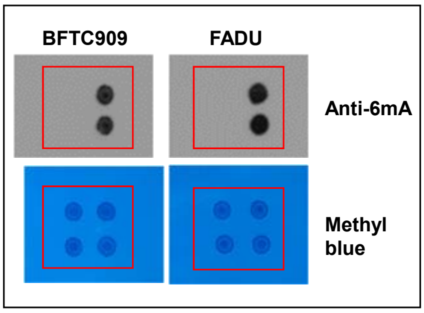

Fig. 1f

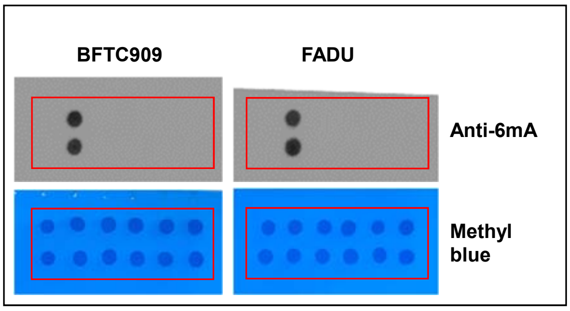

Fig. 2a

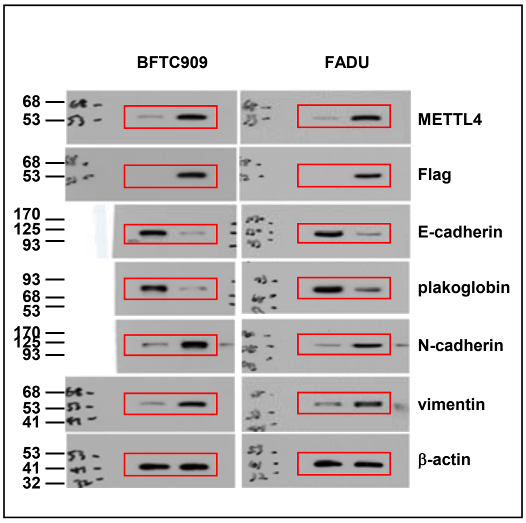

Fig. 1d

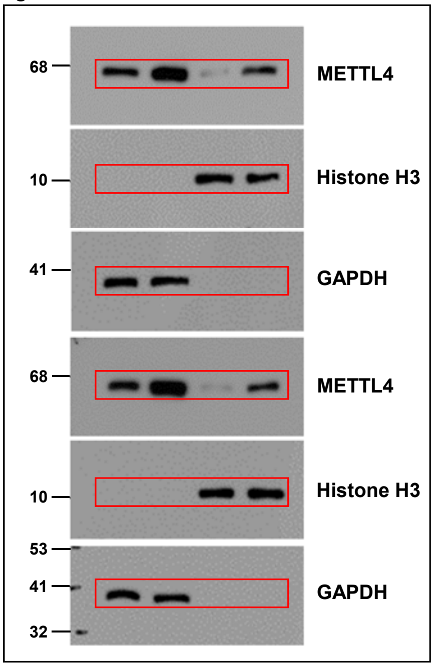

Fig. 1g

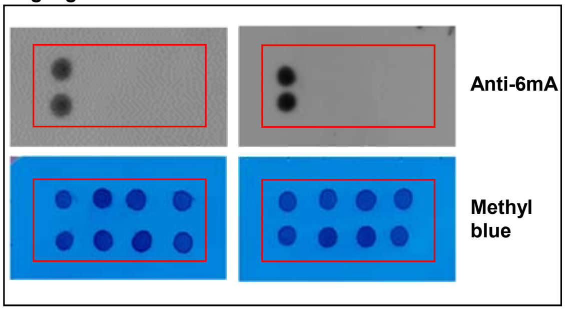

Fig. 2b

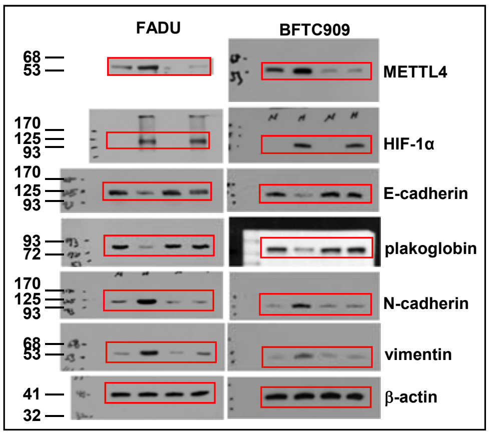

Fig. 2g

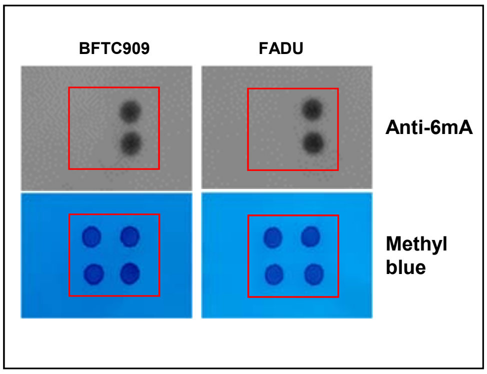

Fig. 2c

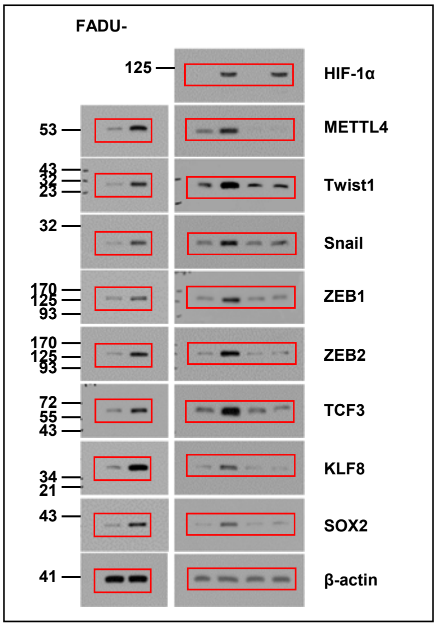

Fig. 3a

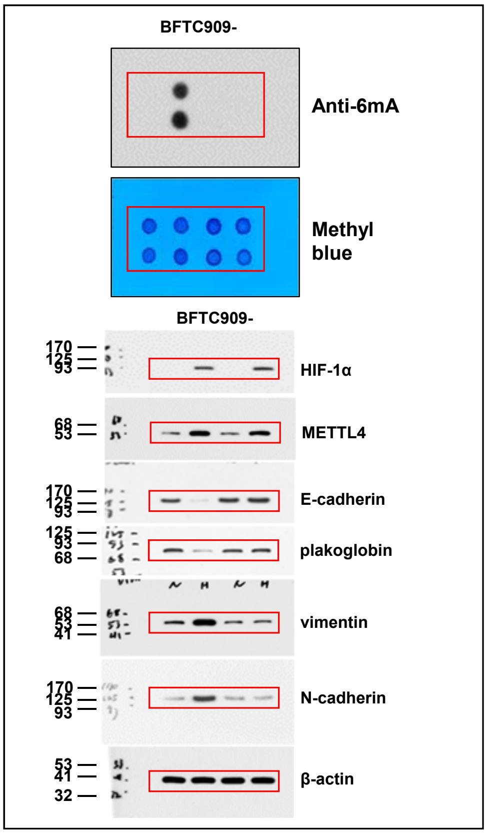

Fig. 4f

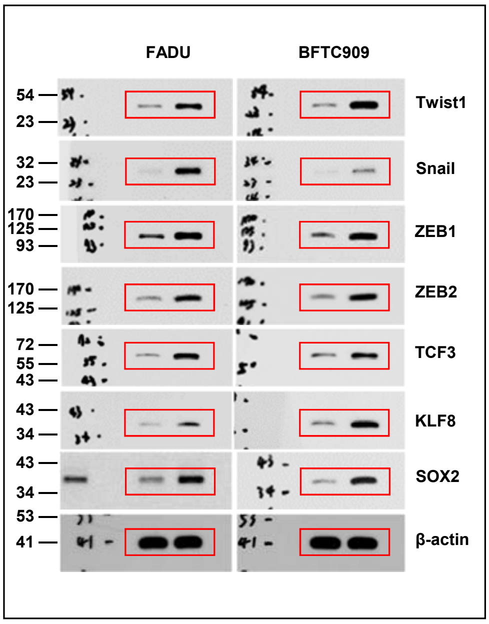

Fig. 2f

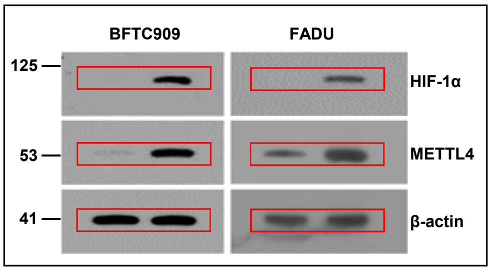

Fig. 5f

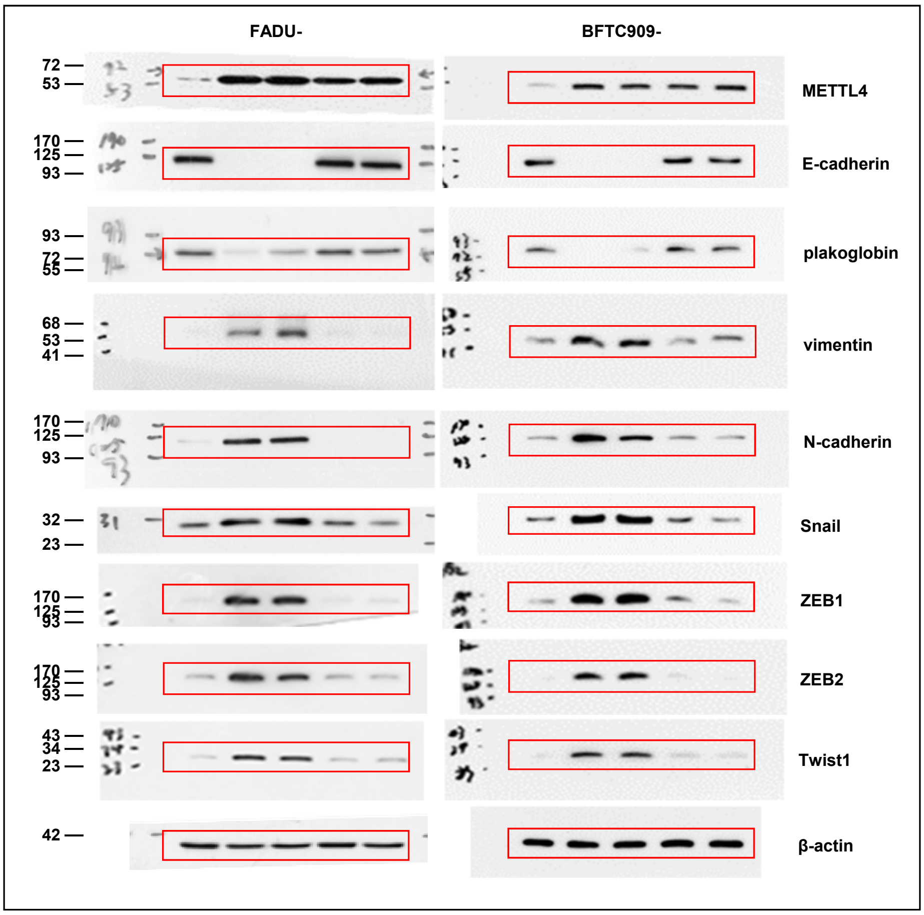

Fig. 6a

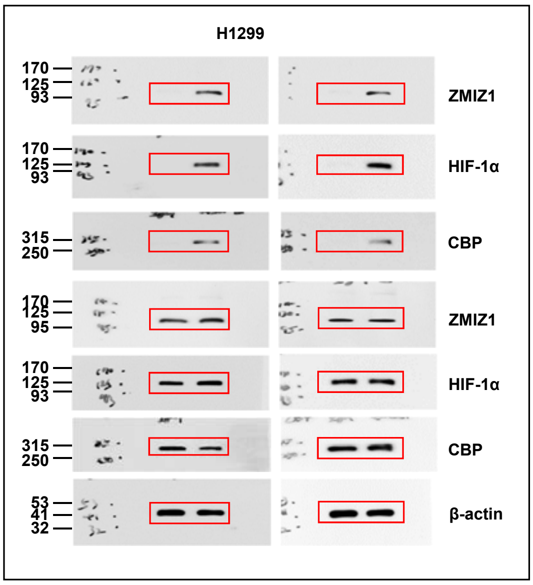

Fig. 6e

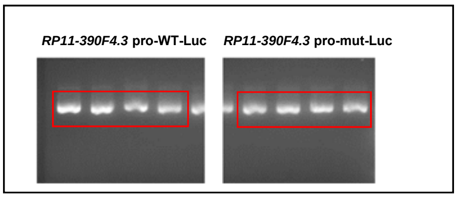

Fig. 6g

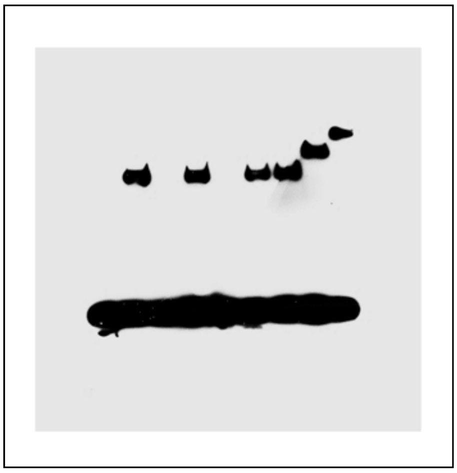

Fig. 6f

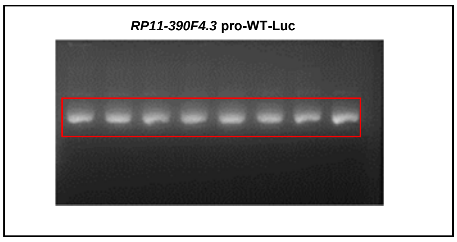

Fig. S1c

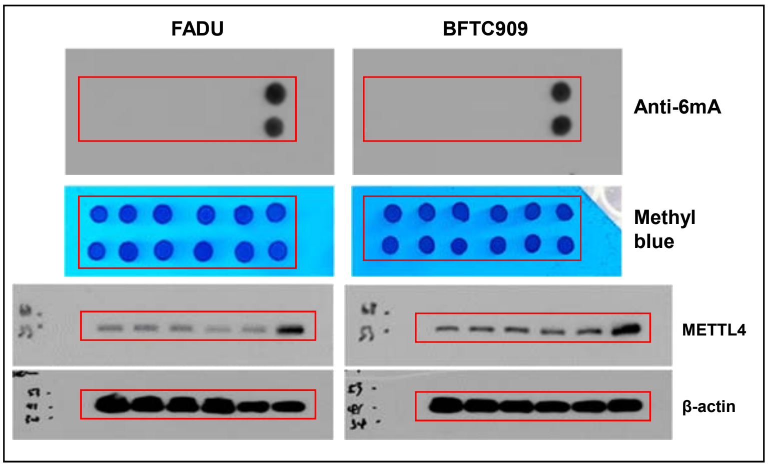

Fig. S1d

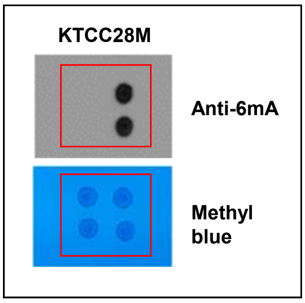

Fig. S1f

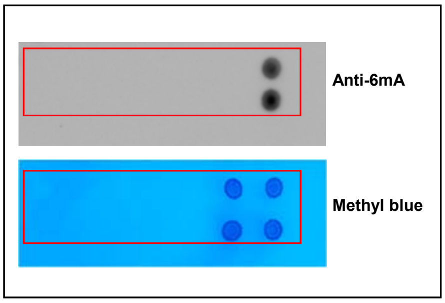

Fig. S1m

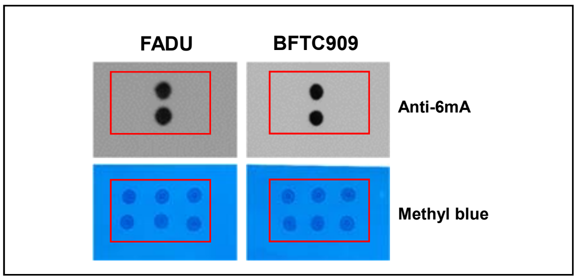

Fig. S1k

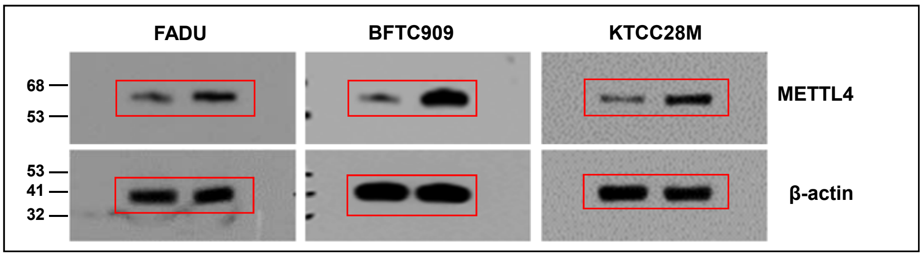

Fig. S1p

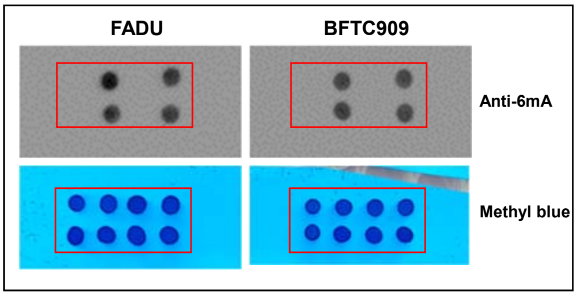

Fig. S1o

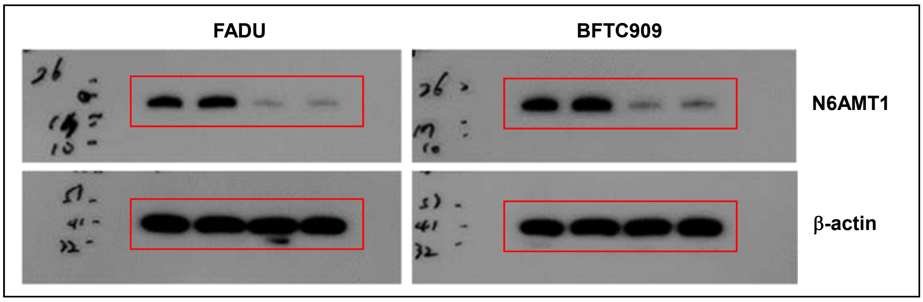

Fig. S1x

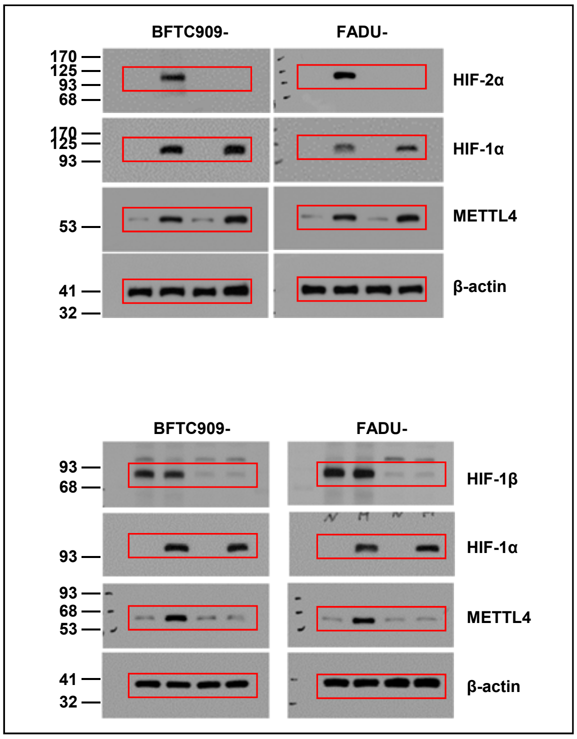

Fig. S1t

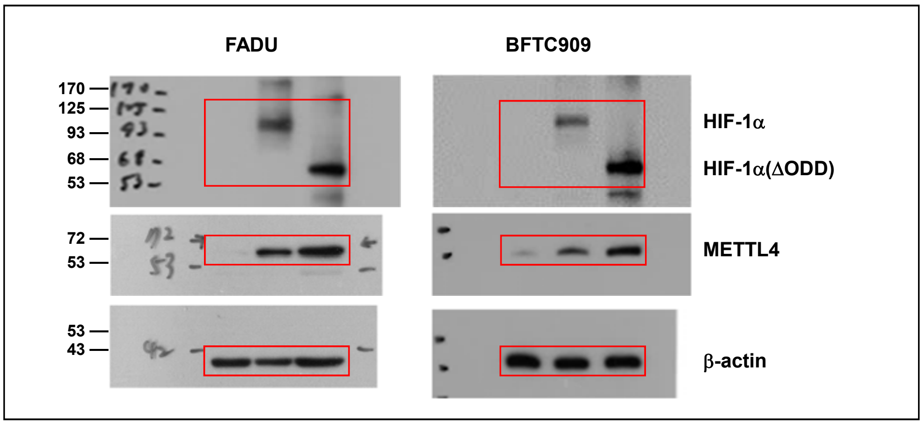

Fig. S1u

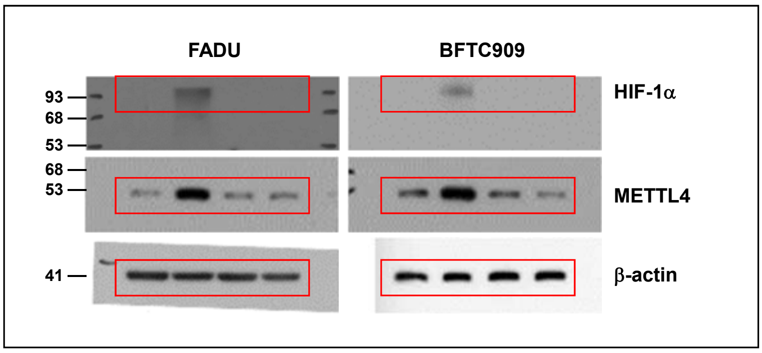

Fig. S1z

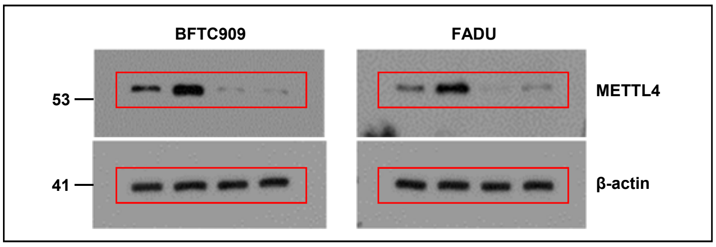

Fig. S2b

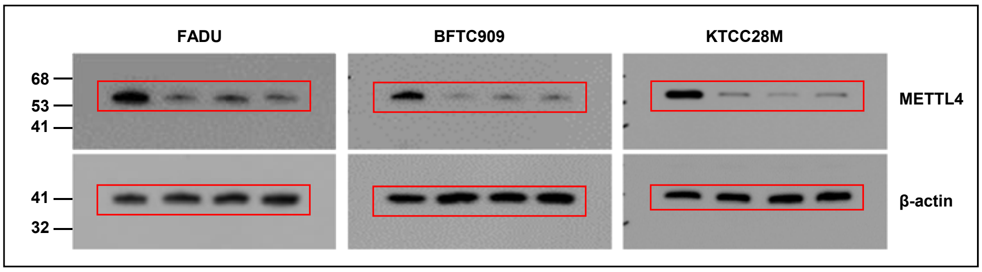

Fig. S2c

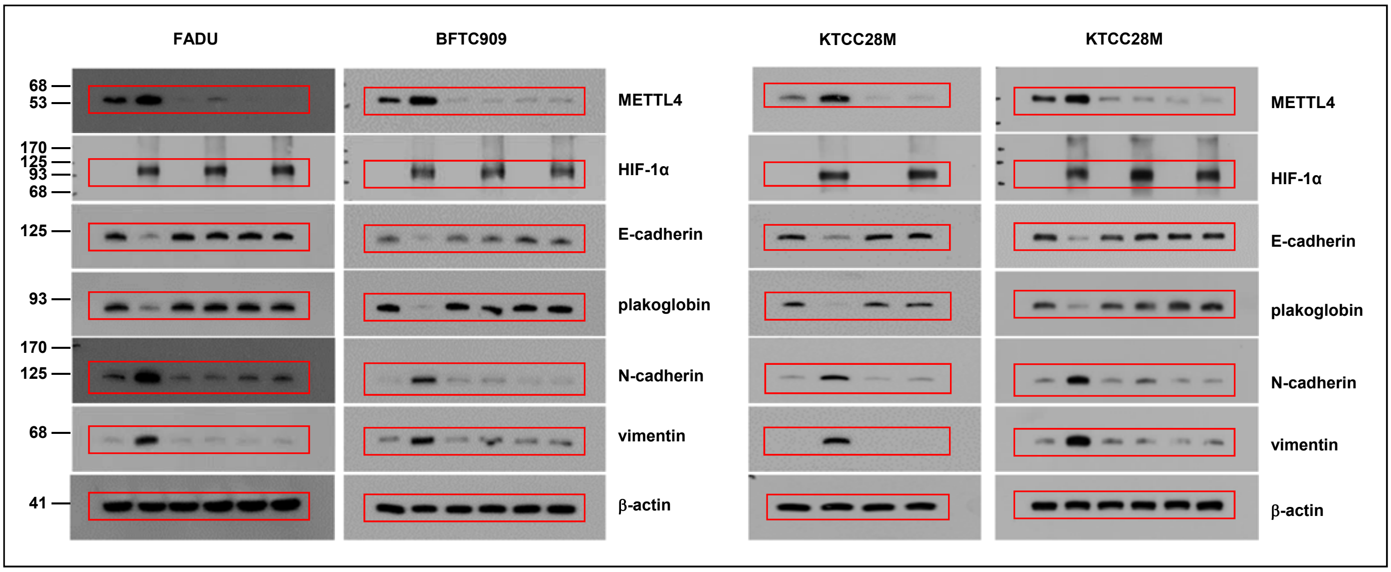

Fig. S2g

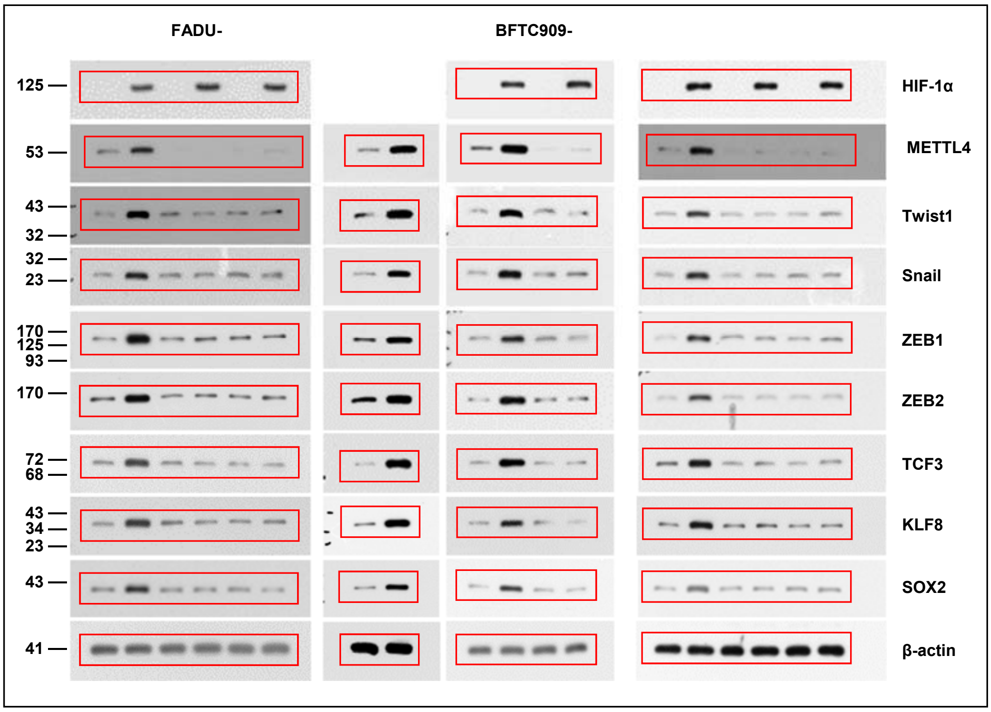

Fig. S2h

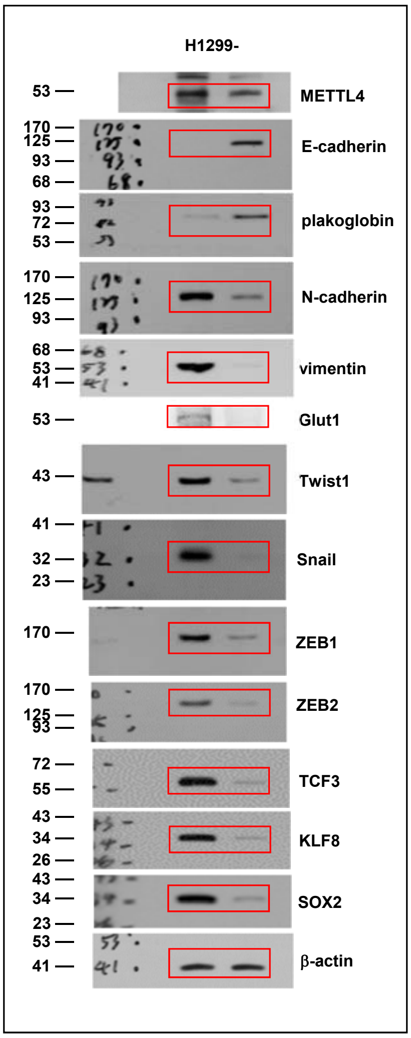

Fig. S2q

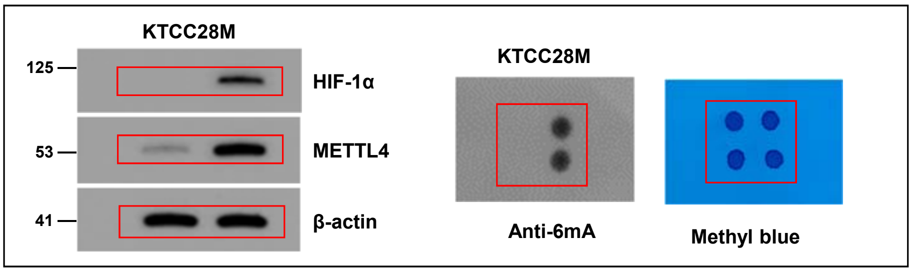

Fig. S3b

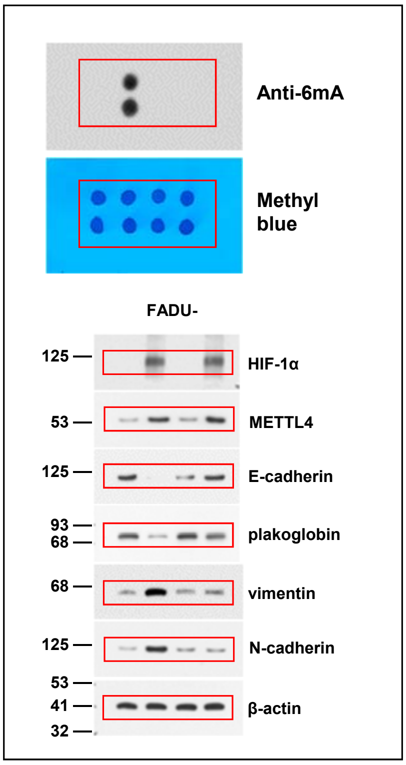

Fig. S3f

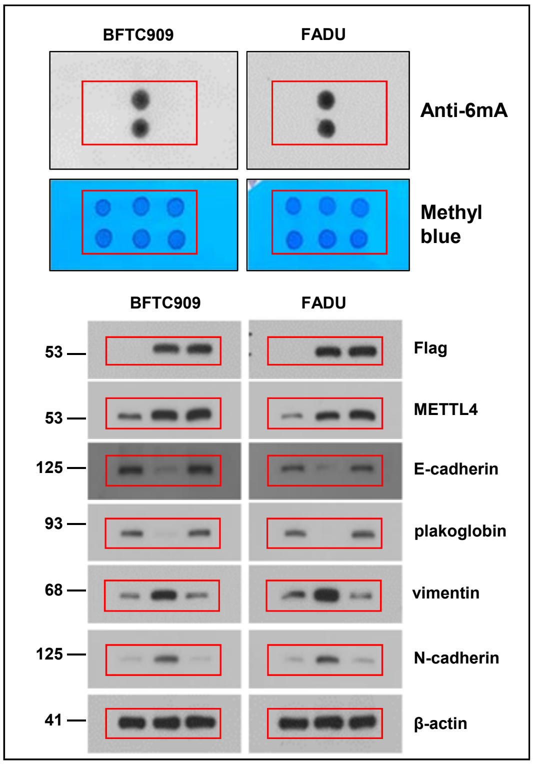

Fig. S3i

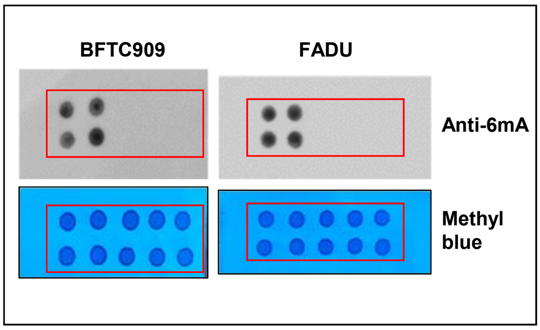

Fig S3m

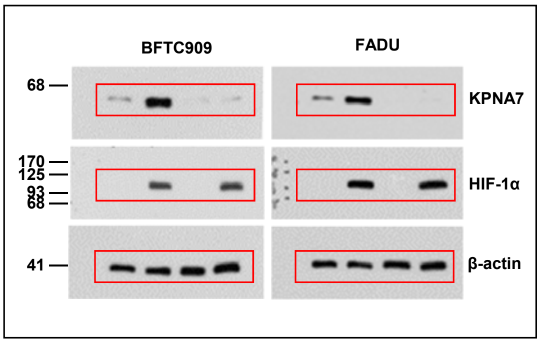

Fig. S3n

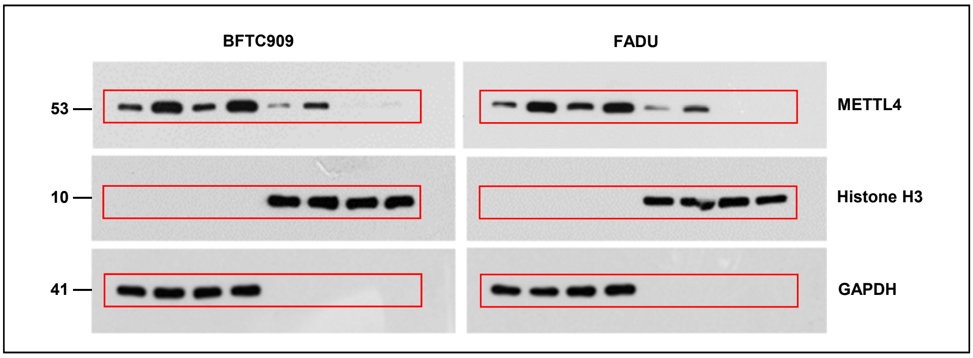

Fig. S4q

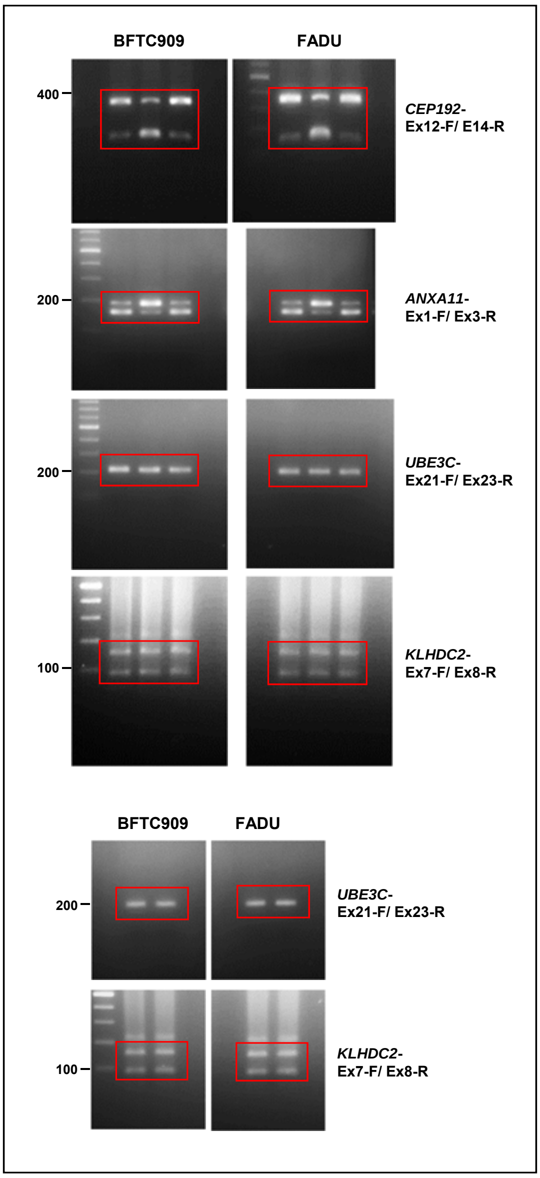

Fig. S4d

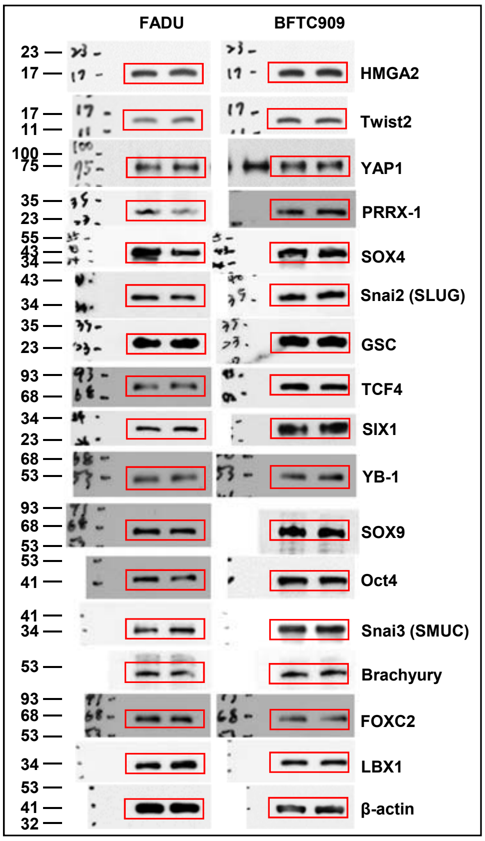

Fig. S4r

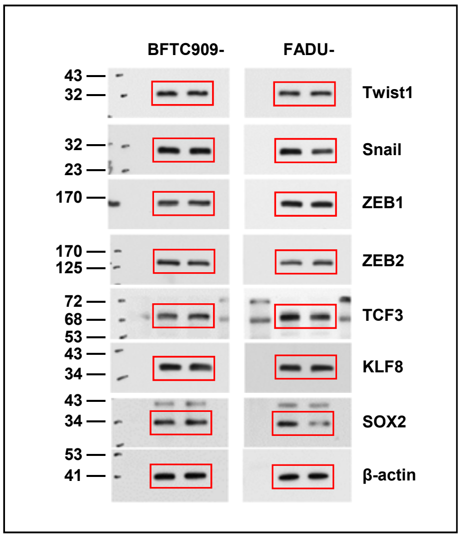

Fig. S4s

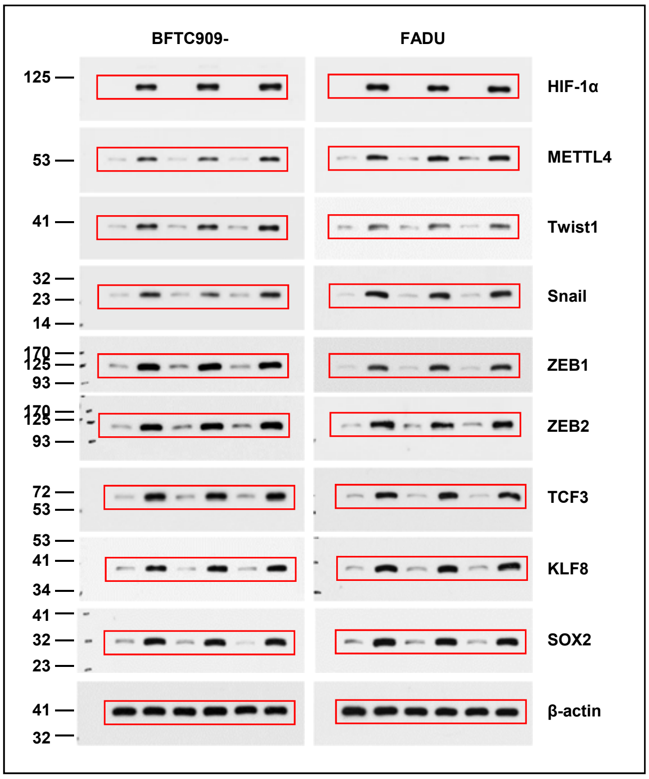

Fig. S4t

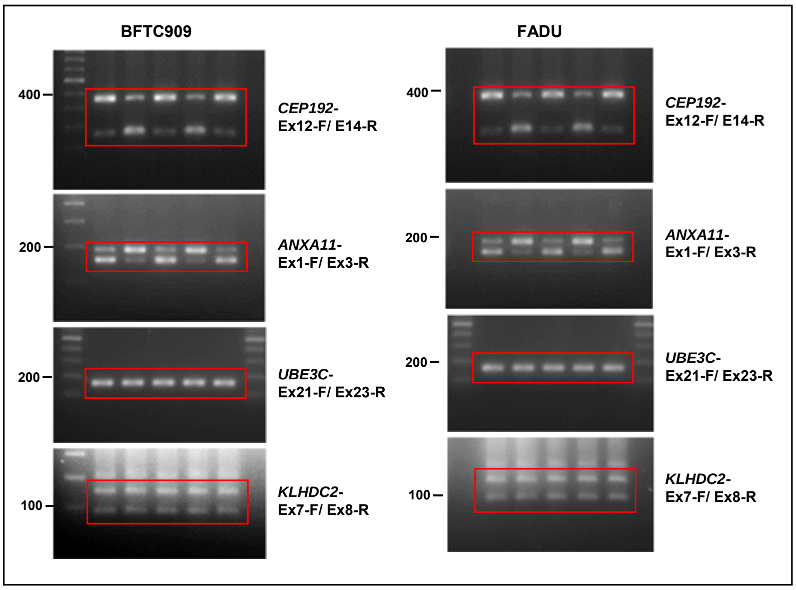

Fig. S4u

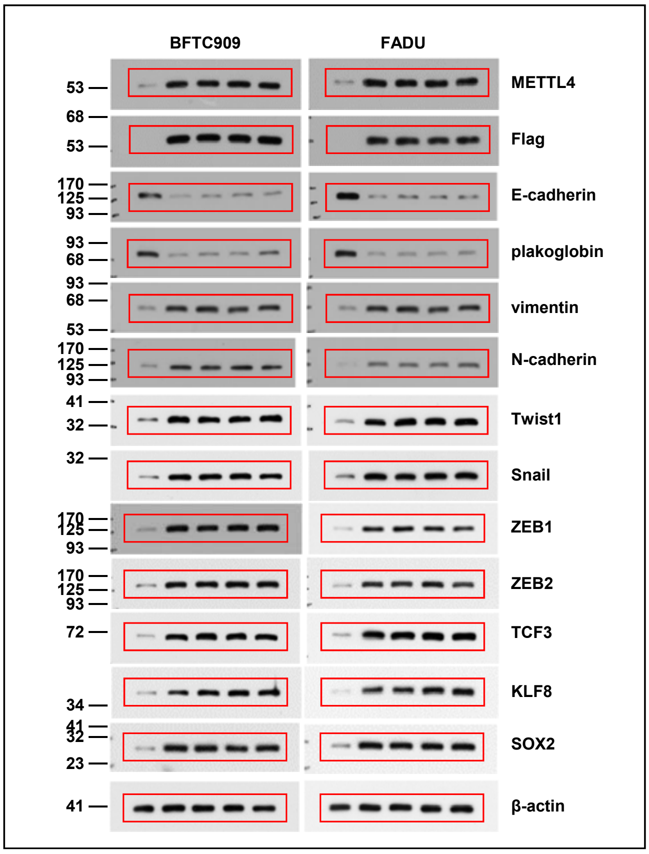

Fig. S6d

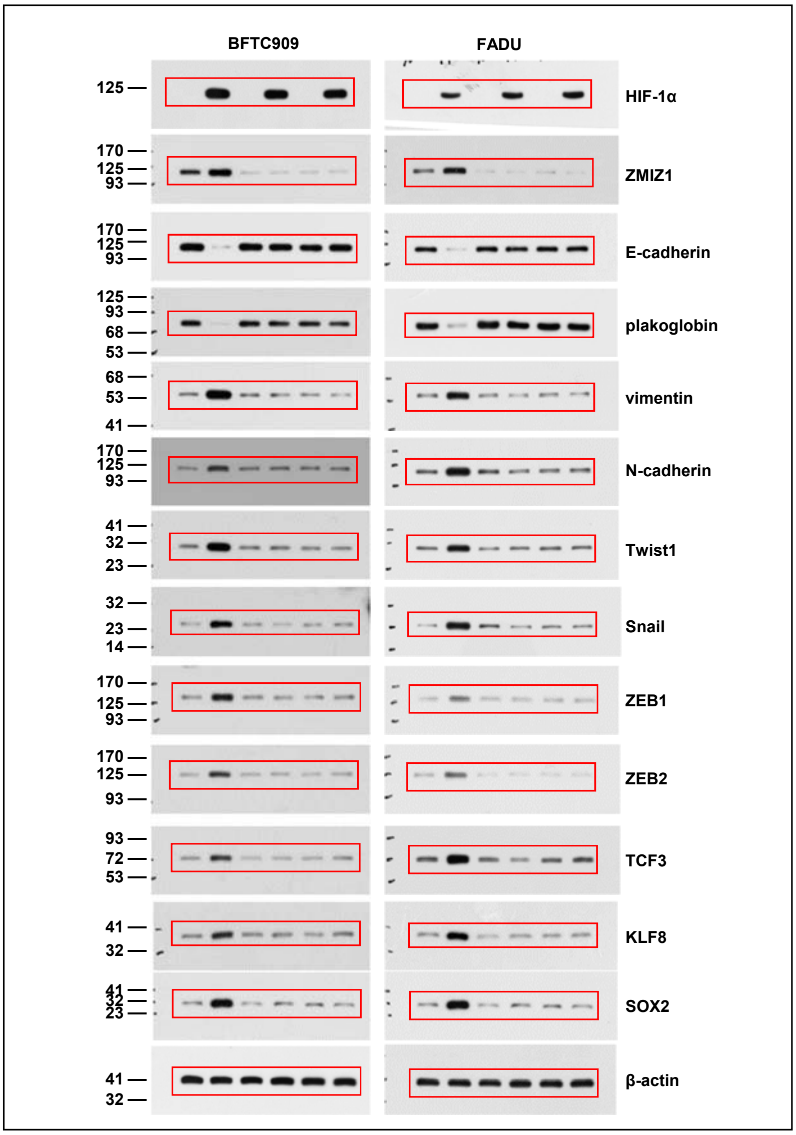

Fig. S6f

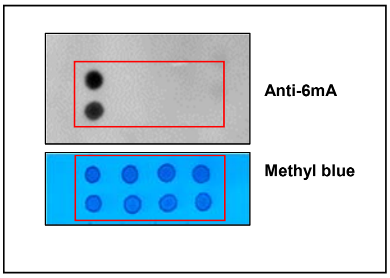

Fig. S6l

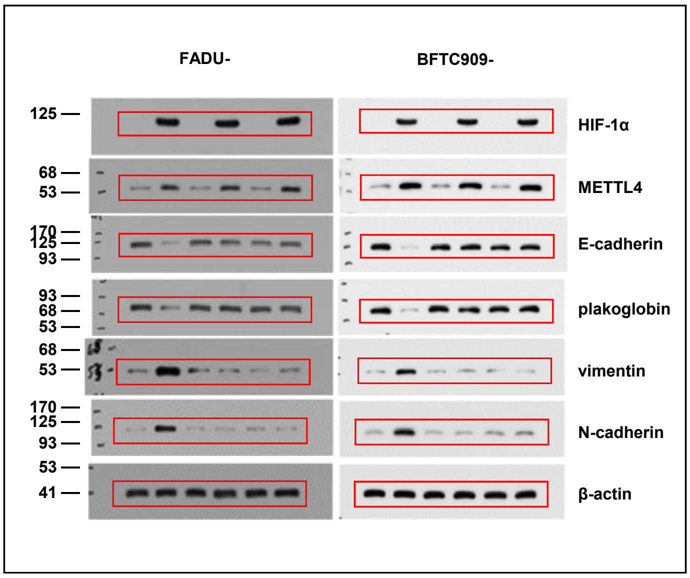

Fig. S6p

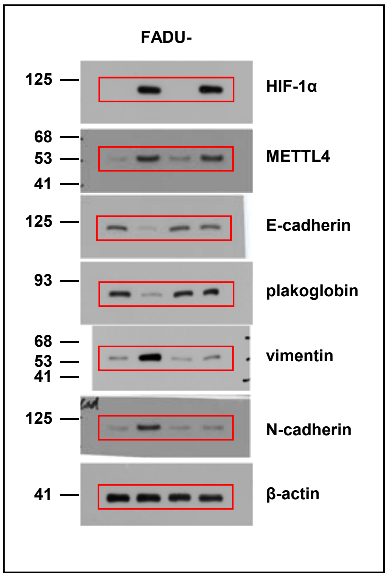

Fig. S6h

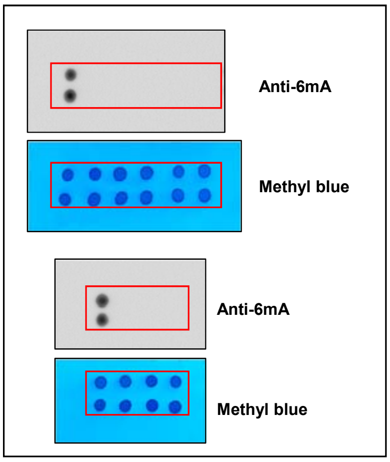

Fig. S6m

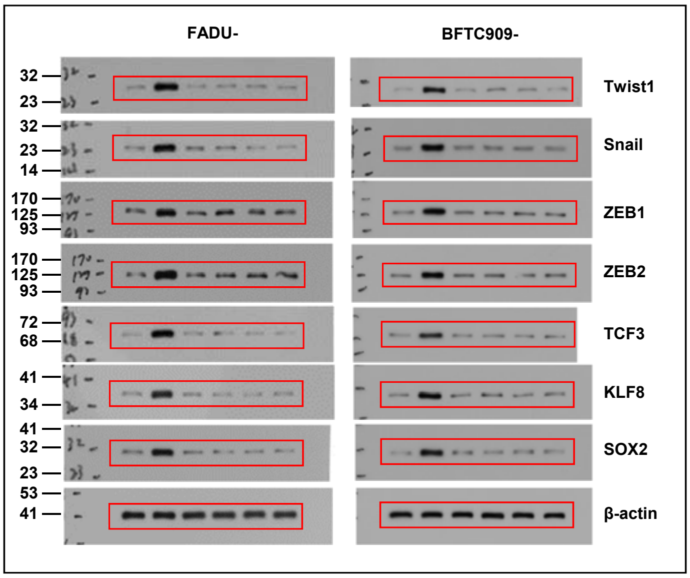

Fig. S6w

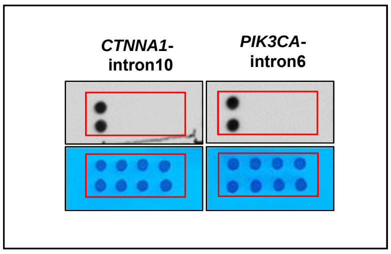

Fig. S6t

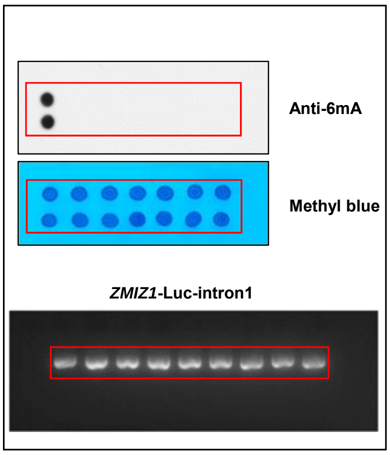

Supplement: Supplementary file 3 — Additional file 3: Contains images of full Western blots. The original uncropped Western blot images used in Figures 1, 2, 3, 4, 5 and 6, Figures S1-4, and Figure S6. [file 13059_2022_2819_MOESM3_ESM.pdf]
