# Supplementary material for: METTL4-mediated nuclear N6-deoxyadenosine methylation promotes metastasis through activating multiple metastasis-inducing targets
Source: Genome Biol. 2022 Dec 2;23:249. doi: 10.1186/s13059-022-02819-3 (PMC9716733; doi:10.1186/s13059-022-02819-3)
Supplement: Supplementary file 4 — Additional file 4: Review history. [file 13059_2022_2819_MOESM4_ESM.docx]

Review History

**First round of review**

**Reviewer 1**

**Were you able to assess all statistics in the manuscript, including the appropriateness of statistical tests used?**

Yes, and I have assessed the statistics in my report.

**Comments to author:**

In this study, Hsu and colleagues investigated the function of Mettl4 and nuclear DNA N6-mA (6mA) deposition in the cancer cell invasion and metastasis in the upper tract urothelial cancer (UTUC). The authors found nuclear DNA 6mA is elevated under the hypoxia condition and the nuclear 6mA methylation was catalyzed by METTL4. Furthermore, METTL4 was demonstrated to be required for the hypoxia-induced epithelial-mesenchymal transition (EMT). For the mechanism, the authors identified that hypoxia-responsive genes were regulated by METTL4 and 6mA. Moreover, LncRNA RP11-390F4.3 and ZMIZ1, which were regulated by the 6mA and METTL4, contribute to the EMT process and cell migration and invasion by activation of HIF1a targeted gene. This study demonstrated that METTL4-mediated nuclear 6mA deposition could induce tumor metastasis. The combination of METTL4 expression and higher 6mA indicates a worse prognostic trend of UTUC patients. Thus, the authors proposed that METLL4 might serve as a potential target for future therapy against UTUC. The whole study is elegant, and the experiments and methods are appropriate to the aims of the study. The authors consider and almost include all necessary controls. This work indeed represents a significant advance in the genomic DNA 6mA studies. This paper will cause broad interest in the field. However, the reviewer has the following concerns or suggestions.

1. The authors should explain the cytosol 6mA signal more clear in the MS and fluorescence staining experiment. For example, Fig. 1b, the cytosol 6mA signal are resistance to RNase digestion, indicating these signals are not from RNA. However, the 6mA signal are much more than Mito tracker in row 4, suggesting it's, at least, not only mtDNA, right? So, are they cytosolic DNA (Fig S1. h)?

2. For the immunofluorescence staining for the nucleotide like 5mC, 5hmC and BrdU, antigen retrieval is an essential step (commonly done with hydrochloric acid), because the common nucleotide antibodies do not recognize its antigen in double-stranded DNA. Specifically, the published study for 6mA staining performed antigen retrieval step by hydrochloric acid (e.g., PMID: 27713410). However, the protocol the authors used does not include this step in the assay. The authors should demonstrate or explain why antigen retrieval is not essential for their experiments/samples for 6mA staining.

3. The authors performed ChIP-exo-seq experiment to profile differential 6mA loci in hypoxia condition vs. normoxia condition, as well as METTL4 KD vs. control under hypoxia condition. Many of the following experiments are based on the ChIP-exo-seq results, so it is essential for the authors to demonstrate the differential 6mA peaks are not generated from 6mA antibody non-specific signals; therefore, the authors should consider providing more evidence regarding this issue. For examples, given the MS results suggested 6mA in normoxia condition is undetectable, the author should be able to estimate the 6mA ChIP-Exo-Seq's FDR in 6mA detection using normoxia condition as a negative control. Also, the conclusion can be further strengthened if the author can generate a WGA negative control sample by whole-genome amplifying genomic DNA under hypoxia condition.

4. In the citation reference 18, Xie, Q., Wu, TP., et al, demonstrated the hypoxia condition, indeed, induce 6mA in GBM genomic DNA and the eraser ALKBH1 can eliminate the 6mA to activate hypoxia responsive genes. So, in this study, authors might need to analyze the down-regulated genes in the RNASeq (hypoxia vs. normoxia) to check what those genes are?

5. Unlike RNA-seq, the authors performed 6mA ChIP-exo-seq only in FADU cells, which was shown to have 2-3-fold lower 6mA abundance than BFTC909 cells under hypoxia condition. The authors might also need to perform 6mA ChIP-exo-seq in BFTC909 cells, or at least conduct the ChIP-qPCR, to confirm the main conclusions.

6. The analyzed data for IgG ChIP-exo-seq control were unavailable at GEO. The author should submit them to GEO.

**Reviewer 2**

**Were you able to assess all statistics in the manuscript, including the appropriateness of statistical tests used?**

Yes, and I have assessed the statistics in my report.

**Comments to author:**

In this study, the authors demonstrated that hypoxia induced upregulation and nuclear translocation of METTL4 thereby increasing nuclear DNA 6mA levels. Consequently, the authors showed that METTL4 is key downstream regulator of HIF-1α mediated EMT in cancer cells and loss of METTL4 greatly hampered in vivo lung metastasis of cancer cells. Furthermore, transcriptome analysis and functional tests revealed that a lncRNA RP11-390F4.3 is a downstream target co-regulated by METTL4 and hypoxia. 6mA ChIP-exo-seq uncovered a group of genes responsible for hypoxia-induced metastasis, including a new co-activator of HIF-1α, which are regulated by METTL4 dependent 6mA. Overall, the authors did a large amount of work and these findings are interesting and novel, but some of the data are not convincing and quality are poor.

Specific comments:

1) The major concern is about immunofluorescence staining results, especially for tissue slides. In Fig 1b, some of the mitochondrial signals from both 6mA and MitoTracker are not clear and more like cytoplasmic. In Fig 1e, METTL4 of mitochondrial location should be observed in both normoxia and hypoxia conditions. In Fig 2h middle panel, these images are clearly background signals as even the DAPI is not from nuclei. Similar issues in supporting information should be addressed.

2) METTL4 mediated 6mA on mtDNA has shown to affect cell proliferation (PMID: 32183942). The authors should check this after METTL4 KD or OE.

3) The quality of some images is poor as the subcellular details are missing, such as Fig S2a, S2d, S2h and S3g.

4) In Fig 2e, some labels are overlapped.

**Authors Response**

**Point-by-point responses to the reviewers’ comments:**

Reviewer #1:

1. The authors should explain the cytosol 6mA signal more clear in the MS and fluorescence staining experiment. For example, Fig. 1b, the cytosol 6mA signal are resistance to RNase digestion, indicating these signals are not from RNA. However, the 6mA signal are much more than Mito tracker in row 4, suggesting it's, at least, not only mtDNA, right? So, are they cytosolic DNA (Fig S1. h)?

*Response: We thank the reviewer for the critical comment. After further immunofluorescence staining experiments, we believe that the previous experiments were just aberrant demonstrations. The new immunofluorescence experiments showed that the cytosol 6mA signals were similar in intensity to the mitotracker signals and these two signals were co-localized (new Fig. 1b, Fig. S1h), supporting that the cytosol 6mA signals belonged to mitochondria as demonstrated (Hao et al., Mol. Cell 78, 382 (2020)). The MS experiments of cytosol 6mA levels were already shown on the previous Mol. Cell paper (Fig. 7 in the Mol. Cell paper). We believe that the IF staining that could simultaneously examine the intensity and location of 6mA signal will better answer the question of cytosol 6mA signals compared to MS experiments.*

2. For the immunofluorescence staining for the nucleotide like 5mC, 5hmC and BrdU, antigen retrieval is an essential step (commonly done with hydrochloric acid), because the common nucleotide antibodies do not recognize its antigen in double-stranded DNA. Specifically, the published study for 6mA staining performed antigen retrieval step by hydrochloric acid (e.g., PMID: 27713410). However, the protocol the authors used does not include this step in the assay. The authors should demonstrate or explain why antigen retrieval is not essential for their experiments/samples for 6mA staining.

*Response: We have re-performed the immunofluorescence staining experiments with or without antigen retrieval. The results showed the similar staining patterns between these two procedures (new Fig. S1g). Therefore, antigen retrieval step appeared not to be essential for the detection of 6mA staining.*

3. The authors performed ChIP-exo-seq experiment to profile differential 6mA loci in hypoxia condition vs. normoxia condition, as well as METTL4 KD vs. control under hypoxia condition. Many of the following experiments are based on the ChIP-exo-seq results, so it is essential for the authors to demonstrate the differential 6mA peaks are not generated from 6mA antibody non-specific signals; therefore, the authors should consider providing more evidence regarding this issue. For examples, given the MS results suggested 6mA in normoxia condition is undetectable, the author should be able to estimate the 6mA ChIP-Exo-Seq's FDR in 6mA detection using normoxia condition as a negative control. Also, the conclusion can be further strengthened if the author can generate a WGA negative control sample by whole-genome amplifying genomic DNA under hypoxia condition.

*Response: We thank the reviewer for the critical comments. The original results presented were already using IgG ChIP-exo-seq results as controls and the non-specific 6mA signals in normoxia condition was used as background signals to subtract from the 6mA signals under hypoxia condition. In the new analyses (comparisons 1 & 2)(Fig. S4i), we further subtracted with whole genome amplified DNA under normoxia condition together with the non-specific 6mA signals in normoxia condition (results shown in Fig. S4i). As suggested by the reviewer, we have performed a WGA negative control experiment and subtracted the background 6mA signals from the original 6mA ChIP-exo-seq 6mA signals in hypoxia condition (Fig. S4j). Indeed the WGA negative control helped to reduce certain backgrounds and the number of 6mA signal-regulated genes were narrowed down from the triple-overlapping results (new Fig. S4i, S4j). The genes containing 6mA signals identified from this approach still had a high percentage of overlapping (>70%) with the genes identified in the original version (data not shown). In addition, the genes that were characterized in the original version (lncRNA RP11-390F4.3, ZMIZ1, CTNNA1, PIK3CA) still had the 6mA signals in the respective locations from the new analysis, indicating the consistency of the newly analyzed 6mA signals. Therefore, the newly analyzed dataset (using different background noises as negative controls) was presented in the revised manuscript. This revision did not affect our original conclusion and the genes analyzed to be regulated by 6mA signals remained the same. Since the negative controls of 6mA signals under normoxia condition and WGA control under hypoxia condition were used, they should most likely minimize the FDR in detecting 6mA signals by using 6mA-ChIP-exo-seq. We believe that the triple-overlapping results (Fig. S4j) should reflect the best 6mA signals that could be identified with the least background.*

4. In the citation reference 18, Xie, Q., Wu, TP., et al, demonstrated the hypoxia condition, indeed, induce 6mA in GBM genomic DNA and the eraser ALKBH1 can eliminate the 6mA to activate hypoxia responsive genes. So, in this study, authors might need to analyze the down-regulated genes in the RNASeq (hypoxia vs. normoxia) to check what those genes are?

*Response: We have analyzed the down-regulated genes in the RNA-seq dataset (hypoxia vs. normoxia). The results showed that indeed a different set of genes were repressed under hypoxia condition and co-regulated by METTL4. The GO and GSEA analysis was shown (Fig. S4g). The signaling pathways in the down-regulated genes (chromatin stability, mitochondria process, RNA metabolism) were different from the up-regulated genes (Fig. 4d).*

*For the results shown in the literature (Xie et al., Cell 175, 1228 (2018)), the data represented the repressed genes under ALKBH1 knockdown which is similar to hypoxia/METTL4 overexpression and hypoxia responsive genes were identified. These results were consistent with our results.*

5. Unlike RNA-seq, the authors performed 6mA ChIP-exo-seq only in FADU cells, which was shown to have 2-3-fold lower 6mA abundance than BFTC909 cells under hypoxia condition. The authors might also need to perform 6mA ChIP-exo-seq in BFTC909 cells, or at least conduct the ChIP-qPCR, to confirm the main conclusions.

*Response: We thank the reviewer for the comment. Using the newly analyzed 6mA signal regions and genes from the newly analyzed sequencing datasets, we have conducted MeDIP (methylation DNA immunoprecipitation) assays to further analyze 5 more genes that contained putative 6mA peaks. RNA qRT-PCR result showed that these genes were activated by hypoxia/METTL4 overexpression (new Fig. S6x). In addition, the 6mA signals identified in these genes were confirmed by MeDIP experiments to show the presence of 6mA peaks on the corresponding genomic 6mA signals in their promoter or intronic regions in BFTC909 and FADU cells (new Fig. S6z). This result supported our conclusion that our ChIP-exo-seq method (through new analysis) was able to identify genes containing 6 mA signals on their genomic regions with corresponding regulation of gene expression.*

6. The analyzed data for IgG ChIP-exo-seq control were unavailable at GEO. The author should submit them to GEO.

*Response: The IgG ChIP-exo-seq control was submitted to GEO. Newly performed WGA 6mA ChIP-seq data were also submitted. Please see the method section for accession numbers.*

Reviewer #2:

Specific comments:

1) The major concern is about immunofluorescence staining results, especially for tissue slides. In Fig 1b, some of the mitochondrial signals from both 6mA and MitoTracker are not clear and more like cytoplasmic. In Fig 1e, METTL4 of mitochondrial location should be observed in both normoxia and hypoxia conditions. In Fig 2h middle panel, these images are clearly background signals as even the DAPI is not from nuclei. Similar issues in supporting information should be addressed.

*Response: We thank the reviewer for the critical comments. After further immunofluorescence staining experiments, we believe that the previous experiments were just aberrant demonstrations. The new immunofluorescence experiments showed that the cytosol 6mA signals were similar in intensity to the mitotracker signals and these two signals were co-localized (new Fig. 1b, Fig. S1h), supporting that the cytosol 6mA signals belonged to mitochondria as demonstrated (Hao et al., Mol. Cell 78, 382 (2020)).*

*We re-performed the experiments for Fig. 1e and added the mitotracker staining experiment (new Fig. 1e, Fig. S1l). The results showed that METTL4 of mitochondrial location was observed in both normoxia and hypoxia conditions, in which the METTL4 signals in hypoxia condition were significantly increased compared to the METTL4 signals in normoxia condition.*

*We have re-performed DAPI staining together with staining of other markers. The results showed that the DAPI staining contours were consistent with the staining of the other two markers (new Fig. 2h, S2r) and we believe the imaging has been significantly improved.*

2) METTL4 mediated 6mA on mtDNA has shown to affect cell proliferation (PMID: 32183942). The authors should check this after METTL4 KD or OE.

*Response: We performed METTL4 KD experiments and the cell proliferation rate in three cell lines decreased. Consistently, we performed METTL4 OE experiments and the cell proliferation rate in three cell lines increased. Furthermore, we performed METTL4 knockdown followed by reconstitution with a wild type METTL4 or a NLS-mutated METTL4. The results showed that reconstitution with a wild type METTL4 could fully rescue the cell proliferation rate, whereas reconstitution with a NLS-mutated METTL4 only partially rescued cell proliferation rate (new Fig. S2k-m). These results indicated that the cytoplasmic and nuclear function of METTL4 played a certain role in mediating cell proliferation.*

3) The quality of some images is poor as the subcellular details are missing, such as Fig S2a, S2d, S2h and S3g.

*Response: We have re-performed the imaging experiments in Fig. S2a, S2d, S2h, and S3g. We believe the quality has been significantly improved.*

4) In Fig 2e, some labels are overlapped.

*Response: We have re-labeled all the overlapping labels to make sure there are no overlappings.*

**Second round of review**

**Reviewer 1**

The authors have answered all the questions and addressed all the issues in this revision.

**Reviewer 2**

The authors have addressed all my concerns in the revised manuscript. I think the manuscript is up to the standard for publication in Genome Biology.
